# Effects of the COVID-19 pandemic and previous pandemics, epidemics and economic crises on mental health: systematic review

**DOI:** 10.1192/bjo.2022.587

**Published:** 2022-10-10

**Authors:** Michaela Asper, Walter Osika, Christina Dalman, Elin Pöllänen, Otto Simonsson, Pär Flodin, Anna Sidorchuk, Laura Marchetti, Fatima Awil, Rosa Castro, Maria E. Niemi

**Affiliations:** Department of Global Public Health, Karolinska Institutet, Sweden; Centre for Psychiatry Research, Department of Clinical Neuroscience, Karolinska Institutet, Sweden; and Stockholm Health Care Services, Region Stockholm, Sweden; Department of Clinical Neuroscience, Karolinska Institutet, Sweden; Mental Health Europe, Belgium; Federation of European Academies of Medicine, Belgium

**Keywords:** COVID-19, suicide, depressive disorders, anxiety disorders, epidemiology

## Abstract

**Background:**

A rise in mental illness is expected to follow the COVID-19 pandemic, which has also been projected to lead to a deep global economic recession, further adding to risk factors.

**Aims:**

The aim of this review was to assess the impact of the COVID-19 pandemic and previous pandemics, epidemics and economic crises on mental health.

**Method:**

Searches were conducted in PubMed, Web of Science, PsycINFO and Sociological Abstracts. We included studies of all populations exposed to the COVID-19 pandemic, and other similar pandemics/epidemics and economic crises, compared with non-exposed time periods or regions. The outcome was mental health.

**Results:**

The 174 included studies assessed mental health impacts of the COVID-19 pandemic (87 studies), 2008 economic crisis (84 studies) and severe acute respiratory syndrome (SARS) epidemic (three studies). Outcomes were divided into affective disorders, suicides, mental healthcare utilisation and other mental health. COVID-19 pandemic studies were of lesser quality than those for the economic crisis or SARS epidemic. Most studies for all exposures showed increases in affective disorders and other mental health problems. For economic crisis exposure, increases in mental healthcare utilisation and suicides were also found, but these findings were mixed for COVID-19 pandemic exposure. This is probably because of quarantine measures affecting help-seeking and shorter follow-ups of studies of COVID-19 pandemic exposure.

**Conclusions:**

Our findings highlight the importance of available, accessible and sustainable mental health services. Also, socioeconomically disadvantaged populations should be particular targets of policy interventions during the COVID-19 pandemic.

The COVID-19 pandemic has had profound effects on population health, resulting from both actual COVID-19 infection and collateral effects of the pandemic.^1^ A rise in mental illness was expected to follow the pandemic, caused by COVID-19-related factors such as fear, bereavement, social isolation and socioeconomic impact.^2^ Also, many people were projected to experience increased levels of alcohol and drug use, insomnia and anxiety.^3^ Furthermore, the COVID-19 pandemic has contributed to the largest global economic shock in decades.^4^ Therefore, the impact of economic recessions on mental health and well-being^5^ may further contribute to the negative effects of the pandemic. Indeed, negative mental health effects from previous epidemics and economic crises have also been reported.^5–7^

Collecting high-quality data on the mental health effects of the COVID-19 pandemic has therefore been identified as an immediate research priority, and international comparisons will be especially helpful in this regard.^2^ The aim of this report is to systematically review the impact that the COVID-19 pandemic has had on mental health, and provide information about possible effects that may add to this as a result of an eventual economic crisis following the pandemic. Therefore, we intend to map information on the impact of previous pandemics/epidemics similar to COVID-19, and the impact of earlier economic crises, to guide the prevention and management of negative mental health effects following the COVID-19 pandemic.

## Method

The searches were designed in collaboration with a university librarian, and conducted on 6 January 2021 in PubMed, Web of Science, PsycINFO and Sociological Abstracts (see search strings in Supplementary Appendix 1 available at https://doi.org/10.1192/bjo.2022.587). The searches were restricted to the years 2000–2021 and the English language, and reference lists of systematic reviews were scanned.

Inclusion criteria were as follows:

- population: general population and/or any specific populations;
- exposure: COVID-19 or pandemics and epidemics similar to COVID-19 (Middle East respiratory syndrome, severe acute respiratory syndrome (SARS), H1N1 influenza (swine flu)), or economic crises (see search strings in Supplementary Appendix 1 for details);
- comparator: pre-pandemic/epidemic or pre-crisis measures or unaffected geographical areas;
- outcome: mental health outcomes (see search strings in Supplementary Appendix 1 for details);
- type of study: longitudinal cohort and repeated cross-sectional studies.

### Study selection and data extraction

The titles and abstracts were independently screened by two researchers, in pairs (M.A., E.P., W.O., O.S., P.F., M.E.N., R.C., L.M. and F.A.). Disagreement was resolved through discussion among the pair or by consulting a third researcher within the team. Articles included for full-text screening were assessed against the inclusion criteria by two researchers. This review followed the Preferred Reporting Items for Systematic Reviews and Meta-Analyses (PRISMA) guidelines,^8^ and the review protocol has been pre-registered in the International Prospective Register of Systematic Reviews (PROSPERO; identifier CRD42021252774; available from https://www.crd.york.ac.uk/prospero/display_record.php?RecordID=252774). The data were collected by one researcher (M.A., E.P., O.S., P.F., M.E.N., R.C., L.M., F.A. or C.D.). The extracted data were then checked by another researcher (M.A. or M.E.N.).

### Risk-of-bias quality assessment

The quality of the included studies was assessed with the Newcastle–Ottawa Scale,^9^ and the assessment ratings for each individual study can be found in the table in Supplementary Appendix 2. The assessment was done independently by two researchers (M.A. and M.E.N.); disagreement was resolved by discussion between them. The study quality was defined as high (7–9 points), fair (5–6 points) or low (≤4 points).

### Qualitative synthesis and harvest plots

Because of the large variation in outcomes measures reported in the included studies (relative risk, mean score, *P*-values only, frequencies, no numerical data in the results reported, etc.), we chose to conduct a qualitative synthesis instead of a meta-analysis, as recommended in the literature.^10,11^ Graphical display of the directions of association across multiple variables is recommended for qualitative synthesis,^10^ and we have therefore visualised the direction of associations between the exposures and outcomes of interest in harvest plots in Figs 2–4.^12^ Further, we performed a grouping by potential moderators: study setting (the country of study origin, further combined into geographical regions) and study size (subdivided into the smaller studies with <1000 participants, medium-sized studies with 1000–10 000 participants and larger studies with >10 000 participants). The grouping by study size mirrors an assessment of a ‘small-study effect’ (i.e. if significant associations are found mainly in small underpowered studies, compared with the results of larger studies),^13^ which is indicative of publication bias.

## Results

**Fig. 1:**
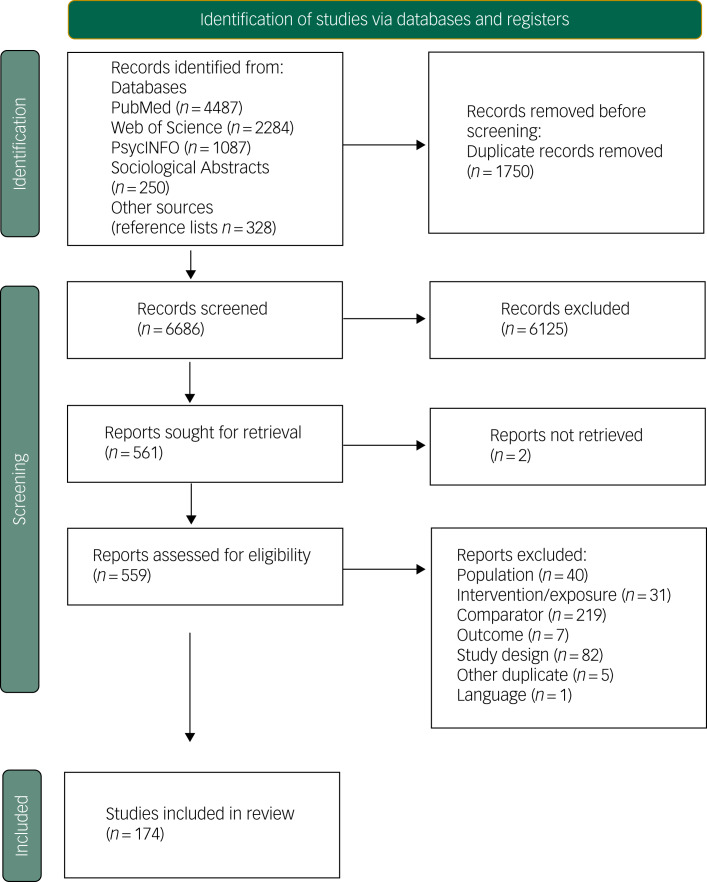
Preferred Reporting Items for Systematic Reviews and Meta-Analyses (PRISMA) 2020 flow diagram for new systematic reviews, including searches of databases and registers.

Figure 1 shows the results of the selection process. We screened 6686 studies by title and abstract. The full texts of 559 studies were assessed for eligibility, and 174 studies met our selection criteria and were included. Articles excluded at the full-text stage are listed in Supplementary Appendix 3, with reasons for exclusion.

Details about the included studies are given in Tables 1–3 in Supplementary Appendix 4. A qualitative summary of the findings is provided below, divided by type of exposure (COVID-19, economic crises or SARS) and outcome (affective disorders, suicides, other mental health problems and healthcare utilisation). For each exposure–outcome combination, the summary presents the direction of reported associations as well as study populations and settings.

### COVID-19 exposure

Altogether, 87 studies were included assessing mental health impacts of the COVID-19 pandemic, where 43 focused on affective disorders, four assessed suicides, 30 assessed other mental health outcomes and ten examined mental healthcare utilisation.

#### Affective disorders

**Fig. 2:**
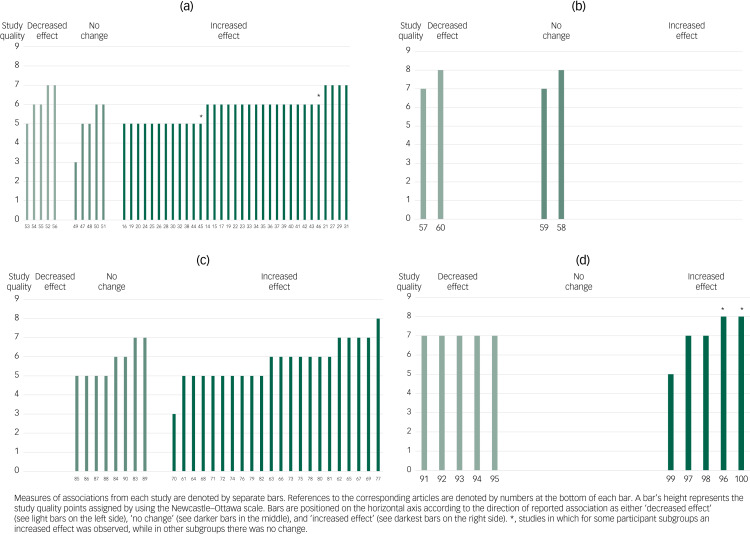
Harvest plot for the associations reported between exposure to the COVID-19 pandemic and (a) affective disorders, (b) suicides, (c) other mental health outcomes and (d) healthcare utilisation. Labels on the x-axis refer to the reference list entries for the studies.

Among the studies on affective disorders (Fig. 2(a)), 31 found increases during the COVID-19 pandemic^14–44^ and two found increases in subgroups of participants.^45,46^ These were conducted on population-based samples (151–336 52 participants);^17,18,22,29,33,39,40,43^ more specific healthy populations of various ages, life stages or occupations (93–7527 participants);^14,15,21,23–27,34,36–39,41,42^ and patients/populations with various somatic or psychiatric diagnoses (46–1 854 742 participants).^16,19,20,28,30–32,35^ The studies were conducted in Hong Kong,^26,33^ the USA,^15,17–19,22,24,30,34,40,42^ the UK,^21,29,30,34,39^ Germany,^31,43^ China,^36,37^ Italy,^14,20,25^ Australia,^32,38^ Bangladesh,^23^ India,^27^ Switzerland,^44^ South Sudan,^41^ Canada,^30^ France,^30^ Singapore,^35^ Serbia^28^ and The Netherlands.^16^ Among these studies, four were of high quality^21,27,29,31^ and 27 were of fair quality.^14–20,22–26,28,30,32–44^ A fair-quality study of adolescents and parents from The Netherlands found increased negative affect only in parents,^45^ and a fair-quality study of people with cancer from the UK found increased rates of depression only among those with certain cancer types.^46^

Altogether, five studies with more defined samples of various ages, occupations and health conditions, with 25–3983 participants, found no change in affective disorders. They were conducted in Canada^47^ the USA^48,49^ The Netherlands^50^ and Italy.^51^ Four of these studies were of fair quality,^47,48,50,51^ and one was of low quality.^49^

Some studies found unchanged or lower rates of affective disorders,^52–55^ and lower incidence of medication prescriptions.^56^ These were conducted on populations of 164–241 458 participants, including postpartum women in Israel,^52^ patients in general practice in the UK,^56^ medical students from the Republic of Kazakhstan,^54^ patients from a sleep clinic from Japan^53^ and university students in China.^55^ Three of these studies were of fair quality^53–55^ and two were of high quality.^52,56^

#### Suicides

Four studies assessed pandemic-period suicide rates in whole populations from Connecticut (USA),^57^ Queensland (Australia),^58^ Japan^59^ and Peru,^60^ and found these had either decreased^57,60^ or remained unaltered (Fig. 2(b)).^58,59^ All four studies were of high quality.

#### Other mental health outcomes

There were 30 studies that assessed other mental health outcomes (Fig. 2(c)). Altogether, 12 studies were conducted on population-based samples and found decreases in mental health.^61–72^ These studies were conducted on populations ranging from 1003 to 17 452 individuals in the USA,^61,67,68^ the UK,^62–65,69^ New Zealand,^66^ Denmark,^70^ Canada^71^ and China.^72^ Four of these studies were of high quality,^62,65,67,69^ seven were of fair quality^61,63,64,66,68,71,72^ and one was of low quality.^70^

Ten other studies in more defined samples ranging from 21 to 3505 individuals also found deteriorations in mental health.^73–82^ These included populations of different ages and occupations,^73–76,78,79,82^ and patients with various somatic or psychiatric diagnoses,^77,80–82^ and were conducted in the USA,^77,78,82^ Spain,^74^ Switzerland,^75^ Croatia,^79^ the UK^73,76^ and Italy.^80,81^ One was of high quality^77^ and nine were of fair quality.^73–76,78–82^

Eight of the studies did not find changes in mental health among study populations of 46–1870 participants. These populations were of various ages and occupations, both healthy and with somatic or mental health diagnoses, conducted in the USA,^83–86^ Sweden,^87^ Germany^88^ and The Netherlands.^89,90^ Of these studies, two were of high quality^83,89^ and six were of fair quality.^84–88,90^

#### Healthcare utilisation

Figure 2(d) presents a harvest plot for associations between COVID-19 and healthcare utilisation. Altogether, five studies that assessed admissions for mental health problems found decreases: emergency department presentations decreased at three health services in Australia^91^ and two hospitals in Italy;^92,93^ psychiatric emergency services presentations decreased in Paris, France;^94^ and presentations to a paediatric emergency department decreased in the USA.^95^ All of these five studies were of high quality.

On the other hand, although acute care presentations for mental health diagnoses in the UK decreased, the patients admitted had more severe conditions.^96^ Admissions for mental health problems increased at an acute medical unit,^97^ and there was acceleration in urgent referrals to secondary mental health services in the UK.^98^ In Italy, psychological morbidity worsened among 145 palliative care professionals.^99^ An emergency department in New Zealand experienced overall decreases in mental health presentations, but relative increases in overdoses and self-harm.^100^ Four^96^^–^^98,100^ of these studies were of high quality and one was of fair quality.^99^

### Economic crisis exposure

Altogether 84 studies were included assessing mental health impacts of the 2008 economic crisis. Among these, 15 studies focused on affective disorders, seven assessed mental healthcare utilisation, 37 assessed suicides and 25 assessed other mental health outcomes.

#### Affective disorders

**Fig. 3:**
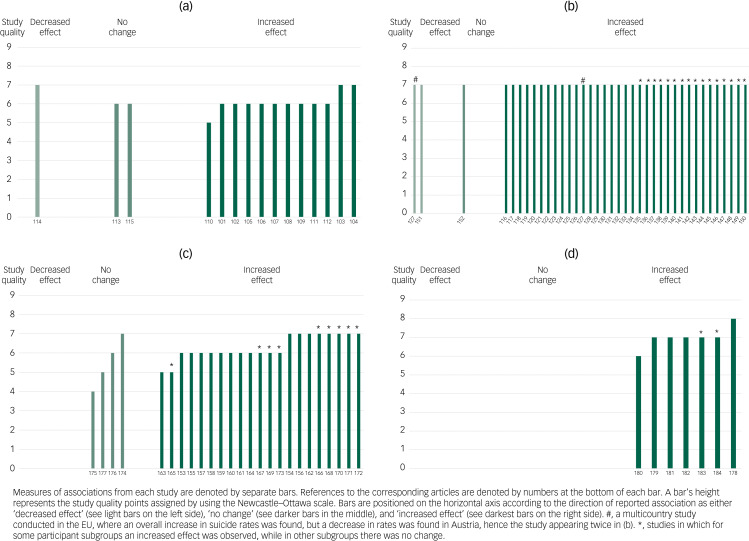
Harvest plot for the associations reported between exposure to the economic crisis and (a) affective disorders, (b) suicides, (c) other mental health outcomes and (d) healthcare utilisation. Labels on the x-axis refer to the reference list entries for the studies.

Figure 3(a) presents a harvest plot for associations between economic crises and affective disorders. All 15 studies reporting affective disorders as an outcome were population-based surveys. The findings from 12 of these studies, with populations ranging from 2011 to 81 313 participants, were that there was a significant increase in affective disorders.^101–112^ These studies were conducted in Canada,^101^ Hong Kong,^102^ the USA,^103–105,108–111^ Europe,^103^ Spain^106^ and Australia.^107,112^ Two of these studies were of high quality^103,104^ and ten were of fair quality.^101^^,^^102^^,^^105–112^

A study on 815 adults aged over 50 years found no increase in depression among those most affected by the stock market crash, despite an increase in antidepressant medication use.^113^ Also, a study on 25- to 75-year-olds in the USA found that mental health improved.^114^ Among 106 158 participants aged over 15 years from 21 European countries, no effect of the crisis was found on depressive feelings.^115^ One study was of high quality,^114^ and two were of fair quality.^113,115^

#### Suicides

Altogether, 37 studies assessed suicide in relation to the 2008 economic crisis, and all of these studies were of high quality (Fig. 3(b)). Altogether, 19 studies found increased suicide rates at the level of the total population after the start of the crisis. These were conducted on the populations of Italy (Milan)^116^ (suicides as a result of mental and behavioural disorders, Italy^117^), Greece,^118–123^ Spain^124^ (suicide attempts in Spain^125^), the European Union,^126–128^ Canada,^126^ England,^128–130^ the USA^131–133^ and South Korea.^134^

Some studies reported increases in suicide rates in specific population subgroups,^135,136^ among men^137–139^ or attributable to specific factors such as unemployment.^140–145^ These studies were conducted in Greece,^135,136^ Italy,^140^ Australia,^141^ Spain,^138,142,143^ Barcelona (Spain),^144^ the UK,^137^ Ireland^139^ and the USA.^136,145^ A study from 29 countries in the European Union found a general relationship between the economic environment and suicide rates.^146^

A study conducted on the male population of 20 countries in the European Union found job losses to be a determinant of suicide risk, and greater spending on active labour market policies and social capital mitigated risks.^147^ A study from 27 European countries, 18 North and South American countries, eight Asian countries, and one African country found that suicide rates increased in the European and North and South American countries, particularly in men and in countries with higher levels of job loss.^148^ In Italy, periods of economic fluctuations were associated with male suicides, whereas severe economic downturns were associated with increased rates overall,^149^ and gross domestic product was associated with suicides because of financial problems.^150^

Finally, one study in Piraeus, Greece, found a slight decrease in suicide rates,^151^ and a study including all European Union countries found decreased rates in Austria.^127^ Also, a study in Crete, Greece, found no overall increase in suicide rates.^152^

#### Other mental health outcomes

Most of the 25 studies assessing other mental health outcomes (Fig. 3(c)) were conducted on nationally or regionally representative samples, and the clear majority found evidence for increased mental distress.^153–162^ The studies that presented results at the population level included 3479–306 664 participants from Sweden,^153^ the UK,^155^ Italy,^156^ Spain,^157^ England,^154,159^ Australia,^160^ Iceland,^162^ the Valencian Community in Spain^158^ and 36 mainly European countries.^161^ Three of these studies were of high quality^154,156,162^ and seven were of low quality.^153,155,157–161^ Also, two studies on more defined populations of 2050 medical researchers in Greece,^163^ and 13 000 children aged 4–17 years in the USA,^164^ found decreases in mental health. Both studies were of fair quality.

Some of the population-based studies, ranging from 3755 to 11 743 participants, showed decreases in mental health only among particular population groups,^165–171^ or under higher rates of precarious employment and lower health spending. These studies were conducted in Spain,^167,169,171^ Ireland,^165^ Iceland,^166^ France^168^ and the UK.^170^ In the USA, retail sales for angiotensin-converting enzyme inhibitors and selective serotonin reuptake inhibitors/serotonin–noradrenaline reuptake inhibitors were not associated with unemployment, but there were positive associations for opioids and phosphodiesterase inhibitors.^172^ Five of these studies were of high quality^166,168,170–172^ and three were or fair quality.^165,167,169^

Also, one study with a cohort of 3321 mothers and 4089 children in Australia found that girls experienced an increase in mental health problems, but not boys or mothers.^173^ This study was of fair quality.

Four studies found no changes in mental health outcomes. They were conducted on a population-based sample in the UK;^174^ a nationally representative sample of adults aged over 50 years in Ireland;^175^ a study of 21 European countries;^176^ and a study of children aged 11–15 years from Israel, the USA and 31 countries in Europe.^177^ One of these studies was of high quality,^174^ two were of fair quality^176,177^ and one was of low quality.^175^

#### Healthcare utilisation

Figure 3(d) presents a harvest plot for economic crises and healthcare utilisation. Five of the seven studies assessing changes in healthcare utilisation for mental health problems found increases in rates. They addressed in-patient admissions for affective disorders in Italy,^178^ hospital admissions owing to depression in Taiwan,^179^ primary care patients in Spain,^180^ general practice patients in the UK^181^ and hospital morbidity data in Spain.^182^ Four studies were of high quality^178,179,181,182^ and one was of fair quality.^180^

Two studies did not find overall increases in mental healthcare utilisation: in the UK, rates of self-harm among patients increased in Derby and among males in Manchester, but not in in Oxford;^183^ in the USA, physician visits owing to mental health disorders decreased after the onset of the crisis, but the use of psychotropic medications increased.^184^ Both of these studies were of high quality.

### SARS exposure

**Fig. 4:**
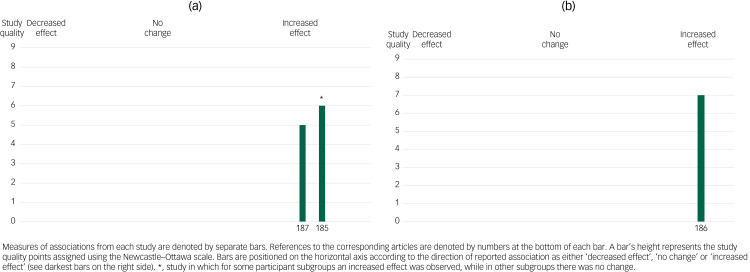
Harvest plot for the associations reported between exposure to the severe acute respiratory syndrome (SARS) epidemic and (a) other mental health outcomes and (b) suicides.Labels on the x-axis refer to the reference list entries for the studies.

Our review also yielded three studies addressing changes in mental health before and after the onset of the SARS epidemic in Hong Kong (Fig. 4(a) and (b)). All of these studies were conducted on adults of older age.^185–187^ One study based on a stratified random sample showed no changes in depression among men, but an increase in depression among women. Another study found an excess in suicide rates among older adults.^186^ Finally, a study of a random sample of women showed increases in depression and perceived stress.^187^ All of these studies were of fair quality.

### Potential moderators

Table 1 in Supplementary Appendix 5 presents all reported exposures and outcomes, subdivided by potential moderators (geographical region and study size) separately, for each direction of change. The majority of both small and large studies, and studies from all geographical regions, reported increased negative effects on mental health, and thus neither the influence of geographical region differences nor the ‘small-study effect’ were considered to pose any risks for the interpretation of our results.

## Discussion

This systematic review resulted in 174 studies assessing the mental health impacts of the COVID-19 pandemic (87 studies), 2008 economic crisis (84 studies) and SARS epidemic (three studies). Most studies reported effects on affective disorders. Mostly, these studies found increased rates, as might be expected because of increased prevalence of risk factors. For the COVID-19 pandemic, these include uncertainty; loss of income; inactivity; limited access to basic services; increased access to food, alcohol and online gambling; and decreased social support.^188^ However, some populations experienced improvements in affective disorders. These populations included postpartum women, university students, patients from general practice and patients from a sleep clinic. Future studies may delineate the ways in which these populations differed in terms of risk and protective factors, perhaps in part because of the various pandemic responses.

Our findings showed that mental healthcare utilisation as a result of the COVID-19 pandemic did not increase in the same manner as it did in result of the economic crisis; regulations on travel and quarantine may have resulted in mental healthcare visits becoming more difficult and impractical.^189^ Further, we found two studies that showed an increase in severity of mental health problems among those using services during the pandemic, indicating a shift away from seeking mental healthcare for milder problems, with a parallel increase in severity. Retaining existing mental health services, scaling up effective practices and promoting new practices that expand access and provide cost-effective delivery, as well as utilising to peer support and remote health delivery, should be prioritised during the COVID-19 pandemic.^188^ Indeed, previous reports of the mental health effects of the SARS epidemic have illustrated that the negative consequences can even be maintained in the long term,^5^ thus further emphasising the importance of accessible prevention and treatment strategies.

Overall, we found that socioeconomic factors and unemployment resulting from the economic crisis had negative effects. Previous studies have also reported on the deleterious consequences of economic crises on mental health;^5^ that the main risk factors mediating these effects include unemployment, indebtedness, precarious working conditions, inequalities, lack of social connectedness and housing instability;^190^ and that the negative impact of economic hardship on mental health may also continue further in bi-directional manner.^191^ Also, in line with our findings, previous work has suggested that men at working age are at particular risk.^190^ It may thus be expected that these population groups will also be negatively affected by the COVID-19 pandemic and economic downturn.

Contrary to the large number of studies assessing suicide rates in relation to the economic crisis, our review did not find many studies in relation to the COVID-19 pandemic. The few studies we did identify showed either that rates decreased or remained unaltered, in contradiction to studies on the economic crisis. Follow-ups of included studies on the pandemic are short, but in the longer term, an increase in suicide rates as a result of the pandemic might be expected because of the increase in many of the known risk factors for suicide, including social isolation, substance misuse, economic hardship, unemployment and uncertainty.^192^

A limitation of our study was the necessity to narrow the scope of our search strategies to search terms found in titles and abstracts, which was done because of the large number of published studies on the topic. This may have resulted in us missing some relevant studies. Also, we were not able to conduct searches in non-English-language publications or grey literature, which is also a limitation. However, a ‘small-study effect’ is unlikely to be present in our review, as shown in the analysis of study size as a potential moderator. Altogether, this indicates that the risk of publication bias, even if present, could be considered as low. Furthermore, our findings reflect what others have noted: toward the end of 2020, mental health was one of the most common topics for research being conducted on the effects of the COVID-19 pandemic, although the quantity was not matched by quality^193^ – our included studies on the economic crisis were overall of better quality than those on the COVID-19 pandemic. Strengths of our study was its systematic nature and broad scope, which allowed us both to see emerging early evidence and possible longer-term impacts of the COVID-19 pandemic on mental health.

Our findings highlight the importance of making mental health services available, accessible and sustainable for those in need. Also, seeing as the socioeconomically disadvantaged are at increased risk of adverse mental health outcomes, these populations should be particular targets of policy interventions during the COVID-19 pandemic. Moreover, our review covers a broad range of mental health outcomes, both in clinical and general populations, in association with worldwide crises, which provides an invaluable basis for future systematic reviews that are more specific in their topics. Since most studies identified though our review were conducted in high-income countries, it would be invaluable to conduct more studies in low- and middle-income countries. Finally, we expect future research, with longer-term follow-up periods, to be able to elucidate the specific effects of the COVID-19 pandemic on mental health. In addition, international comparisons of mental health outcomes may allow detailed analyses on the differential mental health effects of the pandemic and economic mitigation measures taken by different countries.

## Data Availability

Data availability is not applicable to this article as no new data were created or analysed in this study.

## Supplementary material

For supplementary material accompanying this paper visit http://dx.doi.org/10.1192/bjo.2022.587. click here to view supplementary material
