# Supplementary material for: Effects of the COVID-19 pandemic and previous pandemics, epidemics and economic crises on mental health: systematic review
Source: BJPsych Open. 2022 Oct 10;8(6):e181. doi: 10.1192/bjo.2022.587 (PMC9551492; doi:10.1192/bjo.2022.587)
Supplement: Supplementary file 1 [file S2056472422005877sup001.zip › S2056472422005877sup001.docx]

**Appendix 1:** Search strings for databases searched

**PubMed**

"mental illness"[Title] OR "stress"[Title] OR "depression"[Title] OR "Anxiety"[Title] OR "substance abuse"[Title/Abstract] OR "psychosis"[Title] OR "suicide"[Title] OR "panic disorder"[Title] OR "affective disorder"[Title] OR "eating disorder"[Title/Abstract] OR "burnout"[Title] OR "QOL"[Title] OR "prescription drugs"[Title/Abstract] OR "occupational health"[Title] OR "PTSD"[Title] OR "trauma"[Title] OR "post-covid"[Title] OR "long covid"[Title] OR "well-being"[Title] OR "mental health"[Title] OR "psychiatric disorder"[Title] OR (("emotions"[MeSH Terms] OR "emotions"[All Fields] OR "feeling"[All Fields] OR "feelings"[All Fields] OR "feels"[All Fields]) AND "of insecurity"[Title/Abstract]) OR "sleep disorder"[Title/Abstract] OR "psychosocial disability"[Title] OR "psychological distress"[Title] OR "worry"[Title/Abstract] OR "Mental Disorders"[MeSH Terms:noexp] OR ("Depressive Disorder"[MeSH Major Topic] OR "depression"[MeSH Major Topic]) OR "Depressive Disorder"[MeSH Terms:noexp] OR "Anxiety"[MeSH Terms:noexp] OR "psychotic disorders"[MeSH Terms] OR "suicide"[MeSH Terms] OR "panic disorder"[MeSH Terms] OR "feeding and eating disorders"[MeSH Terms] OR "burnout, professional"[MeSH Terms] OR "quality of life"[MeSH Terms] OR "prescription drugs"[MeSH Terms] OR "occupational health"[MeSH Terms] OR "stress disorders, post traumatic"[MeSH Terms] OR "Psychological Trauma"[MeSH Terms:noexp] OR (("post-acute"[All Fields] AND ("severe acute respiratory syndrome coronavirus 2"[Supplementary Concept] OR "severe acute respiratory syndrome coronavirus 2"[All Fields] OR "ncov"[All Fields] OR "2019 ncov"[All Fields] OR "covid-19"[All Fields] OR "sars cov 2"[All Fields] OR (("coronavirus"[All Fields] OR "cov"[All Fields]) AND 2019/11/01:3000/12/31[Date - Publication]))) AND "syndrome"[MeSH Major Topic]) OR ((("severe acute respiratory syndrome coronavirus 2"[Supplementary Concept] OR "severe acute respiratory syndrome coronavirus 2"[All Fields] OR "ncov"[All Fields] OR "2019 ncov"[All Fields] OR "covid-19"[All Fields] OR "sars cov 2"[All Fields] OR (("coronavirus"[All Fields] OR "cov"[All Fields]) AND 2019/11/01:3000/12/31[Date - Publication])) AND "post-intensive"[All Fields] AND "care"[All Fields]) AND "syndrome"[MeSH Major Topic]) OR "sleep wake disorders"[MeSH Terms] OR "psychological distress"[MeSH Terms] OR "mental health"[MeSH Major Topic]) AND "english"[Language] AND "english"[Language] AND ("pandemic"[Title] OR "epidemic"[Title] OR "economic crisis"[Title] OR "MERS"[Title] OR "SARS"[Title] OR "swine flu"[Title] OR "covid-19"[Title] OR "pandemics"[MeSH Major Topic] OR ("Epidemics"[MeSH Terms] NOT "Opioid Epidemic"[MeSH Terms]) OR "covid-19"[MeSH Major Topic] OR "economic recession"[MeSH Terms] OR "middle east respiratory syndrome coronavirus"[MeSH Terms] OR "sars virus"[MeSH Terms] OR "sars cov 2"[MeSH Terms]) AND ("Health"[Title/Abstract] OR "illness"[Title/Abstract] OR "hospitali*"[Title/Abstract] OR "isolation"[Title/Abstract] OR "loneliness"[Title/Abstract] OR "economic hardship"[Title/Abstract] OR "unemployment"[Title/Abstract] OR "dept"[Title/Abstract] OR "discrimination"[Title/Abstract] OR "Violence"[Title/Abstract] OR "moral distress"[Title/Abstract] OR "trust"[Title/Abstract] OR "teleworking"[Title/Abstract] OR "lockdown"[Title/Abstract] OR "policy measures"[Title/Abstract] OR "travel restrictions"[Title/Abstract] OR "social distancing"[Title/Abstract] OR ("care"[All Fields] AND "about children"[Title/Abstract]) OR "distant education"[Title/Abstract] OR (("limit"[All Fields] OR "limitation"[All Fields] OR "limitations"[All Fields] OR "limited"[All Fields] OR "limiting"[All Fields] OR "limits"[All Fields]) AND "health care capacity"[Title/Abstract]) OR "media"[Title/Abstract] OR "quality of healthcare"[Title/Abstract] OR "Health"[MeSH Terms:noexp] OR "hospitalization"[MeSH Terms] OR "social isolation"[MeSH Terms] OR "loneliness"[MeSH Terms] OR "unemployment"[MeSH Terms] OR "Social Discrimination"[MeSH Terms:noexp] OR "Violence"[MeSH Terms:noexp] OR "trust"[MeSH Terms] OR "teleworking"[MeSH Terms] OR "physical distancing"[MeSH Terms] OR "Communicable Disease Control"[MeSH Terms:noexp] OR "child day care centers"[MeSH Terms] OR "education, distance"[MeSH Terms] OR "Health Resources"[MeSH Terms:noexp] OR "Quality of Health Care"[MeSH Terms:noexp] OR "Communications Media"[MeSH Terms:noexp] OR "Mass Media"[MeSH Terms:noexp]) AND ("english"[Language] AND 2000/01/01:2021/12/31[Date - Publication]) AND "english"[Language]

**Web of Science**

TI=(“pandemic*” OR “epidemic*” OR "economic crisis" OR “SARS” OR “MERS” OR “covid-19” OR "swine flu") AND TS=(“health” OR “illness” OR “hospitali*” OR “isolation” OR “loneliness” OR "economic hardship" OR “unemployment” OR “dept” OR “discrimination” OR “violence” OR "moral distress" OR “trust” OR “teleworking” OR “lockdown” OR "policy measures" OR "travel restrictions" OR "social distancing" OR "care about children" OR "distant education" OR "limited health care capacity" OR “media” OR "quality of healthcare") AND TS=("mental illness" OR “stress” OR “depression” OR “anxiety” OR "substance abuse" OR “psychosis” OR “suicide” OR "panic disorder" OR "affective disorder" OR "eating disorder" OR “burnout” OR QOL OR "prescription drugs" OR "occupational health" OR PTDS OR “trauma” OR "post-covid" OR "long covid" OR “well-being” OR "mental health" OR "psychiatric disorder" OR "feelings of insecurity" OR "sleep disorder" OR "psychosocial disability" OR "psychological distress" OR “worry”)

**PsycInfo**

(pandemic or epidemic or MERS or SARS or swine flu or covid-19).m_titl. AND (health or Illness or hospitalization or isolation or loneliness or economic hardship or unemployment or debt or discrimination or violence or moral distress or trust or teleworking or Lockdown or policy measures or travel restrictions or social distancing or care about children or distant education or limited health care capacity or media or quality of healthcare).mp. [mp=title, abstract, heading word, table of contents, key concepts, original title, tests & measures, mesh] AND (mental illness or stress or depression or anxiety or substance abuse or psychosis or suicide or panic disorder or affective disorder or eating disorder or burnout or QOL or prescription drugs or occupational health or PTSD or trauma or post-covid or long covid or well-being or mental health or psychiatric disorder or feelings of insecurity or sleep disorder or psychosocial disability or psychological distress or worry).mp. [mp=title, abstract, heading word, table of contents, key concepts, original title, tests & measures, mesh]

**Sociological abstracts**

ti(pandemic OR epidemic OR economic crisis OR MERS OR SARS OR “swine flu” OR covid-19 OR MAINSUBJECT.EXACT("Economic Crises") OR MAINSUBJECT.EXACT("Epidemics")) AND ab(“health” OR “Illness” OR “hospitali*” OR “isolation” OR “loneliness” OR “economic hardship” OR “unemployment” OR “debt” OR “discrimination” OR “violence” OR "moral distress" OR “trust” OR “teleworking” OR “Lockdown” OR "policy measures" OR "travel restrictions" OR "social distancing" OR "care about children" OR "distant education" OR "limited health care capacity" OR “media” OR "quality of healthcare") OR MAINSUBJECT.EXACT("Health") OR MAINSUBJECT.EXACT("Illness") OR MAINSUBJECT.EXACT("Social Isolation") OR MAINSUBJECT.EXACT("Loneliness") OR MAINSUBJECT.EXACT("Economic Crises") OR MAINSUBJECT.EXACT("Unemployment") OR MAINSUBJECT.EXACT("Discrimination") OR MAINSUBJECT.EXACT("Trust") OR MAINSUBJECT.EXACT("Policy") OR MAINSUBJECT.EXACT("Social Distance") OR MAINSUBJECT.EXACT("Distance Education") OR MAINSUBJECT.EXACT("Mass Media") AND ab("mental illness" OR “stress” OR “depression” OR “anxiety” OR "substance abuse" OR “psychosis” OR “suicide” OR "panic disorder" OR “affective disorder” OR “eating disorder” OR “burnout” OR QOL OR "prescription drugs" OR "occupational health" OR PTSD OR “trauma” OR "post-covid" OR "long covid" OR "well-being" OR "mental health" OR "psychiatric disorder" OR "feelings of insecurity" OR "sleep disorder" OR "psychosocial disability" OR "psychological distress" OR “worry”) OR MAINSUBJECT.EXACT("Mental Illness") OR MAINSUBJECT.EXACT("Mental Health") OR MAINSUBJECT.EXACT("Depression (Psychology)") OR MAINSUBJECT.EXACT("Substance Abuse") OR MAINSUBJECT.EXACT("Suicide") OR MAINSUBJECT.EXACT("Affective Illness") OR MAINSUBJECT.EXACT("Eating Disorders") OR MAINSUBJECT.EXACT("Quality of Life") OR (MAINSUBJECT.EXACT("Posttraumatic Stress Disorder") OR MAINSUBJECT.EXACT("Trauma")) OR MAINSUBJECT.EXACT("Security")
