# Supplementary material for: Effects of the COVID-19 pandemic and previous pandemics, epidemics and economic crises on mental health: systematic review
Source: BJPsych Open. 2022 Oct 10;8(6):e181. doi: 10.1192/bjo.2022.587 (PMC9551492; doi:10.1192/bjo.2022.587)
Supplement: Supplementary file 1 [file S2056472422005877sup001.zip › S2056472422005877sup002.docx]

**Appendix 2: Quality assessment of included studies according to the Newcastle Ottawa Scale:**

| Ref | Exposure | Representativeness of the cohort | Selection of the non-exposed cohort | Ascertainment of exposure | Demonstration that outcome of interest was not present at start of study | Comparability of cohorts on basis of design or analysis | Assessment of outcome | Was follow-up long enough for outcomes to occur | Adequacy of follow-up cohorts | Total quality |
| --- | --- | --- | --- | --- | --- | --- | --- | --- | --- | --- |
| 14 | Covid-19 | --- | * | * | * | * | --- | * | * | 6 |
| 15 | Covid-19 | --- | * | * | * | * | --- | * | * | 6 |
| 16 | Covid-19 | --- | * | * | * | * | --- | * | --- | 5 |
| 17 | Covid-19 | * | * | * | * | * | --- | * | --- | 6 |
| 18 | Covid-19 | * | --- | * | * | * | --- | * | * | 6 |
| 19 | Covid-19 | --- | * | * | * | * | --- | * | --- | 5 |
| 20 | Covid-19 | --- | * | * | * | * | --- | * | --- | 5 |
| 21 | Covid-19 | * | * | * | * | * | --- | * | * | 7 |
| 22 | Covid-19 | * | * | * | * | * | --- | * | --- | 6 |
| 23 | Covid-19 | --- | * | * | * | * | --- | * | * | 6 |
| 24 | Covid-19 | --- | * | * | * | * | --- | * | --- | 5 |
| 25 | Covid-19 | --- | * | * | * | * | --- | * | --- | 5 |
| 26 | Covid-19 | --- | * | * | * | * | --- | * | --- | 5 |
| 27 | Covid-19 | * | * | * | * | * | --- | * | * | 7 |
| 28 | Covid-19 | --- | * | * | * | * | --- | * | --- | 5 |
| 29 | Covid-19 | * | * | * | * | * | --- | * | * | 7 |
| 30 | Covid-19 | --- | * | * | * | * | --- | * | --- | 5 |
| 31 | Covid-19 | * | * | * | * | * | * | * | --- | 7 |
| 32 | Covid-19 | --- | * | * | * | * | --- | * | --- | 5 |
| 33 | Covid-19 | * | * | * | * | * | --- | * | --- | 6 |
| 34 | Covid-19 | --- | * | * | * | * | --- | * | * | 6 |
| 35 | Covid-19 | --- | * | * | * | * | --- | * | * | 6 |
| 36 | Covid-19 | --- | * | * | * | * | --- | * | * | 6 |
| 37 | Covid-19 | * | * | * | * | * | --- | * | --- | 6 |
| 38 | Covid-19 | --- | * | * | * | * | --- | * | --- | 5 |
| 39 | Covid-19 | --- | * | * | * | * | --- | * | * | 6 |
| 40 | Covid-19 | * | * | * | * | * | --- | * | --- | 6 |
| 41 | Covid-19 | --- | * | * | * | * | --- | * | * | 6 |
| 42 | Covid-19 | --- | * | * | * | * | --- | * | * | 6 |
| 43 | Covid-19 | * | * | * | * | * | --- | * | --- | 6 |
| 44 | Covid-19 | --- | * | * | * | * | --- | * | --- | 5 |
| 47 | Covid-19 | --- | * | * | * | * | --- | * | --- | 5 |
| 48 | Covid-19 | --- | * | * | * | * | --- | * | --- | 5 |
| 49 | Covid-19 | --- | --- | --- | * | * | --- | * | --- | 3 |
| 50 | Covid-19 | * | * | * | * | * | --- | * | --- | 6 |
| 51 | Covid-19 | --- | * | * | * | * | --- | * | * | 6 |
| 45 | Covid-19 | --- | * | * | * | * | --- | * | --- | 5 |
| 46 | Covid-19 | --- | * | * | * | * | --- | * | * | 6 |
| 52 | Covid-19 | * | * | * | * | * | --- | * | * | 7 |
| 53 | Covid-19 | --- | * | * | * | * | --- | * | --- | 5 |
| 54 | Covid-19 | --- | * | * | * | * | --- | * | * | 6 |
| 55 | Covid-19 | --- | * | * | * | * | --- | * | * | 6 |
| 56 | Covid-19 | * | * | * | * | * | * | * | --- | 7 |
| 57 | Covid-19 | * | * | * | * | * | * | * | --- | 7 |
| 58 | Covid-19 | * | * | * | * | * | * | * | * | 8 |
| 59 | Covid-19 | * | * | * | * | * | * | * | --- | 7 |
| 60 | Covid-19 | * | * | * | * | * | * | * | * | 8 |
| 61 | Covid-19 | --- | * | * | * | * | --- | * | --- | 5 |
| 62 | Covid-19 | * | * | * | * | * | --- | * | * | 7 |
| 63 | Covid-19 | * | * | * | * | * | --- | * | --- | 6 |
| 64 | Covid-19 | * | * | * | * | --- | --- | * | --- | 5 |
| 65 | Covid-19 | * | * | * | * | * | --- | * | * | 7 |
| 66 | Covid-19 | * | * | * | * | * | --- | * | --- | 6 |
| 67 | Covid-19 | * | * | * | * | * | --- | * | * | 7 |
| 68 | Covid-19 | * | * | * | * | --- | --- | * | --- | 5 |
| 69 | Covid-19 | * | * | * | * | * | --- | * | * | 7 |
| 70 | Covid-19 | --- | --- | * | * | --- | --- | * | --- | 3 |
| 71 | Covid-19 | --- | * | * | * | * | --- | * | --- | 5 |
| 72 | Covid-19 | * | --- | * | * | * | --- | * | --- | 5 |
| 73 | Covid-19 | * | * | * | * | * | --- | * | --- | 6 |
| 74 | Covid-19 | --- | * | * | * | * | --- | * | --- | 5 |
| 75 | Covid-19 | --- | * | * | * | * | --- | * | * | 6 |
| 76 | Covid-19 | --- | * | * | * | * | --- | * | --- | 5 |
| 77 | Covid-19 | * | * | * | * | * | * | * | * | 8 |
| 78 | Covid-19 | --- | * | * | * | * | --- | * | * | 6 |
| 79 | Covid-19 | --- | * | * | * | * | --- | * | --- | 5 |
| 80 | Covid-19 | --- | * | * | * | * | --- | * | * | 6 |
| 81 | Covid-19 | --- | * | * | * | * | --- | * | * | 6 |
| 82 | Covid-19 | --- | * | * | * | * | --- | * | --- | 5 |
| 83 | Covid-19 | * | * | * | * | * | --- | * | * | 7 |
| 84 | Covid-19 | --- | * | * | * | * | --- | * | * | 6 |
| 85 | Covid-19 | --- | * | * | * | * | --- | * | --- | 5 |
| 86 | Covid-19 | --- | * | * | * | * | --- | * | --- | 5 |
| 87 | Covid-19 | --- | * | * | * | * | --- | * | --- | 5 |
| 88 | Covid-19 | --- | * | * | * | * | --- | * | --- | 5 |
| 89 | Covid-19 | * | * | * | * | * | --- | * | * | 7 |
| 90 | Covid-19 | --- | * | * | * | * | --- | * | * | 6 |
| 91 | Covid-19 | * | * | * | * | * | * | * | --- | 7 |
| 92 | Covid-19 | * | * | * | * | * | * | * | --- | 7 |
| 93 | Covid-19 | * | * | * | * | * | * | * | --- | 7 |
| 94 | Covid-19 | * | * | * | * | * | * | * | --- | 7 |
| 95 | Covid-19 | * | * | * | * | * | * | * | --- | 7 |
| 96 | Covid-19 | * | * | * | * | * | * | * | * | 8 |
| 97 | Covid-19 | * | * | * | * | * | * | * | --- | 7 |
| 98 | Covid-19 | * | * | * | * | * | * | * | --- | 7 |
| 99 | Covid-19 | --- | * | * | * | * | --- | * | --- | 5 |
| 100 | Covid-19 | * | * | * | * | * | * | * | * | 8 |
| 101 | Econ crisis | * | * | * | * | * | --- | * | --- | 6 |
| 102 | Econ crisis | * | * | * | * | * | --- | * | --- | 6 |
| 103 | Econ crisis | * | * | * | * | * | --- | * | * | 7 |
| 104 | Econ crisis | * | * | * | * | * | --- | * | * | 7 |
| 105 | Econ crisis | * | * | * | * | * | --- | * | --- | 6 |
| 106 | Econ crisis | * | * | * | * | * | --- | * | --- | 6 |
| 107 | Econ crisis | * | * | * | * | * | --- | * | --- | 6 |
| 108 | Econ crisis | * | * | * | * | * | --- | * | --- | 6 |
| 109 | Econ crisis | * | * | * | * | * | --- | * | --- | 6 |
| 110 | Econ crisis | --- | * | * | * | * | --- | * | --- | 5 |
| 111 | Econ crisis | * | * | * | * | * | --- | * | --- | 6 |
| 112 | Econ crisis | * | * | * | * | * | --- | * | --- | 6 |
| 113 | Econ crisis | * | * | * | * | * | --- | * | --- | 6 |
| 114 | Econ crisis | * | * | * | * | * | --- | * | * | 7 |
| 115 | Econ crisis | * | * | * | * | * | --- | * | --- | 6 |
| 116 | Econ crisis | * | * | * | * | * | * | * | --- | 7 |
| 117 | Econ crisis | * | * | * | * | * | * | * | --- | 7 |
| 118 | Econ crisis | * | * | * | * | * | * | * | --- | 7 |
| 119 | Econ crisis | * | * | * | * | * | * | * | --- | 7 |
| 120 | Econ crisis | * | * | * | * | * | * | * | --- | 7 |
| 121 | Econ crisis | * | * | * | * | * | * | * | --- | 7 |
| 122 | Econ crisis | * | * | * | * | * | * | * | --- | 7 |
| 123 | Econ crisis | * | * | * | * | * | * | * | --- | 7 |
| 124 | Econ crisis | * | * | * | * | * | * | * | --- | 7 |
| 125 | Econ crisis | * | * | * | * | * | * | * | --- | 7 |
| 126 | Econ crisis | * | * | * | * | * | * | * | --- | 7 |
| 127 | Econ crisis | * | * | * | * | * | * | * | --- | 7 |
| 128 | Econ crisis | * | * | * | * | * | * | * | --- | 7 |
| 129 | Econ crisis | * | * | * | * | * | * | * | --- | 7 |
| 130 | Econ crisis | * | * | * | * | * | * | * | --- | 7 |
| 131 | Econ crisis | * | * | * | * | * | * | * | --- | 7 |
| 132 | Econ crisis | * | * | * | * | * | * | * | --- | 7 |
| 133 | Econ crisis | * | * | * | * | * | * | * | --- | 7 |
| 134 | Econ crisis | * | * | * | * | * | * | * | --- | 7 |
| 135 | Econ crisis | * | * | * | * | * | * | * | --- | 7 |
| 136 | Econ crisis | * | * | * | * | * | * | * | --- | 7 |
| 137 | Econ crisis | * | * | * | * | * | * | * | --- | 7 |
| 138 | Econ crisis | * | * | * | * | * | * | * | --- | 7 |
| 139 | Econ crisis | * | * | * | * | * | * | * | --- | 7 |
| 140 | Econ crisis | * | * | * | * | * | * | * | --- | 7 |
| 141 | Econ crisis | * | * | * | * | * | * | * | --- | 7 |
| 142 | Econ crisis | * | * | * | * | * | * | * | --- | 7 |
| 143 | Econ crisis | * | * | * | * | * | * | * | --- | 7 |
| 144 | Econ crisis | * | * | * | * | * | * | * | --- | 7 |
| 145 | Econ crisis | * | * | * | * | * | * | * | --- | 7 |
| 146 | Econ crisis | * | * | * | * | * | * | * | --- | 7 |
| 147 | Econ crisis | * | * | * | * | * | * | * | --- | 7 |
| 148 | Econ crisis | * | * | * | * | * | * | * | --- | 7 |
| 149 | Econ crisis | * | * | * | * | * | * | * | --- | 7 |
| 150 | Econ crisis | * | * | * | * | * | * | * | --- | 7 |
| 151 | Econ crisis | * | * | * | * | * | * | * | --- | 7 |
| 152 | Econ crisis | * | * | * | * | * | * | * | --- | 7 |
| 153 | Econ crisis | * | * | * | * | * | --- | * | --- | 6 |
| 154 | Econ crisis | * | * | * | * | * | --- | * | * | 7 |
| 155 | Econ crisis | * | * | * | * | * | --- | * | --- | 6 |
| 156 | Econ crisis | * | * | * | * | * | --- | * | * | 7 |
| 157 | Econ crisis | * | * | * | * | * | --- | * | --- | 6 |
| 158 | Econ crisis | * | * | * | * | * | --- | * | --- | 6 |
| 159 | Econ crisis | * | * | * | * | * | --- | * | --- | 6 |
| 160 | Econ crisis | * | * | * | * | * | --- | * | --- | 6 |
| 161 | Econ crisis | * | * | * | * | * | --- | * | --- | 6 |
| 162 | Econ crisis | * | * | * | * | * | --- | * | * | 7 |
| 163 | Econ crisis | --- | * | * | * | * | --- | * | --- | 5 |
| 164 | Econ crisis | * | * | * | * | * | --- | * | --- | 6 |
| 165 | Econ crisis | --- | * | * | * | * | --- | * | --- | 5 |
| 166 | Econ crisis | * | * | * | * | * | --- | * | * | 7 |
| 167 | Econ crisis | * | * | * | * | * | --- | * | --- | 6 |
| 168 | Econ crisis | * | * | * | * | * | --- | * | * | 7 |
| 169 | Econ crisis | * | * | * | * | * | --- | * | --- | 6 |
| 170 | Econ crisis | * | * | * | * | * | --- | * | * | 7 |
| 171 | Econ crisis | * | * | * | * | * | --- | * | * | 7 |
| 172 | Econ crisis | * | * | * | * | * | * | * | --- | 7 |
| 173 | Econ crisis | * | * | * | * | * | --- | * | --- | 6 |
| 174 | Econ crisis | * | * | * | * | * | --- | * | * | 7 |
| 175 | Econ crisis | * | --- | * | * | --- | --- | * | --- | 4 |
| 176 | Econ crisis | * | * | * | * | * | --- | * | --- | 6 |
| 177 | Econ crisis | * | * | * | * | --- | --- | * | --- | 5 |
| 178 | Econ crisis | * | * | * | * | * | * | * | * | 8 |
| 179 | Econ crisis | * | * | * | * | * | * | * | --- | 7 |
| 180 | Econ crisis | * | * | * | * | * | --- | * | --- | 6 |
| 181 | Econ crisis | * | * | * | * | * | * | * | --- | 7 |
| 182 | Econ crisis | * | * | * | * | * | * | * | --- | 7 |
| 183 | Econ crisis | * | * | * | * | * | * | * | --- | 7 |
| 184 | Econ crisis | * | * | * | * | * | * | * | --- | 7 |
| 185 | SARS | * | * | * | * | * | --- | * | --- | 6 |
| 186 | SARS | * | * | * | * | * | * | * | --- | 7 |
| 187 | SARS | --- | * | * | * | * | --- | --- | * | 5 |
