# Supplementary material for: Effects of the COVID-19 pandemic and previous pandemics, epidemics and economic crises on mental health: systematic review
Source: BJPsych Open. 2022 Oct 10;8(6):e181. doi: 10.1192/bjo.2022.587 (PMC9551492; doi:10.1192/bjo.2022.587)
Supplement: Supplementary file 1 [file S2056472422005877sup001.zip › S2056472422005877sup003.docx]

**Appendix 3:** Articles excluded via full-text screening:

| **Article** | **Reason for Exclusion**  **PICOS** |
| --- | --- |
| Abawi O, Welling MS, van den Eynde E, van Rossum EFC, Halberstadt J, van den Akker ELT, van der Voorn B. COVID-19 related anxiety in children and adolescents with severe obesity: A mixed-methods study. Clin Obes. 2020 Dec;10(6):e12412. doi: 10.1111/cob.12412. Epub 2020 Sep 13. PMID: 32920993; PMCID: PMC7685119. | C |
| Adadms EL, Smith D, Caccavale LJ, Bean MK. Parents are stressed! Patterns of parent stress across COVID-19. Res Sq [Preprint]. 2020 Dec 9:rs.3.rs-66730. doi: 10.21203/rs.3.rs-66730/v2. Update in: Front Psychiatry. 2021 Apr 08;12:626456. PMID: 33330859; PMCID: PMC7743082. | C |
| Aebischer O, Weilenmann S, Gachoud D, Méan M, Spiller TR. Physical and psychological health of medical students involved in the coronavirus disease 2019 response in Switzerland. Swiss Med Wkly. 2020 Dec 11;150:w20418. doi: 10.4414/smw.2020.20418. PMID: 33306812. | C |
| Agarwal A, Agrawal S, Agarwal A. Mental health among patients in a COVID-19-dedicated facility. Trans R Soc Trop Med Hyg. 2021 Jan 7;115(1):1-2. doi: 10.1093/trstmh/traa095. PMID: 32986096; PMCID: PMC7543627. | P |
| Agha S. Mental well-being and association of the four factors coping structure model: A perspective of people living in lockdown during COVID-19. Ethics Med Public Health. 2021 Mar;16:100605. doi: 10.1016/j.jemep.2020.100605. Epub 2020 Oct 14. PMID: 33078131; PMCID: PMC7556853. | C |
| Agoramoorthy G. The coronavirus stress: A reality check of India's mental health social agenda. Int J Soc Psychiatry. 2020 Sep;66(6):623-624. doi: 10.1177/0020764020925498. Epub 2020 May 21. PMID: 32438840. | C |
| Agudelo-Suárez AA, Ronda E, Vázquez-Navarrete ML, García AM, Martínez JM, Benavides FG. Impact of economic crisis on mental health of migrant workers: what happened with migrants who came to Spain to work? Int J Public Health. 2013 Aug;58(4):627-31. doi: 10.1007/s00038-013-0475-0. Epub 2013 May 11. PMID: 23665911. | C |
| Aguilar-Palacio I, Carrera-Lasfuentes P, Rabanaque MJ. Youth unemployment and economic recession in Spain: influence on health and lifestyles in young people (16-24 years old). Int J Public Health. 2015 May;60(4):427-35. doi: 10.1007/s00038-015-0668-9. Epub 2015 Feb 28. PMID: 25724155. | S |
| Ahmed F, Zhao F, Faraz NA. How and When Does Inclusive Leadership Curb Psychological Distress During a Crisis? Evidence From the COVID-19 Outbreak. Front Psychol. 2020 Aug 6;11:1898. doi: 10.3389/fpsyg.2020.01898. PMID: 32849111; PMCID: PMC7423991. | S |
| Ahmed H, Patel K, Greenwood DC, Halpin S, Lewthwaite P, Salawu A, Eyre L, Breen A, O'Connor R, Jones A, Sivan M. Long-term clinical outcomes in survivors of severe acute respiratory syndrome and Middle East respiratory syndrome coronavirus outbreaks after hospitalisation or ICU admission: A systematic review and meta-analysis. J Rehabil Med. 2020 May 31;52(5):jrm00063. doi: 10.2340/16501977-2694. PMID: 32449782. | C |
| Ahmed MZ, Ahmed O, Aibao Z, Hanbin S, Siyu L, Ahmad A. Epidemic of COVID-19 in China and associated Psychological Problems. Asian J Psychiatr. 2020 Jun;51:102092. doi: 10.1016/j.ajp.2020.102092. Epub 2020 Apr 14. PMID: 32315963; PMCID: PMC7194662. | S |
| Ahmed S, Khaium MO, Tazmeem F. COVID-19 lockdown in India triggers a rapid rise in suicides due to the alcohol withdrawal symptoms: Evidence from media reports. Int J Soc Psychiatry. 2020 Dec;66(8):827-829. doi: 10.1177/0020764020938809. Epub 2020 Jun 26. PMID: 32586209. | C |
| Ahmed ZM, Khalil MF, Kohail AM, Eldesouky IF, Elkady A, Shuaib A. The Prevalence and Predictors of Post-Stroke Depression and Anxiety During COVID-19 Pandemic. J Stroke Cerebrovasc Dis. 2020 Dec;29(12):105315. doi: 10.1016/j.jstrokecerebrovasdis.2020.105315. Epub 2020 Sep 10. PMID: 32958396; PMCID: PMC7834239. | C |
| Ahorsu DK, Imani V, Lin CY, Timpka T, Broström A, Updegraff JA, Årestedt K, Griffiths MD, Pakpour AH. Associations Between Fear of COVID-19, Mental Health, and Preventive Behaviours Across Pregnant Women and Husbands: An Actor-Partner Interdependence Modelling. Int J Ment Health Addict. 2020 Jun 11:1-15. doi: 10.1007/s11469-020-00340-x. Epub ahead of print. PMID: 32837427; PMCID: PMC7289236. | C |
| Akkaya-Kalayci T, Kothgassner OD, Wenzel T, Goreis A, Chen A, Ceri V, Özlü-Erkilic Z. The Impact of the COVID-19 Pandemic on Mental Health and Psychological Well-Being of Young People Living in Austria and Turkey: A Multicenter Study. Int J Environ Res Public Health. 2020 Dec 6;17(23):9111. doi: 10.3390/ijerph17239111. PMID: 33291276; PMCID: PMC7730981. | C |
| Aksoy YE, Koçak V. Psychological effects of nurses and midwives due to COVID-19 outbreak: The case of Turkey. Arch Psychiatr Nurs. 2020 Oct;34(5):427-433. doi: 10.1016/j.apnu.2020.07.011. Epub 2020 Jul 8. PMID: 33032769; PMCID: PMC7341051. | C |
| Alambo A, Padhee S, Banerjee T, Thirunarayan K. COVID-19 and Mental Health/Substance Use Disorders on Reddit: A Longitudinal Study. ArXiv [Preprint]. 2020 Nov 20:arXiv:2011.10518v1. PMID: 33235895; PMCID: PMC7685336. | S |
| Alan H, Eskin Bacaksiz F, Tiryaki Sen H, Taskiran Eskici G, Gumus E, Harmanci Seren AK. "I'm a hero, but…": An evaluation of depression, anxiety, and stress levels of frontline healthcare professionals during COVID-19 pandemic in Turkey. Perspect Psychiatr Care. 2020 Nov 10. doi: 10.1111/ppc.12666. Epub ahead of print. PMID: 33169851. | S |
| Alan S, Gokyildiz Surucu S, Avcibay Vurgec B, Cevik A. An investigation of individuals' health anxiety during the COVID-19 pandemic within the framework of the functional health patterns. Perspect Psychiatr Care. 2020 Oct 27. doi: 10.1111/ppc.12663. Epub ahead of print. PMID: 33111369. | C |
| Al-Dwaikat TN, Aldalaykeh M, Ta'an W, Rababa M. The relationship between social networking sites usage and psychological distress among undergraduate students during COVID-19 lockdown. Heliyon. 2020 Dec 9;6(12):e05695. doi: 10.1016/j.heliyon.2020.e05695. PMID: 33344792; PMCID: PMC7736712. | C |
| Alemany-Arrebola I, Rojas-Ruiz G, Granda-Vera J, Mingorance-Estrada ÁC. Influence of COVID-19 on the Perception of Academic Self-Efficacy, State Anxiety, and Trait Anxiety in College Students. Front Psychol. 2020 Oct 9;11:570017. doi: 10.3389/fpsyg.2020.570017. PMID: 33154727; PMCID: PMC7586314. | S |
| Al-Hashel JY, Ismail II. Impact of coronavirus disease 2019 (COVID-19) pandemic on patients with migraine: a web-based survey study. J Headache Pain. 2020 Sep 24;21(1):115. doi: 10.1186/s10194-020-01183-6. PMID: 32972360; PMCID: PMC7513457. | S |
| Allan SM, Bealey R, Birch J, Cushing T, Parke S, Sergi G, Bloomfield M, Meiser-Stedman R. The prevalence of common and stress-related mental health disorders in healthcare workers based in pandemic-affected hospitals: a rapid systematic review and meta-analysis. Eur J Psychotraumatol. 2020 Oct 16;11(1):1810903. doi: 10.1080/20008198.2020.1810903. PMID: 33244359; PMCID: PMC7678680. | C |
| Al-Mahadin S. Laughing it off: Coronavirus superspreaders, anxiety, and fear in Jordan and Australia. Psychol Trauma. 2020 Aug;12(S1):S45-S46. doi: 10.1037/tra0000630. Epub 2020 Jun 29. PMID: 32597676. | S |
| Almazan AN, Chun AS, Perez-Urbano I. The Medical Student Response to the Mental Health Consequences of COVID-19. Acad Psychiatry. 2020 Dec;44(6):689-690. doi: 10.1007/s40596-020-01313-x. Epub 2020 Sep 21. PMID: 32955711; PMCID: PMC7505041. | C |
| Al-Musharaf S. Prevalence and Predictors of Emotional Eating among Healthy Young Saudi Women during the COVID-19 Pandemic. Nutrients. 2020 Sep 24;12(10):2923. doi: 10.3390/nu12102923. PMID: 32987773; PMCID: PMC7598723. | C |
| Al-Sofiani ME, Albunyan S, Alguwaihes AM, Kalyani RR, Golden SH, Alfadda A. Determinants of mental health outcomes among people with and without diabetes during the COVID-19 outbreak in the Arab Gulf Region. J Diabetes. 2021 Apr;13(4):339-352. doi: 10.1111/1753-0407.13149. Epub 2021 Jan 17. PMID: 33351239. | C |
| Arroyo-Borrell E, Renart G, Saurina C, Saez M. Influence maternal background has on children's mental health. Int J Equity Health. 2017 Apr 18;16(1):63. doi: 10.1186/s12939-017-0559-1. PMID: 28420394; PMCID: PMC5395783. | S |
| Ayaz R, Hocaoğlu M, Günay T, Yardımcı OD, Turgut A, Karateke A. Anxiety and depression symptoms in the same pregnant women before and during the COVID-19 pandemic. J Perinat Med. 2020 Nov 26;48(9):965-970. doi: 10.1515/jpm-2020-0380. PMID: 32887191. | C |
| Aytac, Isik A. ;Rankin, Bruce H. ;Ibikoglu, Arda. The social impact of the 2008 global economic crisis on neighborhoods, households, and individuals in Turkey. Social Indicators Research. 2015. doi http://dx.doi.org/10.1007/s11205-014-0769-5 | C |
| Banerjee D. The impact of Covid-19 pandemic on elderly mental health. Int J Geriatr Psychiatry. 2020 Dec;35(12):1466-1467. doi: 10.1002/gps.5320. Epub 2020 Jun 27. PMID: 32364283; PMCID: PMC7267435. | C |
| Barrea L, Pugliese G, Framondi L, Di Matteo R, Laudisio D, Savastano S, Colao A, Muscogiuri G. Does Sars-Cov-2 threaten our dreams? Effect of quarantine on sleep quality and body mass index. J Transl Med. 2020 Aug 18;18(1):318. doi: 10.1186/s12967-020-02465-y. PMID: 32811530; PMCID: PMC7432549. | C |
| Bendau A, Plag J, Kunas S, Wyka S, Ströhle A, Petzold MB. Longitudinal changes in anxiety and psychological distress, and associated risk and protective factors during the first three months of the COVID-19 pandemic in Germany. Brain Behav. 2021 Feb;11(2):e01964. doi: 10.1002/brb3.1964. Epub 2020 Nov 23. PMID: 33230969; PMCID: PMC7744907. | C |
| Benfante A, Di Tella M, Romeo A, Castelli L. Traumatic Stress in Healthcare Workers During COVID-19 Pandemic: A Review of the Immediate Impact. Front Psychol. 2020 Oct 23;11:569935. doi: 10.3389/fpsyg.2020.569935. PMID: 33192854; PMCID: PMC7645025. | C |
| Berthelot N, Lemieux R, Garon-Bissonnette J, Drouin-Maziade C, Martel É, Maziade M. Uptrend in distress and psychiatric symptomatology in pregnant women during the coronavirus disease 2019 pandemic. Acta Obstet Gynecol Scand. 2020 Jul;99(7):848-855. doi: 10.1111/aogs.13925. Epub 2020 Jun 3. PMID: 32449178. | P |
| Bhattacharjee B, Acharya T. "The COVID-19 Pandemic and its Effect on Mental Health in USA - A Review with Some Coping Strategies". Psychiatr Q. 2020 Dec;91(4):1135-1145. doi: 10.1007/s11126-020-09836-0. PMID: 32829449; PMCID: PMC7443176. | C |
| Blomqvist S, Burström B, Backhans MC. Increasing health inequalities between women in and out of work--the impact of recession or policy change? A repeated cross-sectional study in Stockholm county, 2006 and 2010. Int J Equity Health. 2014 Jul 25;13:51. doi: 10.1186/1475-9276-13-51. PMID: 25063363; PMCID: PMC4126349. | E/I |
| Brady SM, Fenton SAM, Metsios GS, Bosworth A, Duda JL, Kitas GD, Veldhuijzen van Zanten JJCS. Different types of physical activity are positively associated with indicators of mental health and psychological wellbeing in rheumatoid arthritis during COVID-19. Rheumatol Int. 2021 Feb;41(2):335-344. doi: 10.1007/s00296-020-04751-w. Epub 2020 Nov 30. PMID: 33258004; PMCID: PMC7703721. | S |
| Breuer C. Unemployment and Suicide Mortality: Evidence from Regional Panel Data in Europe. Health Econ. 2015 Aug;24(8):936-50. doi: 10.1002/hec.3073. Epub 2014 Jun 17. PMID: 24934277. | E/I |
| Bruckner T, Kim Y, Snowden L. Racial/ethnic disparities in children's emergency mental health after economic downturns. Adm Policy Ment Health. 2014 May;41(3):334-42. doi: 10.1007/s10488-013-0474-8. PMID: 23397232. | E/I |
| Buffel V, Van de Velde S, Bracke P. The mental health consequences of the economic crisis in Europe among the employed, the unemployed, and the non-employed. Soc Sci Res. 2015 Nov;54:263-88. doi: 10.1016/j.ssresearch.2015.08.003. Epub 2015 Aug 28. PMID: 26463548. | C |
| Buss PM, Hartz ZMA, Pinto LF, Rocha CMF. Health promotion and quality of life: a historical perspective of the last two 40 years (1980-2020). Cien Saude Colet. 2020 Dec;25(12):4723-4735. Portuguese, English. doi: 10.1590/1413-812320202512.15902020. Epub 2020 May 23. PMID: 33295496. | S |
| Cai Q, Feng H, Huang J, Wang M, Wang Q, Lu X, Xie Y, Wang X, Liu Z, Hou B, Ouyang K, Pan J, Li Q, Fu B, Deng Y, Liu Y. The mental health of frontline and non-frontline medical workers during the coronavirus disease 2019 (COVID-19) outbreak in China: A case-control study. J Affect Disord. 2020 Oct 1;275:210-215. doi: 10.1016/j.jad.2020.06.031. Epub 2020 Jul 2. PMID: 32734910; PMCID: PMC7329671. | C |
| Calderon-Anyosa RJC, Kaufman JS. Impact of COVID-19 lockdown policy on homicide, suicide, and motor vehicle deaths in Peru. Prev Med. 2021 Feb;143:106331. doi: 10.1016/j.ypmed.2020.106331. Epub 2020 Nov 21. PMID: 33232687; PMCID: PMC7680039. | S |
| Carr MJ, Steeg S, Webb RT, Kapur N, Chew-Graham CA, Abel KM, Hope H, Pierce M, Ashcroft DM. Effects of the COVID-19 pandemic on primary care-recorded mental illness and self-harm episodes in the UK: a population-based cohort study. Lancet Public Health. 2021 Feb;6(2):e124-e135. doi: 10.1016/S2468-2667(20)30288-7. Epub 2021 Jan 11. PMID: 33444560; PMCID: PMC7843955. | S |
| Chang SS, Stuckler D, Yip P, Gunnell D. Impact of 2008 global economic crisis on suicide: time trend study in 54 countries. BMJ. 2013 Sep 17;347:f5239. doi: 10.1136/bmj.f5239. PMID: 24046155; PMCID: PMC3776046. | S |
| Chapman JJ, Hielscher E, Patterson S, Reavley N, Brown WJ, Wyder M, Childs S, Russell A, Suetani S, Scott JG. Preferences of people with mental illness for engaging in exercise programs under COVID-19 restrictions. Australas Psychiatry. 2021 Apr;29(2):175-179. doi: 10.1177/1039856220975299. Epub 2020 Dec 30. PMID: 33380159; PMCID: PMC7780068. | S |
| Chen H, Wang B, Cheng Y, Muhammad B, Li S, Miao Z, Wan B, Abdul M, Zhao Z, Geng D, Xu X. Prevalence of posttraumatic stress symptoms in health care workers after exposure to patients with COVID-19. Neurobiol Stress. 2020 Oct 17;13:100261. doi: 10.1016/j.ynstr.2020.100261. PMID: 33163588; PMCID: PMC7607510. | C |
| Chen H, Zhang K. Insight into the psychological problems on the epidemic of COVID-19 in China by online searching behaviors. J Affect Disord. 2020 Nov 1;276:1093-1094. doi: 10.1016/j.jad.2020.07.128. Epub 2020 Aug 1. PMID: 32771861; PMCID: PMC7395660. | S |
| Chen R, Chou KR, Huang YJ, Wang TS, Liu SY, Ho LY. Effects of a SARS prevention programme in Taiwan on nursing staff's anxiety, depression and sleep quality: a longitudinal survey. Int J Nurs Stud. 2006 Feb;43(2):215-25. doi: 10.1016/j.ijnurstu.2005.03.006. Epub 2005 May 31. PMID: 15927185; PMCID: PMC7094227. | E/I |
| Chen S, Cheng Z, Wu J. Risk factors for adolescents' mental health during the COVID-19 pandemic: a comparison between Wuhan and other urban areas in China. Global Health. 2020 Oct 9;16(1):96. doi: 10.1186/s12992-020-00627-7. PMID: 33036622; PMCID: PMC7545801. | C |
| Chen S, Jones PB, Underwood BR, Moore A, Bullmore ET, Banerjee S, Osimo EF, Deakin JB, Hatfield CF, Thompson FJ, Artingstall JD, Slann MP, Lewis JR, Cardinal RN. The early impact of COVID-19 on mental health and community physical health services and their patients' mortality in Cambridgeshire and Peterborough, UK. J Psychiatr Res. 2020 Dec;131:244-254. doi: 10.1016/j.jpsychires.2020.09.020. Epub 2020 Sep 22. PMID: 33035957; PMCID: PMC7508053. | C |
| Chen YY, Yip PS, Lee C, Fan HF, Fu KW. Economic fluctuations and suicide: a comparison of Taiwan and Hong Kong. Soc Sci Med. 2010 Dec;71(12):2083-90. doi: 10.1016/j.socscimed.2010.09.043. Epub 2010 Oct 21. PMID: 21071128. | E/I |
| Cheung CK, Tse JW. Institutional trust as a determinant of anxiety during the SARS crisis in Hong Kong. Soc Work Public Health. 2008;23(5):41-54. doi: 10.1080/19371910802053224. PMID: 19301537. | C |
| Chew QH, Chia FL, Ng WK, Lee WCI, Tan PLL, Wong CS, Puah SH, Shelat VG, Seah ED, Huey CWT, Phua EJ, Sim K. Perceived Stress, Stigma, Traumatic Stress Levels and Coping Responses amongst Residents in Training across Multiple Specialties during COVID-19 Pandemic-A Longitudinal Study. Int J Environ Res Public Health. 2020 Sep 9;17(18):6572. doi: 10.3390/ijerph17186572. PMID: 32916996; PMCID: PMC7559162. | C |
| Chieffo DPR, Delle Donne V, Massaroni V, Mastrilli L, Belella D, Monti L, Silveri MC, Cauda R. Psychopathological profile in COVID-19 patients including healthcare workers: the implications. Eur Rev Med Pharmacol Sci. 2020 Nov;24(22):11964-11970. doi: 10.26355/eurrev_202011_23858. PMID: 33275271. | C |
| Chou WP, Wang PW, Chen SL, Chang YP, Wu CF, Lu WH, Yen CF. Risk Perception, Protective Behaviors, and General Anxiety during the Coronavirus Disease 2019 Pandemic among Affiliated Health Care Professionals in Taiwan: Comparisons with Frontline Health Care Professionals and the General Public. Int J Environ Res Public Health. 2020 Dec 13;17(24):9329. doi: 10.3390/ijerph17249329. PMID: 33322220; PMCID: PMC7764763. | C |
| Christodoulou C, Efstathiou V, Michopoulos I, Ferentinos P, Korkoliakou P, Gkerekou M, Bouras G, Papadopoulou A, Papageorgiou C, Douzenis A. A case-control study of hopelessness and suicidal behavior in the city of Athens, Greece. The role of the financial crisis. Psychol Health Med. 2017 Aug;22(7):772-777. doi: 10.1080/13548506.2016.1164872. Epub 2016 Mar 30. PMID: 27028345. | C |
| Córdoba-Doña JA, Escolar-Pujolar A, San Sebastián M, Gustafsson PE. How are the employed and unemployed affected by the economic crisis in Spain? Educational inequalities, life conditions and mental health in a context of high unemployment. BMC Public Health. 2016 Mar 15;16:267. doi: 10.1186/s12889-016-2934-z. PMID: 26979336; PMCID: PMC4791891. | S |
| Costa, D. ;Cunha, M. ;Ferreira, C. ;Gama, A. ;Rodrigues, A. M. N. ;Rosado-Marques, V. ;Nogueira, H. ;Silva, M. R. G. ;Padez, C. The impact of the economic crisis on the mental health of Portuguese primary-school children. European Journal of Public Health. 2019. | C |
| Covid-19 and occupational health. Bull Acad Natl Med. 2020 Dec;204(9):e23. doi: 10.1016/j.banm.2020.05.038. Epub 2020 May 15. PMID: 32427148; PMCID: PMC7227575. | S |
| Cox RC, Jessup SC, Luber MJ, Olatunji BO. Pre-pandemic disgust proneness predicts increased coronavirus anxiety and safety behaviors: Evidence for a diathesis-stress model. J Anxiety Disord. 2020 Dec;76:102315. doi: 10.1016/j.janxdis.2020.102315. Epub 2020 Sep 22. PMID: 33007711; PMCID: PMC7507982. | S |
| Curtis S, Leonardi GS. Health, wealth and ways of life: what can we learn from the Swedish, US and UK experience? Overview. Soc Sci Med. 2012 Mar;74(5):639-42. doi: 10.1016/j.socscimed.2011.12.004. Epub 2011 Dec 24. PMID: 22200092. | S |
| da Silva FCT, Neto MLR. Psychiatric symptomatology associated with depression, anxiety, distress, and insomnia in health professionals working in patients affected by COVID-19: A systematic review with meta-analysis. Prog Neuropsychopharmacol Biol Psychiatry. 2021 Jan 10;104:110057. doi: 10.1016/j.pnpbp.2020.110057. Epub 2020 Aug 7. PMID: 32777327; PMCID: PMC7411383. | C |
| Dalwai SH, Rege KP. Psychosocial Wellness During the Covid- 19 Pandemic: Building an ARCH. Indian Pediatr. 2020 Dec 15;57(12):1189-1190. doi: 10.1007/s13312-020-2083-4. PMID: 33318334; PMCID: PMC7781813. | S |
| Daly M, Robinson E. Psychological distress and adaptation to the COVID-19 crisis in the United States. J Psychiatr Res. 2021 Apr;136:603-609. doi: 10.1016/j.jpsychires.2020.10.035. Epub 2020 Oct 27. PMID: 33138985; PMCID: PMC7588823. | C |
| De Brier N, Stroobants S, Vandekerckhove P, De Buck E. Factors affecting mental health of health care workers during coronavirus disease outbreaks (SARS, MERS & COVID-19): A rapid systematic review. PLoS One. 2020 Dec 15;15(12):e0244052. doi: 10.1371/journal.pone.0244052. PMID: 33320910; PMCID: PMC7737991. | S |
| de Matos, D. G. ;Aidar, F. J. ;De Almeida, P. F. ;Moreira, O. C. ;de Souza, R. F. ;Marcal, A. C. ;Marcucci-Barbosa, L. S. ;Martins, F. D. ;Lobo, L. F. ;dos Santos, J. L. ;Guerra, I. ;Silva, Adce ;Neves, E. B. ;Cabral, Bgdt ;Reis, V. M. ;Nunes-Silva, A. The Impact of Measures Recommended by the Government to Limit the Spread of Coronavirus (COVID-19) on Physical Activity Levels, Quality of Life, and Mental Health of Brazilians. Sustainability. 2020. doi 10.3390/su12219072 | C |
| De Vogli R, Marmot M, Stuckler D. Strong evidence that the economic crisis caused a rise in suicides in Europe: the need for social protection. J Epidemiol Community Health. 2013 Apr;67(4):298. doi: 10.1136/jech-2012-202112. Epub 2013 Jan 15. PMID: 23322855. | C |
| Demirci Ş, Konca M, Yetim B, İlgün G. Effect of economic crisis on suicide cases: An ARDL bounds testing approach. Int J Soc Psychiatry. 2020 Feb;66(1):34-40. doi: 10.1177/0020764019879946. Epub 2019 Sep 30. PMID: 31564187. | C |
| Di Tella M, Romeo A, Benfante A, Castelli L. Mental health of healthcare workers during the COVID-19 pandemic in Italy. J Eval Clin Pract. 2020 Dec;26(6):1583-1587. doi: 10.1111/jep.13444. Epub 2020 Jul 25. PMID: 32710481. | C |
| Drydakis N. The effect of unemployment on self-reported health and mental health in Greece from 2008 to 2013: a longitudinal study before and during the financial crisis. Soc Sci Med. 2015 Mar;128:43-51. doi: 10.1016/j.socscimed.2014.12.025. Epub 2014 Dec 24. PMID: 25589031. | C |
| Du L, Chen YM, Li Y, Yuan W, Wang JS. Prevalence of depression during the SARS, MERS, and COVID-19 pandemics: A protocol for overview of systematic reviews. Medicine (Baltimore). 2020 Sep 18;99(38):e22235. doi: 10.1097/MD.0000000000022235. PMID: 32957366; PMCID: PMC7505281. | C |
| Duan H, Yan L, Ding X, Gan Y, Kohn N, Wu J. Impact of the COVID-19 pandemic on mental health in the general Chinese population: Changes, predictors and psychosocial correlates. Psychiatry Res. 2020 Nov;293:113396. doi: 10.1016/j.psychres.2020.113396. Epub 2020 Aug 18. PMID: 32861096; PMCID: PMC7431364. | C |
| Economou M, Madianos M, Peppou LE, Patelakis A, Stefanis CN. Major depression in the era of economic crisis: a replication of a cross-sectional study across Greece. J Affect Disord. 2013 Mar 5;145(3):308-14. doi: 10.1016/j.jad.2012.08.008. Epub 2012 Aug 30. PMID: 22939388. | C |
| Economou M, Madianos M, Peppou LE, Theleritis C, Patelakis A, Stefanis C. Suicidal ideation and reported suicide attempts in Greece during the economic crisis. World Psychiatry. 2013 Feb;12(1):53-9. doi: 10.1002/wps.20016. PMID: 23471802; PMCID: PMC3619166. | C |
| Economou, M. The Untoward Effects Of Economic Crisis On Mental Health In Greece: An Overview. 2014. Pluralism in Psychiatry: I. Diverse Approaches and Converging Goals. P 213-215. | C |
| Ergenekon AP, Yilmaz Yegit C, Cenk M, Bas Ikizoglu N, Atag E, Gokdemir Y, Erdem Eralp E, Karadag B. Depression and anxiety in mothers of home ventilated children before and during COVID-19 pandemic. Pediatr Pulmonol. 2021 Jan;56(1):264-270. doi: 10.1002/ppul.25107. Epub 2020 Oct 29. PMID: 33026707; PMCID: PMC7675723. | C |
| Escolà-Gascón Á, Marín FX, Rusiñol J, Gallifa J. Pseudoscientific beliefs and psychopathological risks increase after COVID-19 social quarantine. Global Health. 2020 Jul 30;16(1):72. doi: 10.1186/s12992-020-00603-1. PMID: 32731864; PMCID: PMC7391050. | C |
| Expression of concern: Chinese mental health burden during the COVID-19 pandemic. Asian J Psychiatr. 2020 Aug;52:102409. doi: 10.1016/j.ajp.2020.102409. Epub 2020 Sep 3. PMID: 32928691; PMCID: PMC7468332. | S |
| Feter N, Caputo EL, Doring IR, Leite JS, Cassuriaga J, Reichert FF, da Silva MC, Coombes JS, Rombaldi AJ. Sharp increase in depression and anxiety among Brazilian adults during the COVID-19 pandemic: findings from the PAMPA cohort. Public Health. 2021 Jan;190:101-107. doi: 10.1016/j.puhe.2020.11.013. Epub 2020 Dec 31. PMID: 33387848; PMCID: PMC7773543. | C |
| Fiorillo A, Gorwood P. The consequences of the COVID-19 pandemic on mental health and implications for clinical practice. Eur Psychiatry. 2020 Apr 1;63(1):e32. doi: 10.1192/j.eurpsy.2020.35. PMID: 32234102; PMCID: PMC7156565. | S |
| Fontes WHA, Gonçalves Júnior J, de Vasconcelos CAC, da Silva CGL, Gadelha MSV. Impacts of the SARS-CoV-2 Pandemic on the Mental Health of the Elderly. Front Psychiatry. 2020 Aug 17;11:841. doi: 10.3389/fpsyt.2020.00841. PMID: 32973583; PMCID: PMC7461950. | C |
| Fontova-Almató A, Suñer-Soler R, Salleras-Duran L, Bertran-Noguer C, Congost-Devesa L, Ferrer-Padrosa M, Juvinyà-Canal D. Evolution of Job Satisfaction and Burnout Levels of Emergency Department Professionals during a Period of Economic Recession. Int J Environ Res Public Health. 2020 Feb 2;17(3):921. doi: 10.3390/ijerph17030921. PMID: 32024249; PMCID: PMC7037686. | C |
| Fornell B, Correa M, López Del Amo MP, Martín JJ. Influence of changes in the Spanish labor market during the economic crisis (2007-2011) on perceived health. Qual Life Res. 2018 Aug;27(8):2095-2105. doi: 10.1007/s11136-018-1824-5. Epub 2018 Feb 24. PMID: 29478131. | C |
| Freedland KE, Dew MA, Sarwer DB, Burg MM, Hart TA, Ewing SWF, Fang CY, Blozis SA, Puterman E, Marquez B, Kaufmann PG. Health psychology in the time of COVID-19. Health Psychol. 2020 Dec;39(12):1021-1025. doi: 10.1037/hea0001049. PMID: 33252927. | S |
| Garriga M, Agasi I, Fedida E, Pinzón-Espinosa J, Vazquez M, Pacchiarotti I, Vieta E. The role of mental health home hospitalization care during the COVID-19 pandemic. Acta Psychiatr Scand. 2020 May;141(5):479-480. doi: 10.1111/acps.13173. PMID: 32279309; PMCID: PMC7262322. | P |
| Garrusi B, Amirkafi A, Garousi S. Mental health: the forgotten aspect of the COVID-19 pandemic. East Mediterr Health J. 2020 Oct 13;26(10):1151-1154. doi: 10.26719/emhj.20.117. PMID: 33103741. | C |
| Gassman-Pines A, Ananat EO, Fitz-Henley J 2nd. COVID-19 and Parent-Child Psychological Well-being. Pediatrics. 2020 Oct;146(4):e2020007294. doi: 10.1542/peds.2020-007294. Epub 2020 Aug 6. PMID: 32764151; PMCID: PMC7546085. | C |
| Gawai, J. P. ;Singh, S. ;Taksande, V. D. ;Sebastian, T. ;Kasturkar, P. ;Ankar, R. S. Critical Review on Impact of COVID 19 and Mental Health.  Journal of Evolution of Medical and Dental Sciences-Jemds. 2020. doi 10.14260/jemds/2020/470 | E/I |
| Ghosh A, Sarkar S. The coronavirus (COVID-19) pandemic's impact on maternal mental health and questionable healthcare services in rural India. Int J Health Plann Manage. 2020 Nov;35(6):1626-1628. doi: 10.1002/hpm.3050. Epub 2020 Sep 6. PMID: 32892430. | C |
| Giannopoulou I, Efstathiou V, Triantafyllou G, Korkoliakou P, Douzenis A. Adding stress to the stressed: Senior high school students' mental health amidst the COVID-19 nationwide lockdown in Greece. Psychiatry Res. 2021 Jan;295:113560. doi: 10.1016/j.psychres.2020.113560. Epub 2020 Nov 6. PMID: 33187723; PMCID: PMC7647459. | S |
| Gilan D, Röthke N, Blessin M, Kunzler A, Stoffers-Winterling J, Müssig M, Yuen KSL, Tüscher O, Thrul J, Kreuter F, Sprengholz P, Betsch C, Stieglitz RD, Lieb K. Psychomorbidity, Resilience, and Exacerbating and Protective Factors During the SARS-CoV-2 Pandemic. Dtsch Arztebl Int. 2020 Sep 18;117(38):625-630. doi: 10.3238/arztebl.2020.0625. PMID: 33200744; PMCID: PMC7817784. | C |
| Gili M, Roca M, Basu S, McKee M, Stuckler D. The mental health risks of economic crisis in Spain: evidence from primary care centres, 2006 and 2010. Eur J Public Health. 2013 Feb;23(1):103-8. doi: 10.1093/eurpub/cks035. Epub 2012 Apr 19. PMID: 23132877. | Other duplicate |
| Giordano A, Siciliano M, De Micco R, Sant'Elia V, Russo A, Tedeschi G, Tessitore A. Correlates of psychological distress in epileptic patients during the COVID-19 outbreak. Epilepsy Behav. 2021 Feb;115:107632. doi: 10.1016/j.yebeh.2020.107632. Epub 2020 Nov 19. PMID: 33373874; PMCID: PMC7674117. | C |
| Golberstein E, Wen H, Miller BF. Coronavirus Disease 2019 (COVID-19) and Mental Health for Children and Adolescents. JAMA Pediatr. 2020 Sep 1;174(9):819-820. doi: 10.1001/jamapediatrics.2020.1456. PMID: 32286618. | P |
| Gold JA. Covid-19: adverse mental health outcomes for healthcare workers. BMJ. 2020 May 5;369:m1815. doi: 10.1136/bmj.m1815. PMID: 32371465. | P |
| González-Sanguino C, Ausín B, Castellanos MÁ, Saiz J, López-Gómez A, Ugidos C, Muñoz M. Mental Health Consequences of the Coronavirus 2020 Pandemic (COVID-19) in Spain. A Longitudinal Study. Front Psychiatry. 2020 Nov 9;11:565474. doi: 10.3389/fpsyt.2020.565474. PMID: 33240123; PMCID: PMC7680731. | C |
| González-Sanguino C, Ausín B, Castellanos MA, Saiz J, Muñoz M. Mental health consequences of the Covid-19 outbreak in Spain. A longitudinal study of the alarm situation and return to the new normality. Prog Neuropsychopharmacol Biol Psychiatry. 2021 Apr 20;107:110219. doi: 10.1016/j.pnpbp.2020.110219. Epub 2020 Dec 15. PMID: 33338556; PMCID: PMC7833458. | C |
| Görlich Y, Stadelmann D. Mental Health of Flying Cabin Crews: Depression, Anxiety, and Stress Before and During the COVID-19 Pandemic. Front Psychol. 2020 Dec 17;11:581496. doi: 10.3389/fpsyg.2020.581496. PMID: 33391100; PMCID: PMC7773923. | C |
| Gotsens M, Malmusi D, Villarroel N, Vives-Cases C, Garcia-Subirats I, Hernando C, Borrell C. Health inequality between immigrants and natives in Spain: the loss of the healthy immigrant effect in times of economic crisis. Eur J Public Health. 2015 Dec;25(6):923-9. doi: 10.1093/eurpub/ckv126. Epub 2015 Jun 30. PMID: 26136466. | E/I |
| Gouttebarge V, Ahmad I, Mountjoy M, Rice S, Kerkhoffs G. Anxiety and Depressive Symptoms During the COVID-19 Emergency Period: A Comparative Cross-Sectional Study in Professional Football. Clin J Sport Med. 2020 Sep 15. doi: 10.1097/JSM.0000000000000886. Epub ahead of print. PMID: 32941374. | C |
| Graham, Carol ;Chattopadhyay, Soumya ;Picon, Mario. Adapting to Adversity: Happiness and the 2009 Economic Crisis in the United States.  Graduate Faculty of Political and Social Science of the New School University, New York NY. 2010. | C |
| Graupensperger S, Benson AJ, Kilmer JR, Evans MB. Social (Un)distancing: Teammate Interactions, Athletic Identity, and Mental Health of Student-Athletes During the COVID-19 Pandemic. J Adolesc Health. 2020 Nov;67(5):662-670. doi: 10.1016/j.jadohealth.2020.08.001. Epub 2020 Sep 14. PMID: 32943294; PMCID: PMC7489994. | C |
| Greenberg N, Docherty M, Gnanapragasam S, Wessely S. Managing mental health challenges faced by healthcare workers during covid-19 pandemic. BMJ. 2020 Mar 26;368:m1211. doi: 10.1136/bmj.m1211. PMID: 32217624. | P |
| Grubic N, Badovinac S, Johri AM. Student mental health in the midst of the COVID-19 pandemic: A call for further research and immediate solutions. Int J Soc Psychiatry. 2020 Aug;66(5):517-518. doi: 10.1177/0020764020925108. Epub 2020 May 2. PMID: 32364039; PMCID: PMC7405631. | C |
| Guillena, S. L. R. ;Saenz, B. M. ;Henche, E. G Economic crisis and suicide - Is it a social reality or just media sensationalism?  European Neuropsychopharmacology. 2014. | S |
| Gunawan J, Juthamanee S, Aungsuroch Y. Current Mental Health Issues in the Era of Covid-19. Asian J Psychiatr. 2020 Jun;51:102103. doi: 10.1016/j.ajp.2020.102103. Epub 2020 Apr 25. PMID: 32361675; PMCID: PMC7182519. | P |
| Gunnell D, Platt S, Hawton K. The economic crisis and suicide. BMJ. 2009 May 15;338:b1891. doi: 10.1136/bmj.b1891. PMID: 19447829. | P |
| Guo Q, Zheng Y, Shi J, Wang J, Li G, Li C, Fromson JA, Xu Y, Liu X, Xu H, Zhang T, Lu Y, Chen X, Hu H, Tang Y, Yang S, Zhou H, Wang X, Chen H, Wang Z, Yang Z. Immediate psychological distress in quarantined patients with COVID-19 and its association with peripheral inflammation: A mixed-method study. Brain Behav Immun. 2020 Aug;88:17-27. doi: 10.1016/j.bbi.2020.05.038. Epub 2020 May 19. PMID: 32416290; PMCID: PMC7235603. | S |
| Gupta, T. ;Nebhinani, N. Impact of COVID-19 Pandemic on Child and Adolescent Mental Health. Journal of Indian Association for Child and Adolescent Mental Health. 2020 | P |
| Hacimusalar Y, Kahve AC, Yasar AB, Aydin MS. Anxiety and hopelessness levels in COVID-19 pandemic: A comparative study of healthcare professionals and other community sample in Turkey. J Psychiatr Res. 2020 Oct;129:181-188. doi: 10.1016/j.jpsychires.2020.07.024. Epub 2020 Jul 21. PMID: 32758711; PMCID: PMC7372275. | C |
| Hageman JR. Children's and Families' Behavioral and Mental Health During COVID-19. Pediatr Ann. 2020 Oct 1;49(10):e405-e406. doi: 10.3928/19382359-20200922-05. PMID: 33034652. | P |
| Halvorsen E, Stamu-O'Brien C, Carniciu S, Jafferany M. Psychological effects of COVID-19 on parenting and maternal-fetal mental health. Dermatol Ther. 2020 Jul;33(4):e13579. doi: 10.1111/dth.13579. Epub 2020 Jun 7. PMID: 32406114; PMCID: PMC7272902. | P |
| Hamada K, Fan X. The impact of COVID-19 on individuals living with serious mental illness. Schizophr Res. 2020 Aug;222:3-5. doi: 10.1016/j.schres.2020.05.054. Epub 2020 May 27. PMID: 32473931; PMCID: PMC7250778. | P |
| Hamm ME, Brown PJ, Karp JF, Lenard E, Cameron F, Dawdani A, Lavretsky H, Miller JP, Mulsant BH, Pham VT, Reynolds CF, Roose SP, Lenze EJ. Experiences of American Older Adults with Pre-existing Depression During the Beginnings of the COVID-19 Pandemic: A Multicity, Mixed-Methods Study. Am J Geriatr Psychiatry. 2020 Sep;28(9):924-932. doi: 10.1016/j.jagp.2020.06.013. Epub 2020 Jun 20. PMID: 32682619; PMCID: PMC7305766. | S |
| Hao X, Zhou D, Li Z, Zeng G, Hao N, Li E, Li W, Deng A, Lin M, Yan B. Severe psychological distress among patients with epilepsy during the COVID-19 outbreak in southwest China. Epilepsia. 2020 Jun;61(6):1166-1173. doi: 10.1111/epi.16544. Epub 2020 May 22. PMID: 32353184; PMCID: PMC7267575. | C |
| Hao X, Zhou D, Li Z, Zeng G, Hao N, Li E, Li W, Deng A, Lin M, Yan B. Severe psychological distress among patients with epilepsy during the COVID-19 outbreak in southwest China. Epilepsia. 2020 Jun;61(6):1166-1173. doi: 10.1111/epi.16544. Epub 2020 May 22. PMID: 32353184; PMCID: PMC7267575. | Other duplicate |
| Haravuori H, Junttila K, Haapa T, Tuisku K, Kujala A, Rosenström T, Suvisaari J, Pukkala E, Laukkala T, Jylhä P. Personnel Well-Being in the Helsinki University Hospital during the COVID-19 Pandemic-A Prospective Cohort Study. Int J Environ Res Public Health. 2020 Oct 28;17(21):7905. doi: 10.3390/ijerph17217905. PMID: 33126583; PMCID: PMC7662585. | C |
| Hassannia L, Taghizadeh F, Moosazadeh M, Zarghami M, Taghizadeh H, Dooki AF, Fathi M, Alizadeh-Navaei R, Hedayatizadeh-Omran A, Dehghan N. Anxiety and Depression in Health Workers and General Population During COVID-19 in IRAN: A Cross-Sectional Study. Neuropsychopharmacol Rep. 2021 Mar;41(1):40-49. doi: 10.1002/npr2.12153. Epub 2020 Dec 25. PMID: 33369264. | C |
| Hessami K, Romanelli C, Chiurazzi M, Cozzolino M. COVID-19 pandemic and maternal mental health: a systematic review and meta-analysis. J Matern Fetal Neonatal Med. 2020 Nov 1:1-8. doi: 10.1080/14767058.2020.1843155. Epub ahead of print. PMID: 33135523. | C |
| Hessel P, Vandoros S, Avendano M. The differential impact of the financial crisis on health in Ireland and Greece: a quasi-experimental approach. Public Health. 2014 Oct;128(10):911-9. doi: 10.1016/j.puhe.2014.08.004. Epub 2014 Oct 18. PMID: 25369355; PMCID: PMC4393742. | S |
| Hidalgo MD, Balluerka N, Gorostiaga A, Espada JP, Santed MÁ, Padilla JL, Gómez-Benito J. The Psychological Consequences of COVID-19 and Lockdown in the Spanish Population: An Exploratory Sequential Design. Int J Environ Res Public Health. 2020 Nov 19;17(22):8578. doi: 10.3390/ijerph17228578. PMID: 33227938; PMCID: PMC7699190. | S |
| Hiremath P, Suhas Kowshik CS, Manjunath M, Shettar M. COVID 19: Impact of lock-down on mental health and tips to overcome. Asian J Psychiatr. 2020 Jun;51:102088. doi: 10.1016/j.ajp.2020.102088. Epub 2020 Apr 10. PMID: 32302964; PMCID: PMC7151434. | C |
| Hong J, Knapp M, McGuire A. Income-related inequalities in the prevalence of depression and suicidal behaviour: a 10-year trend following economic crisis. World Psychiatry. 2011 Feb;10(1):40-4. doi: 10.1002/j.2051-5545.2011.tb00012.x. PMID: 21379355; PMCID: PMC3048505. | E/I |
| Hossain MM, Tasnim S, Sultana A, Faizah F, Mazumder H, Zou L, McKyer ELJ, Ahmed HU, Ma P. Epidemiology of mental health problems in COVID-19: a review. F1000Res. 2020 Jun 23;9:636. doi: 10.12688/f1000research.24457.1. PMID: 33093946; PMCID: PMC7549174. | S |
| Houle JN, Light MT. The harder they fall? Sex and race/ethnic specific suicide rates in the U.S. foreclosure crisis. Soc Sci Med. 2017 May;180:114-124. doi: 10.1016/j.socscimed.2017.03.033. Epub 2017 Mar 19. PMID: 28343110; PMCID: PMC6076432. | E/I |
| Huckins JF, daSilva AW, Wang W, Hedlund E, Rogers C, Nepal SK, Wu J, Obuchi M, Murphy EI, Meyer ML, Wagner DD, Holtzheimer PE, Campbell AT. Mental Health and Behavior of College Students During the Early Phases of the COVID-19 Pandemic: Longitudinal Smartphone and Ecological Momentary Assessment Study. J Med Internet Res. 2020 Jun 17;22(6):e20185. doi: 10.2196/20185. PMID: 32519963; PMCID: PMC7301687. | C |
| Iqbal, S. Z. ;Li, B. ;Onigu-Otito, E. ;Naqvi, M. F. ;Shah, A. A. The Long-Term Mental Health Effects of COVID-19. Psychiatric Annals. 2020. doi 10.3928/00485713-20201103-01 | C |
| Javed B, Sarwer A, Soto EB, Mashwani ZU. Impact of SARS-CoV-2 (Coronavirus) Pandemic on Public Mental Health. Front Public Health. 2020 Jun 23;8:292. doi: 10.3389/fpubh.2020.00292. PMID: 32656175; PMCID: PMC7324781. | P |
| Ji G, Wei W, Yue KC, Li H, Shi LJ, Ma JD, He CY, Zhou SS, Zhao Z, Lou T, Cheng J, Yang SC, Hu XZ. Effects of the COVID-19 Pandemic on Obsessive-Compulsive Symptoms Among University Students: Prospective Cohort Survey Study. J Med Internet Res. 2020 Sep 30;22(9):e21915. doi: 10.2196/21915. PMID: 32931444; PMCID: PMC7528732. | S |
| John-Henderson NA, Ginty AT. Historical trauma and social support as predictors of psychological stress responses in American Indian adults during the COVID-19 pandemic. J Psychosom Res. 2020 Dec;139:110263. doi: 10.1016/j.jpsychores.2020.110263. Epub 2020 Oct 2. PMID: 33038816; PMCID: PMC7531919. | C |
| José Celada F, Quiroga-Fernández A, Mohedano-Moriano A, Aliaga Vera I, Fernández Pérez C, Martín Conty JL. Attempted suicides attended by emergency services during the economic recession in Castile-La Mancha, Spain. Emergencias. 2017 Jul;29(4):247-252. English, Spanish. PMID: 30033698. | Other language |
| Jun J, Tucker S, Melnyk BM. Clinician Mental Health and Well-Being During Global Healthcare Crises: Evidence Learned From Prior Epidemics for COVID-19 Pandemic. Worldviews Evid Based Nurs. 2020 Jun;17(3):182-184. doi: 10.1111/wvn.12439. Epub 2020 Apr 22. PMID: 32246793. | C |
| Jung S, Kneer J, Krüger THC. Mental Health, Sense of Coherence, and Interpersonal Violence during the COVID-19 Pandemic Lockdown in Germany. J Clin Med. 2020 Nov 18;9(11):3708. doi: 10.3390/jcm9113708. PMID: 33218215; PMCID: PMC7699150. | C |
| Kang, S. M. COVID-19 and health-care worker mental health. Lancet Psychiatry. 2020 | C |
| Kannampallil TG, Goss CW, Evanoff BA, Strickland JR, McAlister RP, Duncan J. Exposure to COVID-19 patients increases physician trainee stress and burnout. PLoS One. 2020 Aug 6;15(8):e0237301. doi: 10.1371/journal.pone.0237301. PMID: 32760131; PMCID: PMC7410237. | C |
| Kar SK, Oyetunji TP, Prakash AJ, Ogunmola OA, Tripathy S, Lawal MM, Sanusi ZK, Arafat SMY. Mental health research in the lower-middle-income countries of Africa and Asia during the COVID-19 pandemic: A scoping review. Neurol Psychiatry Brain Res. 2020 Dec;38:54-64. doi: 10.1016/j.npbr.2020.10.003. Epub 2020 Oct 28. PMID: 33162686; PMCID: PMC7598562. | S |
| Karahan I. The COVID-19 anxiety of hospitalised patients in a palliative care unit. Int J Palliat Nurs. 2020 Aug 2;26(6):267. doi: 10.12968/ijpn.2020.26.6.267. PMID: 32841081. | C |
| Kathirvel N. Post COVID-19 pandemic mental health challenges. Asian J Psychiatr. 2020 Oct;53:102430. doi: 10.1016/j.ajp.2020.102430. Epub 2020 Sep 22. PMID: 33264840; PMCID: PMC7507979. | C |
| Khan KS, Mamun MA, Griffiths MD, Ullah I. The Mental Health Impact of the COVID-19 Pandemic Across Different Cohorts. Int J Ment Health Addict. 2020 Jul 9:1-7. doi: 10.1007/s11469-020-00367-0. Epub ahead of print. PMID: 32837440; PMCID: PMC7347045. | C |
| Khan S, Siddique R, Li Z, Xue M, Liu J, Nabi G. COVID-19 pandemic; prevention, treatment, and mental health. Hum Vaccin Immunother. 2020 Sep 1;16(9):2215-2216. doi: 10.1080/21645515.2020.1759976. Epub 2020 May 21. PMID: 32437231; PMCID: PMC7553693. | C |
| Khan S, Siddique R, Xiaoyan W, Zhang R, Nabi G, Sohail Afzal M, Liu J, Xue M. Mental health consequences of infections by coronaviruses including severe acute respiratory syndrome coronavirus 2 (SARS-CoV-2). Brain Behav. 2021 Feb;11(2):e01901. doi: 10.1002/brb3.1901. Epub 2020 Dec 13. PMID: 33314732; PMCID: PMC7882169. | C |
| Kikuchi H, Machida M, Nakamura I, Saito R, Odagiri Y, Kojima T, Watanabe H, Fukui K, Inoue S. Changes in Psychological Distress During the COVID-19 Pandemic in Japan: A Longitudinal Study. J Epidemiol. 2020 Nov 5;30(11):522-528. doi: 10.2188/jea.JE20200271. Epub 2020 Sep 19. PMID: 32963212; PMCID: PMC7557175. | C |
| Kim AW, Nyengerai T, Mendenhall E. Evaluating the Mental Health Impacts of the COVID-19 Pandemic in Urban South Africa: Perceived Risk of COVID-19 Infection and Childhood Trauma Predict Adult Depressive Symptoms. medRxiv [Preprint]. 2020 Jun 16:2020.06.13.20130120. doi: 10.1101/2020.06.13.20130120. Update in: Psychol Med. 2020 Sep 08;:1-24. PMID: 32587996; PMCID: PMC7310654. | C |
| Kim AW, Nyengerai T, Mendenhall E. Evaluating the mental health impacts of the COVID-19 pandemic: perceived risk of COVID-19 infection and childhood trauma predict adult depressive symptoms in urban South Africa. Psychol Med. 2020 Sep 8:1-13. doi: 10.1017/S0033291720003414. Epub ahead of print. PMID: 32895082; PMCID: PMC7520640. | C |
| Kim H, Song YJ, Yi JJ, Chung WJ, Nam CM. Changes in mortality after the recent economic crisis in South Korea. Ann Epidemiol. 2004 Jul;14(6):442-6. doi: 10.1016/j.annepidem.2003.09.018. PMID: 15246334. | E/I |
| Kim HH, Jung JH. Social Isolation and Psychological Distress During the COVID-19 Pandemic: A Cross-National Analysis. Gerontologist. 2021 Jan 21;61(1):103-113. doi: 10.1093/geront/gnaa168. PMID: 33125065; PMCID: PMC7665475. | C |
| Kim HH, Jung JH. Social Isolation and Psychological Distress During the COVID-19 Pandemic: A Cross-National Analysis. Gerontologist. 2021 Jan 21;61(1):103-113. doi: 10.1093/geront/gnaa168. PMID: 33125065; PMCID: PMC7665475. | C |
| Kim YJ, Qian L, Aslam MS. The impact of substance use disorder on the mental health among COVID-19 patients: A protocol for systematic review and meta-analysis. Medicine (Baltimore). 2020 Nov 13;99(46):e23203. doi: 10.1097/MD.0000000000023203. PMID: 33181701; PMCID: PMC7668455. | C |
| Knipe D, Evans H, Marchant A, Gunnell D, John A. Mapping population mental health concerns related to COVID-19 and the consequences of physical distancing: a Google trends analysis. Wellcome Open Res. 2020 Jun 10;5:82. doi: 10.12688/wellcomeopenres.15870.2. PMID: 32671230; PMCID: PMC7331103. | C |
| Kondilis E, Giannakopoulos S, Gavana M, Ierodiakonou I, Waitzkin H, Benos A. Economic crisis, restrictive policies, and the population's health and health care: the Greek case. Am J Public Health. 2013 Jun;103(6):973-9. doi: 10.2105/AJPH.2012.301126. Epub 2013 Apr 18. PMID: 23597358; PMCID: PMC3698730. | S |
| Konstantinov V, Berdenova S, Satkangulova G, Reznik A, Isralowitz R. COVID-19 Impact on Kazakhstan University Student Fear, Mental Health, and Substance Use. Int J Ment Health Addict. 2020 Nov 9:1-7. doi: 10.1007/s11469-020-00412-y. Epub ahead of print. PMID: 33192199; PMCID: PMC7651818. | C |
| Kounou KB, Guédénon KM, Dogbe Foli AA, Gnassounou-Akpa E. Mental health of medical professionals during the COVID-19 pandemic in Togo. Psychiatry Clin Neurosci. 2020 Oct;74(10):559-560. doi: 10.1111/pcn.13108. Epub 2020 Jul 31. PMID: 32621390; PMCID: PMC7361459. | C |
| Kovler ML, Ziegfeld S, Ryan LM, Goldstein MA, Gardner R, Garcia AV, Nasr IW. Increased proportion of physical child abuse injuries at a level I pediatric trauma center during the Covid-19 pandemic. Child Abuse Negl. 2021 Jun;116(Pt 2):104756. doi: 10.1016/j.chiabu.2020.104756. Epub 2020 Sep 25. PMID: 33004213; PMCID: PMC7518108. | S |
| Kozman D, Graziul C, Gibbons R, Alexander GC. Association between unemployment rates and prescription drug utilization in the United States, 2007-2010. BMC Health Serv Res. 2012 Nov 30;12:435. doi: 10.1186/1472-6963-12-435. PMID: 23193954; PMCID: PMC3541063. | C |
| Krishnamoorthy Y, Nagarajan R, Saya GK, Menon V. Prevalence of psychological morbidities among general population, healthcare workers and COVID-19 patients amidst the COVID-19 pandemic: A systematic review and meta-analysis. Psychiatry Res. 2020 Nov;293:113382. doi: 10.1016/j.psychres.2020.113382. Epub 2020 Aug 11. PMID: 32829073; PMCID: PMC7417292. | C |
| Krishnamoorthy Y, Nagarajan R, Saya GK, Menon V. Prevalence of psychological morbidities among general population, healthcare workers and COVID-19 patients amidst the COVID-19 pandemic: A systematic review and meta-analysis. Psychiatry Res. 2020 Nov;293:113382. doi: 10.1016/j.psychres.2020.113382. Epub 2020 Aug 11. PMID: 32829073; PMCID: PMC7417292. | S |
| Kuehn BM. Pandemic's Mental Health Toll Grows. JAMA. 2020 Sep 22;324(12):1130. doi: 10.1001/jama.2020.17280. PMID: 32960260. | C |
| Kuki K, Yamaguchi Y, Makinodan M, Honda M, Ueda J, Okazaki K, Okamura K, Kimoto S, Kishimoto T. Effects of contact with COVID-19 patients on the mental health of workers in a psychiatric hospital. Psychiatry Clin Neurosci. 2021 Feb;75(2):67-69. doi: 10.1111/pcn.13179. Epub 2020 Dec 14. PMID: 33247505; PMCID: PMC7753672. | C |
| Kumar A, Nayar KR. COVID 19 and its mental health consequences. J Ment Health. 2021 Feb;30(1):1-2. doi: 10.1080/09638237.2020.1757052. Epub 2020 Apr 27. PMID: 32339041. | C |
| Kumar M, Kumar P. Impact of pandemic on mental health in lower- and middle-income countries (LMICs). Glob Ment Health (Camb). 2020 Dec 3;7:e35. doi: 10.1017/gmh.2020.28. PMID: 34191999; PMCID: PMC7750653. | C |
| Lai AY, Lee L, Wang MP, Feng Y, Lai TT, Ho LM, Lam VS, Ip MS, Lam TH. Mental Health Impacts of the COVID-19 Pandemic on International University Students, Related Stressors, and Coping Strategies. Front Psychiatry. 2020 Nov 23;11:584240. doi: 10.3389/fpsyt.2020.584240. PMID: 33329126; PMCID: PMC7719620. | C |
| Lara B, Carnes A, Dakterzada F, Benitez I, Piñol-Ripoll G. Neuropsychiatric symptoms and quality of life in Spanish patients with Alzheimer's disease during the COVID-19 lockdown. Eur J Neurol. 2020 Sep;27(9):1744-1747. doi: 10.1111/ene.14339. Epub 2020 Jun 24. PMID: 32449791; PMCID: PMC7283827. | C |
| Leaune E, Samuel M, Oh H, Poulet E, Brunelin J. Suicidal behaviors and ideation during emerging viral disease outbreaks before the COVID-19 pandemic: A systematic rapid review. Prev Med. 2020 Dec;141:106264. doi: 10.1016/j.ypmed.2020.106264. Epub 2020 Oct 2. PMID: 33017599; PMCID: PMC7531915. | S |
| Lee AM, Wong JG, McAlonan GM, Cheung V, Cheung C, Sham PC, Chu CM, Wong PC, Tsang KW, Chua SE. Stress and psychological distress among SARS survivors 1 year after the outbreak. Can J Psychiatry. 2007 Apr;52(4):233-40. doi: 10.1177/070674370705200405. PMID: 17500304. | C |
| Lee J. Mental health effects of school closures during COVID-19. Lancet Child Adolesc Health. 2020 Jun;4(6):421. doi: 10.1016/S2352-4642(20)30109-7. Epub 2020 Apr 14. Erratum in: Lancet Child Adolesc Health. 2020 Apr 17;: PMID: 32302537; PMCID: PMC7156240. | C |
| Lee J. Mental health effects of school closures during COVID-19. Lancet Child Adolesc Health. 2020 Jun;4(6):421. doi: 10.1016/S2352-4642(20)30109-7. Epub 2020 Apr 14. Erratum in: Lancet Child Adolesc Health. 2020 Apr 17;: PMID: 32302537; PMCID: PMC7156240. | Other duplicate |
| Lee MCC, Thampi S, Chan HP, Khoo D, Chin BZB, Foo DPX, Lua CB, Lewin B, Jacob R. Psychological distress during the COVID-19 pandemic amongst anaesthesiologists and nurses. Br J Anaesth. 2020 Oct;125(4):e384-e386. doi: 10.1016/j.bja.2020.07.005. Epub 2020 Jul 22. PMID: 32792139; PMCID: PMC7375332. | C |
| Lee S, Guo WJ, Tsang A, Mak AD, Wu J, Ng KL, Kwok K. Evidence for the 2008 economic crisis exacerbating depression in Hong Kong. J Affect Disord. 2010 Oct;126(1-2):125-33. doi: 10.1016/j.jad.2010.03.007. Epub 2010 Apr 8. PMID: 20381157. | C |
| Lee, J. Features Mental health effects of school closures during COVID-19. Lancet Child & Adolescent Health. 2020. doi 10.1016/s2352-4642(20)30109-7 | C |
| Lei L, Huang X, Zhang S, Yang J, Yang L, Xu M. Comparison of Prevalence and Associated Factors of Anxiety and Depression Among People Affected by versus People Unaffected by Quarantine During the COVID-19 Epidemic in Southwestern China. Med Sci Monit. 2020 Apr 26;26:e924609. doi: 10.12659/MSM.924609. PMID: 32335579; PMCID: PMC7199435. | C |
| Li J, Yang Z, Qiu H, Wang Y, Jian L, Ji J, Li K. Anxiety and depression among general population in China at the peak of the COVID-19 epidemic. World Psychiatry. 2020 Jun;19(2):249-250. doi: 10.1002/wps.20758. PMID: 32394560; PMCID: PMC7214959. | C |
| Li S, Wang Y, Xue J, Zhao N, Zhu T. The Impact of COVID-19 Epidemic Declaration on Psychological Consequences: A Study on Active Weibo Users. Int J Environ Res Public Health. 2020 Mar 19;17(6):2032. doi: 10.3390/ijerph17062032. PMID: 32204411; PMCID: PMC7143846. | O |
| Liang Y, Wu K, Zhou Y, Huang X, Zhou Y, Liu Z. Mental Health in Frontline Medical Workers during the 2019 Novel Coronavirus Disease Epidemic in China: A Comparison with the General Population. Int J Environ Res Public Health. 2020 Sep 9;17(18):6550. doi: 10.3390/ijerph17186550. PMID: 32916836; PMCID: PMC7558595. | C |
| Librero J, Segura A, Beatriz LV. Suicides, hurricanes and economic crisis. Eur J Public Health. 2014 Apr;24(2):183. doi: 10.1093/eurpub/ckt167. Epub 2013 Nov 5. PMID: 24192399. | S |
| Lim JM, Tun ZM, Kumar V, Quaye SED, Offeddu V, Cook AR, Lwin MO, Jiang S, Tam CC. Population anxiety and positive behaviour change during the COVID-19 epidemic: Cross-sectional surveys in Singapore, China and Italy. Influenza Other Respir Viruses. 2021 Jan;15(1):45-55. doi: 10.1111/irv.12785. Epub 2020 Sep 5. PMID: 32889784; PMCID: PMC7767950. | C |
| Lin YH, Chiang TW, Lin YL. Increased Internet Searches for Insomnia as an Indicator of Global Mental Health During the COVID-19 Pandemic: Multinational Longitudinal Study. J Med Internet Res. 2020 Sep 21;22(9):e22181. doi: 10.2196/22181. PMID: 32924951; PMCID: PMC7508633. | O |
| Liu Y, Mattke S. Association between state stay-at-home orders and risk reduction behaviors and mental distress amid the COVID-19 pandemic. Prev Med. 2020 Dec;141:106299. doi: 10.1016/j.ypmed.2020.106299. Epub 2020 Oct 28. PMID: 33129909; PMCID: PMC7598555. | C |
| Llibre-Guerra JJ, Jiménez-Velázquez IZ, Llibre-Rodriguez JJ, Acosta D. The impact of COVID-19 on mental health in the Hispanic Caribbean region. Int Psychogeriatr. 2020 Oct;32(10):1143-1146. doi: 10.1017/S1041610220000848. Epub 2020 May 8. PMID: 32383637; PMCID: PMC7267095. | C |
| Lo CC, Cheng TC. Race, unemployment rate, and chronic mental illness: a 15-year trend analysis. Soc Psychiatry Psychiatr Epidemiol. 2014 Jul;49(7):1119-28. doi: 10.1007/s00127-014-0844-x. Epub 2014 Feb 21. PMID: 24556812. | E/I |
| Loades ME, Chatburn E, Higson-Sweeney N, Reynolds S, Shafran R, Brigden A, Linney C, McManus MN, Borwick C, Crawley E. Rapid Systematic Review: The Impact of Social Isolation and Loneliness on the Mental Health of Children and Adolescents in the Context of COVID-19. J Am Acad Child Adolesc Psychiatry. 2020 Nov;59(11):1218-1239.e3. doi: 10.1016/j.jaac.2020.05.009. Epub 2020 Jun 3. PMID: 32504808; PMCID: PMC7267797. | C |
| Longobardi C, Morese R, Fabris MA. COVID-19 Emergency: Social Distancing and Social Exclusion as Risks for Suicide Ideation and Attempts in Adolescents. Front Psychol. 2020 Nov 19;11:551113. doi: 10.3389/fpsyg.2020.551113. PMID: 33329182; PMCID: PMC7710515. | P |
| Lu W, Wang H, Lin Y, Li L. Psychological status of medical workforce during the COVID-19 pandemic: A cross-sectional study. Psychiatry Res. 2020 Jun;288:112936. doi: 10.1016/j.psychres.2020.112936. Epub 2020 Apr 4. PMID: 32276196; PMCID: PMC7195354. | C |
| Luca, L. ;Ciubara, A. B. ;Fulga, I. ;Burlea, S. L. ;Terpan, M. ;Ciubara, A. Social Implications for Psychiatric Pathology of Depressive and Anxiety Disorders, Alcohol Addiction and Psychotic Disorders during the COVID-19 Pandemic in Romania. Analysis of two Relevant Psychiatry Hospitals. Revista De Cercetare Si Interventie Sociala. 2020. | C |
| Lung FW, Lu YC, Chang YY, Shu BC. Mental Symptoms in Different Health Professionals During the SARS Attack: A Follow-up Study. Psychiatr Q. 2009 Jun;80(2):107-16. doi: 10.1007/s11126-009-9095-5. Epub 2009 Feb 27. PMID: 19247834. | C |
| Luo Y, Chua CR, Xiong Z, Ho RC, Ho CSH. A Systematic Review of the Impact of Viral Respiratory Epidemics on Mental Health: An Implication on the Coronavirus Disease 2019 Pandemic. Front Psychiatry. 2020 Nov 23;11:565098. doi: 10.3389/fpsyt.2020.565098. PMID: 33329106; PMCID: PMC7719673. | S |
| Lyrakos, G. ;Grigoriadou, M. ;Zacharis, T. ;Grigoraki, M. ;Menti, D. ;Tsioumas, N. ;Georgiadis, A. ;Spyropoulos, I. ;Spinaris, V. The effect of the reductions in social interactions due to the economic crisis on the subjective well-being of non-insurance health care seekers in Greece. European Psychiatry. 2016. doi 10.1016/j.eurpsy.2016.01.442 | C |
| Machado DB, Pescarini JM, Araújo LFSC, Barreto ML. Austerity policies in Brazil may affect violence related outcomes. Cien Saude Colet. 2019 Dec;24(12):4385-4394. doi: 10.1590/1413-812320182412.07422019. Epub 2019 Aug 29. PMID: 31778489. | S |
| Malathesh BC, Chatterjee SS, Das S. Overview of mental health issues of COVID-19: need of the hour. Gen Psychiatr. 2020 May;33(3):e100233. doi: 10.1136/gpsych-2020-100233. Epub 2020 May 20. PMID: 34192230; PMCID: PMC7295860. | P |
| Mari JJ, Oquendo MA. Mental health consequences of COVID-19: the next global pandemic. Trends Psychiatry Psychother. 2020 Jul-Sep;42(3):219-220. doi: 10.1590/2237-6089-2020-0081. PMID: 32844977; PMCID: PMC7879073. | P |
| Marques de Miranda D, da Silva Athanasio B, Sena Oliveira AC, Simoes-E-Silva AC. How is COVID-19 pandemic impacting mental health of children and adolescents? Int J Disaster Risk Reduct. 2020 Dec;51:101845. doi: 10.1016/j.ijdrr.2020.101845. Epub 2020 Sep 10. PMID: 32929399; PMCID: PMC7481176. | S |
| Martinelli A, Ruggeri M. The impact of COVID-19 on patients of Italian mental health supported accommodation services. Soc Psychiatry Psychiatr Epidemiol. 2020 Oct;55(10):1395-1396. doi: 10.1007/s00127-020-01897-7. Epub 2020 Jul 25. PMID: 32712677; PMCID: PMC7381855. | C |
| Martínez-Martí ML, Theirs CI, Pascual D, Corradi G. Character Strengths Predict an Increase in Mental Health and Subjective Well-Being Over a One-Month Period During the COVID-19 Pandemic Lockdown. Front Psychol. 2020 Oct 21;11:584567. doi: 10.3389/fpsyg.2020.584567. PMID: 33192913; PMCID: PMC7609545. | C |
| Matsubayashi T, Sekijima K, Ueda M. Government spending, recession, and suicide: evidence from Japan. BMC Public Health. 2020 Feb 21;20(1):243. doi: 10.1186/s12889-020-8264-1. PMID: 32079525; PMCID: PMC7033906. | E/I |
| Mattei G, De Vogli R, Ferrari S, Pingani L, Rigatelli M, Galeazzi GM. Impact of the economic crisis on health-related behaviors in Italy. Int J Soc Psychiatry. 2017 Nov;63(7):649-656. doi: 10.1177/0020764017726097. Epub 2017 Aug 23. PMID: 28831854. | S |
| Matvienko-Sikar K, Meedya S, Ravaldi C. Perinatal mental health during the COVID-19 pandemic. Women Birth. 2020 Jul;33(4):309-310. doi: 10.1016/j.wombi.2020.04.006. Epub 2020 May 7. PMID: 32418652; PMCID: PMC7203052. | C |
| Maunder R. The experience of the 2003 SARS outbreak as a traumatic stress among frontline healthcare workers in Toronto: lessons learned. Philos Trans R Soc Lond B Biol Sci. 2004 Jul 29;359(1447):1117-25. doi: 10.1098/rstb.2004.1483. PMID: 15306398; PMCID: PMC1693388. | C |
| Mazhar K, Hussain S, Ullah R, Raza H, Aamir A, Asif A, Memon FS, Haider J, Anas M. Mental Health Crisis in Pregnant Women during Current COVID-19 Pandemic. Psychiatr Danub. 2020 Autumn;32(3-4):598-599. PMID: 33373992. | P |
| Mazza MG, De Lorenzo R, Conte C, Poletti S, Vai B, Bollettini I, Melloni EMT, Furlan R, Ciceri F, Rovere-Querini P; COVID-19 BioB Outpatient Clinic Study group, Benedetti F. Anxiety and depression in COVID-19 survivors: Role of inflammatory and clinical predictors. Brain Behav Immun. 2020 Oct;89:594-600. doi: 10.1016/j.bbi.2020.07.037. Epub 2020 Jul 30. PMID: 32738287; PMCID: PMC7390748. | C |
| Mc Gee U, Sanders E. Letter to the editor regarding the effect of isolation on athletes' mental health during the COVID-19 pandemic. Phys Sportsmed. 2020 Nov 17:1. doi: 10.1080/00913847.2020.1845582. Epub ahead of print. PMID: 33148087. | S |
| McInerney M, Mellor JM. Recessions and seniors' health, health behaviors, and healthcare use: analysis of the Medicare Current Beneficiary Survey. J Health Econ. 2012 Sep;31(5):744-51. doi: 10.1016/j.jhealeco.2012.06.002. Epub 2012 Jul 7. PMID: 22898452. | E/I |
| McLaughlin KA, Nandi A, Keyes KM, Uddin M, Aiello AE, Galea S, Koenen KC. Home foreclosure and risk of psychiatric morbidity during the recent financial crisis. Psychol Med. 2012 Jul;42(7):1441-8. doi: 10.1017/S0033291711002613. Epub 2011 Nov 21. PMID: 22099861; PMCID: PMC3438142. | E/I |
| Mehra A, Rani S, Sahoo S, Parveen S, Singh AP, Chakrabarti S, Grover S. A crisis for elderly with mental disorders: Relapse of symptoms due to heightened anxiety due to COVID-19. Asian J Psychiatr. 2020 Jun;51:102114. doi: 10.1016/j.ajp.2020.102114. Epub 2020 Apr 18. PMID: 32334406; PMCID: PMC7166027. | C |
| Mehra A, Sahoo S, Suri V, Malhotra P, Yaddanapudi N, Puri GD, Grover S. Why involvement of mental health professionals and screening for past mental illness is important in persons with COVID-19 infection: A case report. Asian J Psychiatr. 2020 Dec;54:102294. doi: 10.1016/j.ajp.2020.102294. Epub 2020 Jul 6. PMID: 32663797; PMCID: PMC7832217. | S |
| Mejía ST, Settersten RA Jr, Odden MC, Hooker K. Responses to Financial Loss During the Great Recession: An Examination of Sense of Control in Late Midlife. J Gerontol B Psychol Sci Soc Sci. 2016 Jul;71(4):734-44. doi: 10.1093/geronb/gbv054. Epub 2015 Aug 25. PMID: 26307482; PMCID: PMC5013892. | O |
| Mental Health Effects of COVID-19. Am J Nurs. 2020 Nov;120(11):15. doi: 10.1097/01.NAJ.0000721880.79285.04. PMID: 33105208. | P |
| Meyer, J. ;McDowell, C. ;Lansing, J. ;Brower, C. ;Smith, L. ;Tully, M. ;Herring, M. Changes in Physical Activity and Sedentary Behavior in Response to COVID-19 and Their Associations with Mental Health in 3052 US Adults (vol 17, 6469, 2020). International Journal of Environmental Research and Public Health. 2020. doi 10.3390/ijerph17196949 | C |
| Miller JJ, Cooley ME, Mihalec-Adkins BP. Examining the Impact of COVID-19 on Parental Stress: A Study of Foster Parents. Child Adolesc Social Work J. 2020 Nov 20:1-10. doi: 10.1007/s10560-020-00725-w. Epub ahead of print. PMID: 33235406; PMCID: PMC7677097. | S |
| Mitra S, Kavoor AR. The missing discourse on mental health of psychiatrists during COVID-19. Aust N Z J Psychiatry. 2021 Mar;55(3):325-326. doi: 10.1177/0004867420957084. Epub 2020 Sep 8. PMID: 32900212. | C |
| Modrek S, Cullen MR. Job insecurity during recessions: effects on survivors' work stress. BMC Public Health. 2013 Oct 6;13:929. doi: 10.1186/1471-2458-13-929. PMID: 24093476; PMCID: PMC3853134. | S |
| Mohring, K. ;Naumann, E. ;Reifenscheid, M. ;Wenz, A. ;Rettig, T. ;Krieger, U. ;Friedel, S. ;Finkel, M. ;Cornesse, C. ;Blom, A. G. The COVID-19 pandemic and subjective well-being: longitudinal evidence on satisfaction with work and family. European Societies. doi 10.1080/14616696.2020.1833066 | O |
| Moncho J, Pereyra-Zamora P, Tamayo-Fonseca N, Giron M, Gómez-Beneyto M, Nolasco A. Is recession bad for your mental health? The answer could be complex: evidence from the 2008 crisis in Spain. BMC Med Res Methodol. 2018 Jul 13;18(1):78. doi: 10.1186/s12874-018-0538-2. PMID: 30001696; PMCID: PMC6044068. | S |
| Monteith LL, Holliday R, Brown TL, Brenner LA, Mohatt NV. Preventing Suicide in Rural Communities During the COVID-19 Pandemic. J Rural Health. 2021 Jan;37(1):179-184. doi: 10.1111/jrh.12448. Epub 2020 May 30. PMID: 32282968; PMCID: PMC7262063. | C |
| Moser A, Carlander M, Wieser S, Hämmig O, Puhan MA, Höglinger M. The COVID-19 Social Monitor longitudinal online panel: Real-time monitoring of social and public health consequences of the COVID-19 emergency in Switzerland. PLoS One. 2020 Nov 11;15(11):e0242129. doi: 10.1371/journal.pone.0242129. PMID: 33175906; PMCID: PMC7657546. | C |
| Moser DA, Glaus J, Frangou S, Schechter DS. Years of life lost due to the psychosocial consequences of COVID-19 mitigation strategies based on Swiss data. Eur Psychiatry. 2020 May 29;63(1):e58. doi: 10.1192/j.eurpsy.2020.56. PMID: 32466820; PMCID: PMC7303469. | S |
| Mourouvaye M, Bottemanne H, Bonny G, Fourcade L, Angoulvant F, Cohen JF, Ouss L. Association between suicide behaviours in children and adolescents and the COVID-19 lockdown in Paris, France: a retrospective observational study. Arch Dis Child. 2020 Dec 22:archdischild-2020-320628. doi: 10.1136/archdischild-2020-320628. Epub ahead of print. PMID: 33355154. | C |
| Mousavi SB. Coronavirus disease 2019 pandemic: Do not forget patients with severe mental illness. Int J Soc Psychiatry. 2020 Jul 7:20764020939982. doi: 10.1177/0020764020939982. Epub ahead of print. PMID: 32633183. | P |
| Mousteri V, Daly M, Delaney L, Tynelius P, Rasmussen F. Adolescent mental health and unemployment over the lifespan: Population evidence from Sweden. Soc Sci Med. 2019 Feb;222:305-314. doi: 10.1016/j.socscimed.2018.12.030. Epub 2019 Jan 8. PMID: 30677644. | E/I |
| Mukhtar S. Psychological health during the coronavirus disease 2019 pandemic outbreak. Int J Soc Psychiatry. 2020 Aug;66(5):512-516. doi: 10.1177/0020764020925835. Epub 2020 May 21. PMID: 32434402; PMCID: PMC7405632. | C |
| Muller AE, Hafstad EV, Himmels JPW, Smedslund G, Flottorp S, Stensland SØ, Stroobants S, Van de Velde S, Vist GE. The mental health impact of the covid-19 pandemic on healthcare workers, and interventions to help them: A rapid systematic review. Psychiatry Res. 2020 Nov;293:113441. doi: 10.1016/j.psychres.2020.113441. Epub 2020 Sep 1. PMID: 32898840; PMCID: PMC7462563. | C |
| Mumtaz M. COVID-19 and mental health challenges in Pakistan. Int J Soc Psychiatry. 2021 May;67(3):303-304. doi: 10.1177/0020764020954487. Epub 2020 Sep 9. PMID: 32900252. | P |
| Munasinghe S, Sperandei S, Freebairn L, Conroy E, Jani H, Marjanovic S, Page A. The Impact of Physical Distancing Policies During the COVID-19 Pandemic on Health and Well-Being Among Australian Adolescents. J Adolesc Health. 2020 Nov;67(5):653-661. doi: 10.1016/j.jadohealth.2020.08.008. PMID: 33099413; PMCID: PMC7577185. | C |
| Mutlu E, Anıl Yağcıoğlu AE. Relapse in patients with serious mental disorders during the COVID-19 outbreak: a retrospective chart review from a community mental health center. Eur Arch Psychiatry Clin Neurosci. 2021 Mar;271(2):381-383. doi: 10.1007/s00406-020-01203-1. Epub 2020 Oct 26. PMID: 33104904; PMCID: PMC7587161. | C |
| Mutz, M. Forced adaptations of sporting behaviours during the Covid-19 pandemic and their effects on subjective well-being. European Societies. Doi 10.1080/14616696.2020.1821077 | S |
| Nagae M, Sakamoto M, Horikawa E. Work-sharing and male employees' mental health during an economic recession. Occup Med (Lond). 2017 Dec 2;67(8):648-651. doi: 10.1093/occmed/kqx135. PMID: 29016936. | C |
| Nearchou F, Flinn C, Niland R, Subramaniam SS, Hennessy E. Exploring the Impact of COVID-19 on Mental Health Outcomes in Children and Adolescents: A Systematic Review. Int J Environ Res Public Health. 2020 Nov 16;17(22):8479. doi: 10.3390/ijerph17228479. PMID: 33207689; PMCID: PMC7698263. | S |
| Nelson B, Kaminsky DB. COVID-19's crushing mental health toll on health care workers: Beyond its devastating physical effects, the pandemic has unleashed a mental health crisis marked by anxiety, depression, posttraumatic stress disorder, and even suicide. Here, in part 1 of a 2-part series, we examine the growing effort to identify and alleviate the fallout for health care workers. Cancer Cytopathol. 2020 Sep;128(9):597-598. doi: 10.1002/cncy.22347. PMID: 32885911. | P |
| Nelson B, Kaminsky DB. COVID-19's multipronged attack on mental health: Months of uncertainty and threats to health, social, and financial security have contributed to intense anxiety, depression, posttraumatic stress disorder, and even suicide. Here, in part 2 of a 2-part series, we investigate the pandemic's growing mental health toll on the general public and efforts to address it. Cancer Cytopathol. 2020 Oct;128(10):679-680. doi: 10.1002/cncy.22364. PMID: 33006815; PMCID: PMC7537285. | Other duplicate |
| Nelson NA, Bergeman CS. Daily Stress Processes in a Pandemic: The Effects of Worry, Age, and Affect. Gerontologist. 2021 Feb 23;61(2):196-204. doi: 10.1093/geront/gnaa187. PMID: 33186445; PMCID: PMC7717331. | C |
| Nelson, B. ;Kaminsky, D. B. COVID-19's Multipronged Attack on Mental Health. Cancer Cytopathology. 2020. doi 10.1002/cncy.22364 | P |
| Nodoushan RJ, Alimoradi H, Nazari M. Spiritual Health and Stress in Pregnant Women During the Covid-19 Pandemic. SN Compr Clin Med. 2020 Oct 16:1-7. doi: 10.1007/s42399-020-00582-9. Epub ahead of print. PMID: 33083694; PMCID: PMC7561430. | C |
| Nordt C, Warnke I, Seifritz E, Kawohl W. Modelling suicide and unemployment: a longitudinal analysis covering 63 countries, 2000-11. Lancet Psychiatry. 2015 Mar;2(3):239-45. doi: 10.1016/S2215-0366(14)00118-7. Epub 2015 Feb 25. PMID: 26359902. | E/I |
| Norström T, Grönqvist H. The Great Recession, unemployment and suicide. J Epidemiol Community Health. 2015 Feb;69(2):110-6. doi: 10.1136/jech-2014-204602. Epub 2014 Oct 22. PMID: 25339416; PMCID: PMC4316842. | E/I |
| Nour S, Labonté R, Bancej C. Impact of the 2008 global financial crisis on the health of Canadians: repeated cross-sectional analysis of the Canadian Community Health Survey, 2007-2013. J Epidemiol Community Health. 2017 Apr;71(4):336-343. doi: 10.1136/jech-2016-207661. Epub 2016 Nov 9. PMID: 27831480. | S |
| O'Connor RC, Wetherall K, Cleare S, McClelland H, Melson AJ, Niedzwiedz CL, O'Carroll RE, O'Connor DB, Platt S, Scowcroft E, Watson B, Zortea T, Ferguson E, Robb KA. Mental health and well-being during the COVID-19 pandemic: longitudinal analyses of adults in the UK COVID-19 Mental Health & Wellbeing study. Br J Psychiatry. 2020 Oct 21:1-8. doi: 10.1192/bjp.2020.212. Epub ahead of print. PMID: 33081860; PMCID: PMC7684009. | C |
| Omiya Y, Tokuno S. How much of an impact did COVID-19 self-isolation measures have on mental health? Asian J Psychiatr. 2020 Dec;54:102445. doi: 10.1016/j.ajp.2020.102445. Epub 2020 Oct 14. PMID: 33271724; PMCID: PMC7556281. | S |
| Oppo V, Serra G, Fenu G, Murgia D, Ricciardi L, Melis M, Morgante F, Cossu G. Parkinson's Disease Symptoms Have a Distinct Impact on Caregivers' and Patients' Stress: A Study Assessing the Consequences of the COVID-19 Lockdown. Mov Disord Clin Pract. 2020 Sep 16;7(7):865-867. doi: 10.1002/mdc3.13030. PMID: 33043088; PMCID: PMC7533970. | C |
| O'Reilly A, Tibbs M, Booth A, Doyle E, McKeague B, Moore J. A rapid review investigating the potential impact of a pandemic on the mental health of young people aged 12-25 years. Ir J Psychol Med. 2020 Sep 11:1-16. doi: 10.1017/ipm.2020.106. Epub ahead of print. PMID: 32912358; PMCID: PMC7711353. | S |
| Ornell F, Halpern SC, Kessler FHP, Narvaez JCM. The impact of the COVID-19 pandemic on the mental health of healthcare professionals. Cad Saude Publica. 2020 Apr 30;36(4):e00063520. doi: 10.1590/0102-311X00063520. PMID: 32374807. | C |
| Ostamo A, Lönnqvist J. Attempted suicide rates and trends during a period of severe economic recession in Helsinki, 1989-1997. Soc Psychiatry Psychiatr Epidemiol. 2001 Jul;36(7):354-60. doi: 10.1007/s001270170041. PMID: 11606005. | E/I |
| Palacio A CA. Mental Health in Times of Pandemic. Rev Colomb Psiquiatr (Engl Ed). 2020 Jul-Sep;49(3):135. English, Spanish. doi: 10.1016/j.rcp.2020.07.001. Epub 2020 Aug 14. PMID: 32888655. | P |
| Pan R, Zhang L, Pan J. The Anxiety Status of Chinese Medical Workers During the Epidemic of COVID-19: A Meta-Analysis. Psychiatry Investig. 2020 May;17(5):475-480. doi: 10.30773/pi.2020.0127. Epub 2020 May 15. PMID: 32403209; PMCID: PMC7265026. | C |
| Pandya P. Effect on mental health issues during the COVID-19 pandemic. Br J Gen Pract. 2020 Jul 30;70(697):382. doi: 10.3399/bjgp20X711857. PMID: 32732196; PMCID: PMC7384829. | S |
| Pellerin N, Raufaste E. Psychological Resources Protect Well-Being During the COVID-19 Pandemic: A Longitudinal Study During the French Lockdown. Front Psychol. 2020 Dec 4;11:590276. doi: 10.3389/fpsyg.2020.590276. PMID: 33424709; PMCID: PMC7793808. | C |
| Peppou LE, Economou M, Skali T, Papageorgiou C. From economic crisis to the COVID-19 pandemic crisis: evidence from a mental health helpline in Greece. Eur Arch Psychiatry Clin Neurosci. 2021 Mar;271(2):407-409. doi: 10.1007/s00406-020-01165-4. Epub 2020 Jul 14. PMID: 32666279; PMCID: PMC7358697. | S |
| Pereira-Sanchez V, Adiukwu F, El Hayek S, Bytyçi DG, Gonzalez-Diaz JM, Kundadak GK, Larnaout A, Nofal M, Orsolini L, Ramalho R, Ransing R, Shalbafan M, Soler-Vidal J, Syarif Z, Teixeira ALS, da Costa MP. COVID-19 effect on mental health: patients and workforce. Lancet Psychiatry. 2020 Jun;7(6):e29-e30. doi: 10.1016/S2215-0366(20)30153-X. Epub 2020 May 20. PMID: 32445691; PMCID: PMC7239628. | C |
| Peretti-Watel P, Alleaume C, Léger D, Beck F, Verger P; COCONEL Group. Anxiety, depression and sleep problems: a second wave of COVID-19. Gen Psychiatr. 2020 Sep 22;33(5):e100299. doi: 10.1136/gpsych-2020-100299. PMID: 33083692; PMCID: PMC7513629. | C |
| Pfefferbaum B, North CS. Mental Health and the Covid-19 Pandemic. N Engl J Med. 2020 Aug 6;383(6):510-512. doi: 10.1056/NEJMp2008017. Epub 2020 Apr 13. PMID: 32283003. | C |
| Pfoertner TK, Rathmann K, Elgar FJ, de Looze M, Hofmann F, Ottova-Jordan V, Ravens-Sieberer U, Bosakova L, Currie C, Richter M. Adolescents' psychological health complaints and the economic recession in late 2007: a multilevel study in 31 countries. Eur J Public Health. 2014 Dec;24(6):961-7. doi: 10.1093/eurpub/cku056. Epub 2014 May 23. PMID: 24860027. | C |
| Pınar Senkalfa B, Sismanlar Eyuboglu T, Aslan AT, Ramaslı Gursoy T, Soysal AS, Yapar D, İlhan MN. Effect of the COVID-19 pandemic on anxiety among children with cystic fibrosis and their mothers. Pediatr Pulmonol. 2020 Aug;55(8):2128-2134. doi: 10.1002/ppul.24900. Epub 2020 Jun 26. PMID: 32530552; PMCID: PMC7307121. | C |
| Planchuelo-Gómez Á, Odriozola-González P, Irurtia MJ, de Luis-García R. Longitudinal evaluation of the psychological impact of the COVID-19 crisis in Spain. J Affect Disord. 2020 Dec 1;277:842-849. doi: 10.1016/j.jad.2020.09.018. Epub 2020 Sep 7. PMID: 33065825; PMCID: PMC7476580. | C |
| Polsek, D. Psychiatry of Pandemics: a Mental Health Response to Infection Outbreak. Croatian Medical Journal. 2020. doi  10.3325/cmj.2020.61.306 | C |
| Poudel K, Subedi P. Impact of COVID-19 pandemic on socioeconomic and mental health aspects in Nepal. Int J Soc Psychiatry. 2020 Dec;66(8):748-755. doi: 10.1177/0020764020942247. Epub 2020 Jul 10. PMID: 32650687; PMCID: PMC7443960. | S |
| Preti E, Di Mattei V, Perego G, Ferrari F, Mazzetti M, Taranto P, Di Pierro R, Madeddu F, Calati R. The Psychological Impact of Epidemic and Pandemic Outbreaks on Healthcare Workers: Rapid Review of the Evidence. Curr Psychiatry Rep. 2020 Jul 10;22(8):43. doi: 10.1007/s11920-020-01166-z. PMID: 32651717; PMCID: PMC7350408. | C |
| Probst T, Budimir S, Pieh C. Depression in and after COVID-19 lockdown in Austria and the role of stress and loneliness in lockdown: A longitudinal study. J Affect Disord. 2020 Dec 1;277:962-963. doi: 10.1016/j.jad.2020.09.047. Epub 2020 Sep 13. PMID: 33065839; PMCID: PMC7487145. | C |
| Purtle J. COVID-19 and mental health equity in the United States. Soc Psychiatry Psychiatr Epidemiol. 2020 Aug;55(8):969-971. doi: 10.1007/s00127-020-01896-8. Epub 2020 Jun 17. PMID: 32556376; PMCID: PMC7298157. | P |
| Rahimi, R. Dolatabadi, Z. Moeindarbary, S. Behzadfar, S. Ghasemi, N. F. Tafrishi, R. Kamali, M. A Systematic Review of the Prevalence of Mental Health Disorders in Pregnant Women during the COVID-19 Pandemic. International Journal of Pediatrics-Mashhad. 2020. doi 10.22038/ijp.2020.52315.4155 | C |
| Raihan, M. M. H. Mental health consequences of COVID-19 pandemic on adult population: a systematic review. Mental Health Review Journal. Doi  10.1108/mhrj-07-2020-0044 | C |
| Rathmann K, Pförtner TK, Osorio AM, Hurrelmann K, Elgar FJ, Bosakova L, Richter M. Adolescents' psychological health during the economic recession: does public spending buffer health inequalities among young people? BMC Public Health. 2016 Aug 24;16(1):860. doi: 10.1186/s12889-016-3551-6. PMID: 27553033; PMCID: PMC4995668. | E/I |
| Real E, Jover L, Verdaguer R, Griera A, Segalàs C, Alonso P, Contreras F, Arteman A, Menchón JM. Factors Associated with Long-Term Sickness Absence Due to Mental Disorders: A Cohort Study of 7.112 Patients during the Spanish Economic Crisis. PLoS One. 2016 Jan 5;11(1):e0146382. doi: 10.1371/journal.pone.0146382. PMID: 26730603; PMCID: PMC4701450. | E/I |
| Reeves A, Stuckler D, McKee M, Gunnell D, Chang SS, Basu S. Increase in state suicide rates in the USA during economic recession. Lancet. 2012 Nov 24;380(9856):1813-4. doi: 10.1016/S0140-6736(12)61910-2. Epub 2012 Nov 6. PMID: 23141814. | S |
| Reile R, Helakorpi S, Klumbiene J, Tekkel M, Leinsalu M. The recent economic recession and self-rated health in Estonia, Lithuania and Finland: a comparative cross-sectional study in 2004-2010. J Epidemiol Community Health. 2014 Nov;68(11):1072-9. doi: 10.1136/jech-2014-204196. Epub 2014 Jul 16. PMID: 25031451. | O |
| Ren X, Huang W, Pan H, Huang T, Wang X, Ma Y. Mental Health During the Covid-19 Outbreak in China: a Meta-Analysis. Psychiatr Q. 2020 Dec;91(4):1033-1045. doi: 10.1007/s11126-020-09796-5. PMID: 32642822; PMCID: PMC7343383. | C |
| Ren Y, Zhou Y, Qian W, Li Z, Liu Z, Wang R, Qi L, Yang J, Song X, Zeng L, Zhang X. Letter to the Editor "A longitudinal study on the mental health of general population during the COVID-19 epidemic in China". Brain Behav Immun. 2020 Jul;87:132-133. doi: 10.1016/j.bbi.2020.05.004. Epub 2020 May 6. PMID: 32387510; PMCID: PMC7201232. | S |
| Rezende Machado de Sousa L, Saint Ville A, Maria Segall-Corrêa A, Melgar-Quiñonez H. Health inequalities and well-being in times of financial and political crisis in Brazil, a case study. Glob Public Health. 2019 Dec;14(12):1815-1828. doi: 10.1080/17441692.2019.1616800. Epub 2019 May 15. PMID: 31088204. | E/I |
| Rihmer Z, Kapitany B, Gonda X, Dome P. Suicide, recession, and unemployment. Lancet. 2013 Mar 2;381(9868):722-3. doi: 10.1016/S0140-6736(13)60575-9. PMID: 23472913. | S |
| Robinson E, Daly M. Explaining the rise and fall of psychological distress during the COVID-19 crisis in the United States: Longitudinal evidence from the Understanding America Study. Br J Health Psychol. 2021 May;26(2):570-587. doi: 10.1111/bjhp.12493. Epub 2020 Dec 5. PMID: 33278066. | C |
| Roca, M. ;Vicens, C. ;Gili, M. COVID-19: PSYCHOLOGICAL EFFECTS ON HEALTHCARE WORKERS Covid-19 and the future of mental health in primary care. Bmj-British Medical Journal. 2020. doi 10.1136/bmj.m2520 | C |
| Rodrigues DFS, Nunes C. Inpatient Profile of Patients with Major Depression in Portuguese National Health System Hospitals, in 2008 and 2013: Variation in a Time of Economic Crisis. Community Ment Health J. 2018 Feb;54(2):224-235. doi: 10.1007/s10597-017-0144-5. Epub 2017 May 4. PMID: 28474141. | S |
| Rohde N, Tang KK, Osberg L, Rao P. The effect of economic insecurity on mental health: Recent evidence from Australian panel data. Soc Sci Med. 2016 Feb;151:250-8. doi: 10.1016/j.socscimed.2015.12.014. Epub 2016 Jan 4. PMID: 26826683. | C |
| Rojas Y, Stenberg SÅ. Evictions and suicide: a follow-up study of almost 22,000 Swedish households in the wake of the global financial crisis. J Epidemiol Community Health. 2016 Apr;70(4):409-13. doi: 10.1136/jech-2015-206419. Epub 2015 Nov 4. PMID: 26537566; PMCID: PMC4819654. | E/I |
| Ruggeri, M. ;Tomassi, S. The impact of economic crisis on mental and physical health in Italy. Die Psychiatrie: Grundlagen & Perspektiven. 2017. | Other duplicate |
| Said RM, El-Shafei DA. Occupational stress, job satisfaction, and intent to leave: nurses working on front lines during COVID-19 pandemic in Zagazig City, Egypt. Environ Sci Pollut Res Int. 2021 Feb;28(7):8791-8801. doi: 10.1007/s11356-020-11235-8. Epub 2020 Oct 17. PMID: 33067794; PMCID: PMC7567651. | S |
| Salmanian M, Salehi M, Hooshyari Z. Global Prevalence of Posttraumatic Stress Disorder (PTSD) during and after Coronavirus Pandemic: A Study Protocol for a Systematic Review and Meta-Analysis. Iran J Psychiatry. 2020 Jul;15(3):252-255. doi: 10.18502/ijps.v15i3.3819. PMID: 33193775; PMCID: PMC7603595. | S |
| Santana, P. ;Loureiro, A. ;Almendra, R. ;Nunes, C. Determinants of mental health in Lisbon Region in a period of economic crisis. A multi-level study.  European Journal of Public Health. 2016. | S |
| Sarwar MAA, Sarwar H. The Impact of COVID-19 on the Mental Health of Healthcare Professionals. J Coll Physicians Surg Pak. 2020 Jun;30(6):83. doi: 10.29271/jcpsp.2020.Supp1.S83. PMID: 32723465. | C |
| Sasaki N, Kuroda R, Tsuno K, Imamura K, Kawakami N. Deterioration in Mental Health Under Repeated COVID-19 Outbreaks Greatest in the Less Educated: A Cohort Study of Japanese Employees. J Epidemiol. 2021 Jan 5;31(1):93-96. doi: 10.2188/jea.JE20200499. Epub 2020 Nov 25. PMID: 33162424; PMCID: PMC7738638. | E/I |
| Sasaki, N. ;Kuroda, R. ;Tsuno, K. ;Imamura, K. ;Kawakami, N. Mental health deteriorated mostly in the less educated under repeated COVID-19 outbreaks: A cohort study of Japanese employees. J Epidemiol. 2020. doi 10.2188/jea.JE20200499 | C |
| Sciberras E, Patel P, Stokes MA, Coghill D, Middeldorp CM, Bellgrove MA, Becker SP, Efron D, Stringaris A, Faraone SV, Bellows ST, Quach J, Banaschewski T, McGillivray J, Hutchinson D, Silk TJ, Melvin G, Wood AG, Jackson A, Loram G, Engel L, Montgomery A, Westrupp E. Physical Health, Media Use, and Mental Health in Children and Adolescents With ADHD During the COVID-19 Pandemic in Australia. J Atten Disord. 2020 Dec 17:1087054720978549. doi: 10.1177/1087054720978549. Epub ahead of print. PMID: 33331195. | C |
| Seghi F, Barbini B, Franchini L, Colombo C. The challenge of mental health during Covid-19 outbreak: experience from metropolitan area of Milan. Eur Arch Psychiatry Clin Neurosci. 2021 Mar;271(2):401-402. doi: 10.1007/s00406-020-01154-7. Epub 2020 Jun 20. PMID: 32564126; PMCID: PMC7305475. | C |
| Senczyszyn A, Lion KM, Szcześniak D, Trypka E, Mazurek J, Ciułkowicz M, Maćkowiak M, Duda-Sikuła M, Wallner R, Rymaszewska J. Mental Health Impact of SARS-COV-2 Pandemic on Long-Term Care Facility Personnel in Poland. J Am Med Dir Assoc. 2020 Nov;21(11):1576-1577. doi: 10.1016/j.jamda.2020.09.020. Epub 2020 Sep 19. PMID: 33138941; PMCID: PMC7501840. | C |
| Şenışık S, Denerel N, Köyağasıoğlu O, Tunç S. The effect of isolation on athletes' mental health during the COVID-19 pandemic. Phys Sportsmed. 2021 May;49(2):187-193. doi: 10.1080/00913847.2020.1807297. Epub 2020 Sep 1. PMID: 32762510. | C |
| Serafim AP, Gonçalves PD, Rocca CC, Lotufo Neto F. The impact of COVID-19 on Brazilian mental health through vicarious traumatization. Braz J Psychiatry. 2020 Aug;42(4):450. doi: 10.1590/1516-4446-2020-0999. Epub 2020 May 11. PMID: 32401867; PMCID: PMC7430376. | S |
| Serafini G, Parmigiani B, Amerio A, Aguglia A, Sher L, Amore M. The psychological impact of COVID-19 on the mental health in the general population. QJM. 2020 Jun 22;113(8):531–7. doi: 10.1093/qjmed/hcaa201. Epub ahead of print. PMID: 32569360; PMCID: PMC7337855. | C |
| Serrano-Ripoll MJ, Meneses-Echavez JF, Ricci-Cabello I, Fraile-Navarro D, Fiol-deRoque MA, Pastor-Moreno G, Castro A, Ruiz-Pérez I, Zamanillo Campos R, Gonçalves-Bradley DC. Impact of viral epidemic outbreaks on mental health of healthcare workers: a rapid systematic review and meta-analysis. J Affect Disord. 2020 Dec 1;277:347-357. doi: 10.1016/j.jad.2020.08.034. Epub 2020 Aug 23. PMID: 32861835; PMCID: PMC7443314. | S |
| Shi J, Gao Y, Zhao L, Li Y, Yan M, Niu MM, Chen Y, Song Z, Zhang R, Zhang L, Tian J. Prevalence of delirium, depression, anxiety, and post-traumatic stress disorder among COVID-19 patients: protocol for a living systematic review. Syst Rev. 2020 Nov 6;9(1):258. doi: 10.1186/s13643-020-01507-2. PMID: 33158456; PMCID: PMC7646715. | C |
| Shigemura J, Ursano RJ, Morganstein JC, Kurosawa M, Benedek DM. Public responses to the novel 2019 coronavirus (2019-nCoV) in Japan: Mental health consequences and target populations. Psychiatry Clin Neurosci. 2020 Apr;74(4):281-282. doi: 10.1111/pcn.12988. Epub 2020 Feb 23. PMID: 32034840; PMCID: PMC7168047. | C |
| Shim RS. Mental Health Inequities in the Context of COVID-19. JAMA Netw Open. 2020 Sep 1;3(9):e2020104. doi: 10.1001/jamanetworkopen.2020.20104. PMID: 32876681. | P |
| Sicras-Mainar A, Navarro-Artieda R. Use of antidepressants in the treatment of major depressive disorder in primary care during a period of economic crisis. Neuropsychiatr Dis Treat. 2015 Dec 30;12:29-40. doi: 10.2147/NDT.S91227. PMID: 26766910; PMCID: PMC4699544. | C |
| Silva M, Antunes A, Azeredo-Lopes S, Cardoso G, Xavier M, Saraceno B, Caldas-de-Almeida JM. How did the use of psychotropic drugs change during the Great Recession in Portugal? A follow-up to the National Mental Health Survey. BMC Psychiatry. 2020 May 11;20(1):215. doi: 10.1186/s12888-020-02620-1. PMID: 32393219; PMCID: PMC7216710. | C |
| Silva, M. ;Antunes, A. ;Frasquilho, D. ;Cardoso, G. ;Caldas-de-Almeida, J. M. The impact of the economic crisis on the use of psychotropic medication in Portugal: Preliminary results of the national mental health survey follow-up. European Psychiatry. 2017. doi 10.1016/j.eurpsy.2017.01.866 | S |
| Singh OP. Mental health of migrant laborers in COVID-19 pandemic and lockdown: Challenges ahead. Indian J Psychiatry. 2020 May-Jun;62(3):233-234. doi: 10.4103/psychiatry.IndianJPsychiatry_422_20. Epub 2020 May 15. PMID: 32773863; PMCID: PMC7368439. | P |
| Skoog I. COVID-19 and mental health among older people in Sweden. Int Psychogeriatr. 2020 Oct;32(10):1173-1175. doi: 10.1017/S104161022000143X. Epub 2020 Jul 8. PMID: 32635950; PMCID: PMC7403743. | P |
| Smith CA. Covid-19: healthcare students face unique mental health challenges. BMJ. 2020 Jun 29;369:m2491. doi: 10.1136/bmj.m2491. PMID: 32601100. | P |
| Smith K, Bhui K, Cipriani A. COVID-19, mental health and ethnic minorities. Evid Based Ment Health. 2020 Aug;23(3):89-90. doi: 10.1136/ebmental-2020-300174. Epub 2020 Jul 17. PMID: 32680834; PMCID: PMC7418618. | S |
| Smith, C. A. COVID-19: PSYCHOLOGICAL EFFECTS ON HEALTHCARE WORKERS Covid-19: healthcare students face unique mental health challenges. Bmj-British Medical Journal. 2020. doi 10.1136/bmj.m2491 | S |
| Soldevila-Domenech N, Forcano L, Boronat A, Lorenzo T, Piera I, Puig-Pijoan A, Mateus J, González de Echevarri Gómez JM, Knezevic I, Soteras A, Fauria K, Pizarro N, Molinuevo JL, de la Torre R; PENSA Study Group. From IMIM:; From BBRC:. Effects of COVID-19 Home Confinement on Mental Health in Individuals with Increased Risk of Alzheimer's Disease. J Alzheimers Dis. 2021;79(3):1015-1021. doi: 10.3233/JAD-201408. PMID: 33386809; PMCID: PMC7990405. | E/I |
| Somarriba Arechavala, Noelia ;Zarzosa Espina, Pilar ;Pena Trapero, Bernardo. The Economic Crisis and its Effects on the Quality of Life in the European Union. Social Indicators Research. 2015. doi http://dx.doi.org/10.1007/s11205-014-0595-9 | C |
| Spoorthy MS, Pratapa SK, Mahant S. Mental health problems faced by healthcare workers due to the COVID-19 pandemic-A review. Asian J Psychiatr. 2020 Jun;51:102119. doi: 10.1016/j.ajp.2020.102119. Epub 2020 Apr 22. PMID: 32339895; PMCID: PMC7175897. | C |
| Stanzani, Sandro. Economic crisis and well-being in Italy. Italian Sociological Review.2012. | C |
| Staples L, Nielssen O, Kayrouz R, Cross S, Karin E, Ryan K, Dear B, Titov N. Rapid report 2: Symptoms of anxiety and depression during the first 12 weeks of the Coronavirus (COVID-19) pandemic in Australia. Internet Interv. 2020 Dec;22:100351. doi: 10.1016/j.invent.2020.100351. Epub 2020 Oct 22. PMID: 33110762; PMCID: PMC7580521. | C |
| Starace F, Mungai F, Sarti E, Addabbo T. Being hit twice: The psychological consequences of the economic crisis and an earthquake. Int J Soc Psychiatry. 2016 Jun;62(4):345-9. doi: 10.1177/0020764016633490. Epub 2016 Feb 29. PMID: 26929188. | E/I |
| Stuijfzand S, Deforges C, Sandoz V, Sajin CT, Jaques C, Elmers J, Horsch A. Psychological impact of an epidemic/pandemic on the mental health of healthcare professionals: a rapid review. BMC Public Health. 2020 Aug 12;20(1):1230. doi: 10.1186/s12889-020-09322-z. PMID: 32787815; PMCID: PMC7422454. | C |
| Styra R, Hawryluck L, Robinson S, Kasapinovic S, Fones C, Gold WL. Impact on health care workers employed in high-risk areas during the Toronto SARS outbreak. J Psychosom Res. 2008 Feb;64(2):177-83. doi: 10.1016/j.jpsychores.2007.07.015. PMID: 18222131; PMCID: PMC7094601. | C |
| Su TP, Lien TC, Yang CY, Su YL, Wang JH, Tsai SL, Yin JC. Prevalence of psychiatric morbidity and psychological adaptation of the nurses in a structured SARS caring unit during outbreak: a prospective and periodic assessment study in Taiwan. J Psychiatr Res. 2007 Jan-Feb;41(1-2):119-30. doi: 10.1016/j.jpsychires.2005.12.006. Epub 2006 Feb 7. PMID: 16460760; PMCID: PMC7094424. | C |
| Sutin AR, Luchetti M, Terracciano A. Has loneliness increased during COVID-19? Comment on "Loneliness: A signature mental health concern in the era of COVID-19". Psychiatry Res. 2020 Sep;291:113295. doi: 10.1016/j.psychres.2020.113295. Epub 2020 Jul 13. PMID: 32763553; PMCID: PMC7357499. | S |
| Swaziek Z, Wozniak A. Disparities Old and New in US Mental Health during the COVID-19 Pandemic. Fisc Stud. 2020 Sep;41(3):709-732. doi: 10.1111/1475-5890.12244. Epub 2020 Nov 30. PMID: 33362315; PMCID: PMC7753757. | C |
| Tamayo-Fonseca N, Nolasco A, Moncho J, Barona C, Irles MÁ, Más R, Girón M, Gómez-Beneyto M, Pereyra-Zamora P. Contribution of the Economic Crisis to the Risk Increase of Poor Mental Health in a Region of Spain. Int J Environ Res Public Health. 2018 Nov 9;15(11):2517. doi: 10.3390/ijerph15112517. PMID: 30424004; PMCID: PMC6265689. | E/I |
| Tan BYQ, Chew NWS, Lee GKH, Jing M, Goh Y, Yeo LLL, Zhang K, Chin HK, Ahmad A, Khan FA, Shanmugam GN, Chan BPL, Sunny S, Chandra B, Ong JJY, Paliwal PR, Wong LYH, Sagayanathan R, Chen JT, Ng AYY, Teoh HL, Ho CS, Ho RC, Sharma VK. Psychological Impact of the COVID-19 Pandemic on Health Care Workers in Singapore. Ann Intern Med. 2020 Aug 18;173(4):317-320. doi: 10.7326/M20-1083. Epub 2020 Apr 6. PMID: 32251513; PMCID: PMC7143149. | C |
| Tang F, Liang J, Zhang H, Kelifa MM, He Q, Wang P. COVID-19 related depression and anxiety among quarantined respondents. Psychol Health. 2021 Feb;36(2):164-178. doi: 10.1080/08870446.2020.1782410. Epub 2020 Jun 22. PMID: 32567952. | C |
| Tang, J. Y. ;Han, X. Q. ;Wang, C. Y. ;Wang, X. X. ;Jiang, B. The mental health status and personality character of the cadets who were military officers in the epidemic period of SARS. International Journal of Psychology. 2004. | S |
| Tanne JH. Covid-19: Mental health and economic problems are worse in US than in other rich nations. BMJ. 2020 Aug 6;370:m3110. doi: 10.1136/bmj.m3110. PMID: 32764109. | P |
| Tchimtchoua Tamo AR. An analysis of mother stress before and during COVID-19 pandemic: The case of China. Health Care Women Int. 2020 Nov-Dec;41(11-12):1349-1362. doi: 10.1080/07399332.2020.1841194. Epub 2020 Dec 14. PMID: 33315545. | C |
| Tehrani H. Mental health stigma related to novel coronavirus disease (COVID-19) in older adults. Geriatr Gerontol Int. 2020 Aug;20(8):796-797. doi: 10.1111/ggi.13985. PMID: 32639082; PMCID: PMC7361788. | C |
| Thakur K, Kumar N, Sharma N. Effect of the Pandemic and Lockdown on Mental Health of Children. Indian J Pediatr. 2020 Jul;87(7):552. doi: 10.1007/s12098-020-03308-w. Epub 2020 May 12. PMID: 32394157; PMCID: PMC7214231. | S |
| Thapa SB, Mainali A, Schwank SE, Acharya G. Maternal mental health in the time of the COVID-19 pandemic. Acta Obstet Gynecol Scand. 2020 Jul;99(7):817-818. doi: 10.1111/aogs.13894. PMID: 32374420; PMCID: PMC7267371. | C |
| The Lancet Public Health. COVID-19: from a PHEIC to a public mental health crisis? Lancet Public Health. 2020 Aug;5(8):e414. doi: 10.1016/S2468-2667(20)30165-1. Epub 2020 Jul 23. PMID: 32711683; PMCID: PMC7377773. | P |
| The Lingering Effects of COVID-19. Am J Nurs. 2020 Dec;120(12):15. doi: 10.1097/01.NAJ.0000724176.20278.f5. PMID: 33214356. | C |
| Thern E, de Munter J, Hemmingsson T, Rasmussen F. Long-term effects of youth unemployment on mental health: does an economic crisis make a difference? J Epidemiol Community Health. 2017 Apr;71(4):344-349. doi: 10.1136/jech-2016-208012. Epub 2017 Jan 13. PMID: 28087812; PMCID: PMC5484029. | E/I |
| Thomas RK, Suleman R, Mackay M, Hayer L, Singh M, Correll CU, Dursun S. Adapting to the impact of COVID-19 on mental health: an international perspective. J Psychiatry Neurosci. 2020 Jul 1;45(4):229-233. doi: 10.1503/jpn.200076. PMID: 32584526; PMCID: PMC7828922. | P |
| Thombs BD, Bonardi O, Rice DB, Boruff JT, Azar M, He C, Markham S, Sun Y, Wu Y, Krishnan A, Thombs-Vite I, Benedetti A. Curating evidence on mental health during COVID-19: A living systematic review. J Psychosom Res. 2020 Jun;133:110113. doi: 10.1016/j.jpsychores.2020.110113. Epub 2020 Apr 27. PMID: 32354463; PMCID: PMC7185913. | P |
| Toprak Celenay S, Karaaslan Y, Mete O, Ozer Kaya D. Coronaphobia, musculoskeletal pain, and sleep quality in stay-at home and continued-working persons during the 3-month Covid-19 pandemic lockdown in Turkey. Chronobiol Int. 2020 Dec;37(12):1778-1785. doi: 10.1080/07420528.2020.1815759. Epub 2020 Sep 3. PMID: 32878506. | C |
| Torales J, O'Higgins M, Castaldelli-Maia JM, Ventriglio A. The outbreak of COVID-19 coronavirus and its impact on global mental health. Int J Soc Psychiatry. 2020 Jun;66(4):317-320. doi: 10.1177/0020764020915212. Epub 2020 Mar 31. PMID: 32233719. | C |
| Torjesen I. Covid-19: Mental health services must be boosted to deal with "tsunami" of cases after lockdown. BMJ. 2020 May 15;369:m1994. doi: 10.1136/bmj.m1994. PMID: 32417756. | P |
| Townsend E. COVID-19 policies in the UK and consequences for mental health. Lancet Psychiatry. 2020 Dec;7(12):1014-1015. doi: 10.1016/S2215-0366(20)30457-0. Epub 2020 Oct 15. PMID: 33069273; PMCID: PMC7561293. | P |
| Tromans S, Chester V, Harrison H, Pankhania P, Booth H, Chakraborty N. Patterns of use of secondary mental health services before and during COVID-19 lockdown: observational study. BJPsych Open. 2020 Oct 12;6(6):e117. doi: 10.1192/bjo.2020.104. PMID: 33040771; PMCID: PMC7550872. | C |
| Tull MT, Barbano AC, Scamaldo KM, Richmond JR, Edmonds KA, Rose JP, Gratz KL. The prospective influence of COVID-19 affective risk assessments and intolerance of uncertainty on later dimensions of health anxiety. J Anxiety Disord. 2020 Oct;75:102290. doi: 10.1016/j.janxdis.2020.102290. Epub 2020 Aug 12. PMID: 32823216; PMCID: PMC7422821. | C |
| Usher K, Bhullar N, Jackson D. Life in the pandemic: Social isolation and mental health. J Clin Nurs. 2020 Aug;29(15-16):2756-2757. doi: 10.1111/jocn.15290. Epub 2020 May 6. PMID: 32250493. | S |
| Usher K, Durkin J, Bhullar N. The COVID-19 pandemic and mental health impacts. Int J Ment Health Nurs. 2020 Jun;29(3):315-318. doi: 10.1111/inm.12726. Epub 2020 Apr 10. PMID: 32277578; PMCID: PMC7262128. | C |
| Usher K, Wynaden D, Bhullar N, Durkin J, Jackson D. The mental health impact of COVID-19 on pre-registration nursing students in Australia. Int J Ment Health Nurs. 2020 Dec;29(6):1015-1017. doi: 10.1111/inm.12791. Epub 2020 Sep 24. PMID: 32885572. | C |
| Uvais NA, Aziz F, Hafeeq B. COVID-19-related stigma and perceived stress among dialysis staff. J Nephrol. 2020 Dec;33(6):1121-1122. doi: 10.1007/s40620-020-00833-x. PMID: 32804354; PMCID: PMC7429935. | C |
| Vahia IV, Jeste DV, Reynolds CF 3rd. Older Adults and the Mental Health Effects of COVID-19. JAMA. 2020 Dec 8;324(22):2253-2254. doi: 10.1001/jama.2020.21753. PMID: 33216114. | S |
| Vallée M, Kutchukian S, Pradère B, Verdier E, Durbant È, Ramlugun D, Weizman I, Kassir R, Cayeux A, Pécheux O, Baumgarten C, Hauguel A, Paasche A, Mouhib T, Meyblum J, Dagneaux L, Matillon X, Levy-Bohbot A, Gautier S, Saiydoun G. Prospective and observational study of COVID-19's impact on mental health and training of young surgeons in France. Br J Surg. 2020 Oct;107(11):e486-e488. doi: 10.1002/bjs.11947. Epub 2020 Aug 13. PMID: 32790175; PMCID: PMC7436595. | C |
| van Agteren J, Bartholomaeus J, Fassnacht DB, Iasiello M, Ali K, Lo L, Kyrios M. Using Internet-Based Psychological Measurement to Capture the Deteriorating Community Mental Health Profile During COVID-19: Observational Study. JMIR Ment Health. 2020 Jun 11;7(6):e20696. doi: 10.2196/20696. PMID: 32490845; PMCID: PMC7294997. | P |
| van Giesen, R. I. ;Pieters, R. Climbing out of an economic crisis: A cycle of consumer sentiment and personal stress. Journal of Economic Psychology. 2019. doi 10.1016/j.joep.2018.12.004 | C |
| van Gorp M, Maurice-Stam H, Teunissen LC, van de Peppel-van der Meer W, Huussen M, Schouten-van Meeteren AYN, Grootenhuis MA. No increase in psychosocial stress of Dutch children with cancer and their caregivers during the first months of the COVID-19 pandemic. Pediatr Blood Cancer. 2021 Feb;68(2):e28827. doi: 10.1002/pbc.28827. Epub 2020 Nov 29. PMID: 33251717; PMCID: PMC7744828. | O |
| Varghese A, George G, Kondaguli SV, Naser AY, Khakha DC, Chatterji R. Decline in the mental health of nurses across the globe during COVID-19: A systematic review and meta-analysis. J Glob Health. 2021 Apr 10;11:05009. doi: 10.7189/jogh.11.05009. PMID: 33884193; PMCID: PMC8053406. | C |
| Vaughan, A. The mental health toll of the covid-19 pandemic becomes clear. New Scientist. 2020 | S |
| Venkatesh A, Edirappuli S. Social distancing in covid-19: what are the mental health implications? BMJ. 2020 Apr 6;369:m1379. doi: 10.1136/bmj.m1379. PMID: 32253182. | S |
| Verma K. The mental health impact of the COVID-19 epidemic on college students in India. Asian J Psychiatr. 2020 Oct;53:102398. doi: 10.1016/j.ajp.2020.102398. Epub 2020 Aug 31. PMID: 32956994; PMCID: PMC7458048. | C |
| Vervoort D, Luc JGY, Percy E, Hirji S, Lee R. Assessing the Collateral Damage of the Novel Coronavirus: A Call to Action for the Post-COVID-19 Era. Ann Thorac Surg. 2020 Sep;110(3):757-760. doi: 10.1016/j.athoracsur.2020.04.015. Epub 2020 Apr 30. PMID: 32360875; PMCID: PMC7192104. | P |
| Vigo D, Patten S, Pajer K, Krausz M, Taylor S, Rush B, Raviola G, Saxena S, Thornicroft G, Yatham LN. Mental Health of Communities during the COVID-19 Pandemic. Can J Psychiatry. 2020 Oct;65(10):681-687. doi: 10.1177/0706743720926676. Epub 2020 May 11. PMID: 32391720; PMCID: PMC7502878. | S |
| Vindegaard N, Benros ME. COVID-19 pandemic and mental health consequences: Systematic review of the current evidence. Brain Behav Immun. 2020 Oct;89:531-542. doi: 10.1016/j.bbi.2020.05.048. Epub 2020 May 30. PMID: 32485289; PMCID: PMC7260522. | S |
| Vizheh M, Qorbani M, Arzaghi SM, Muhidin S, Javanmard Z, Esmaeili M. The mental health of healthcare workers in the COVID-19 pandemic: A systematic review. J Diabetes Metab Disord. 2020 Oct 26;19(2):1-12. doi: 10.1007/s40200-020-00643-9. Epub ahead of print. PMID: 33134211; PMCID: PMC7586202. | C |
| Wand APF, Zhong BL, Chiu HFK, Draper B, De Leo D. COVID-19: the implications for suicide in older adults. Int Psychogeriatr. 2020 Oct;32(10):1225-1230. doi: 10.1017/S1041610220000770. Epub 2020 Apr 30. PMID: 32349837; PMCID: PMC7235297. | P |
| Wang C, Chudzicka-Czupała A, Grabowski D, Pan R, Adamus K, Wan X, Hetnał M, Tan Y, Olszewska-Guizzo A, Xu L, McIntyre RS, Quek J, Ho R, Ho C. The Association Between Physical and Mental Health and Face Mask Use During the COVID-19 Pandemic: A Comparison of Two Countries With Different Views and Practices. Front Psychiatry. 2020 Sep 9;11:569981. doi: 10.3389/fpsyt.2020.569981. PMID: 33033485; PMCID: PMC7510452. | E/I |
| Wang C, Pan R, Wan X, Tan Y, Xu L, McIntyre RS, Choo FN, Tran B, Ho R, Sharma VK, Ho C. A longitudinal study on the mental health of general population during the COVID-19 epidemic in China. Brain Behav Immun. 2020 Jul;87:40-48. doi: 10.1016/j.bbi.2020.04.028. Epub 2020 Apr 13. PMID: 32298802; PMCID: PMC7153528. | E/I |
| Wang LQ, Zhang M, Liu GM, Nan SY, Li T, Xu L, Xue Y, Zhang M, Wang L, Qu YD, Liu F. Psychological impact of coronavirus disease (2019) (COVID-19) epidemic on medical staff in different posts in China: A multicenter study. J Psychiatr Res. 2020 Oct;129:198-205. doi: 10.1016/j.jpsychires.2020.07.008. Epub 2020 Jul 14. PMID: 32763586; PMCID: PMC7834267. | C |
| Wang Q, Feng H, Wang M, Xie Y, Hou B, Lu X, Liu Z, Ouyang K, Zhang R, Cai Q, Xu Z, Li H, Chao H, Yang X, Hong Y, Hu X, Liu Z, Liu Y. Mental Health and Psychological Responses During the Coronavirus Disease 2019 Epidemic: A Comparison Between Wuhan and Other Areas in China. Psychosom Med. 2021 May 1;83(4):322-327. doi: 10.1097/PSY.0000000000000892. PMID: 33284256. | C |
| Wang Y, Wu P, Liu X, Li S, Zhu T, Zhao N. Subjective Well-Being of Chinese Sina Weibo Users in Residential Lockdown During the COVID-19 Pandemic: Machine Learning Analysis. J Med Internet Res. 2020 Dec 17;22(12):e24775. doi: 10.2196/24775. PMID: 33290247; PMCID: PMC7747794. | C |
| Wańkowicz P, Szylińska A, Rotter I. Assessment of Mental Health Factors among Health Professionals Depending on Their Contact with COVID-19 Patients. Int J Environ Res Public Health. 2020 Aug 12;17(16):5849. doi: 10.3390/ijerph17165849. PMID: 32806699; PMCID: PMC7459704. | C |
| Webb RT, Kapur N. Suicide, unemployment, and the effect of economic recession. Lancet Psychiatry. 2015 Mar;2(3):196-7. doi: 10.1016/S2215-0366(14)00129-1. Epub 2015 Feb 25. PMID: 26359886. | S |
| Werneck AO, Silva DR, Malta DC, Lima MG, Souza-Júnior PRB, Azevedo LO, Barros MBA, Szwarcwald CL. The mediation role of sleep quality in the association between the incidence of unhealthy movement behaviors during the COVID-19 quarantine and mental health. Sleep Med. 2020 Dec;76:10-15. doi: 10.1016/j.sleep.2020.09.021. Epub 2020 Sep 25. PMID: 33049547; PMCID: PMC7518797. | C |
| Wheaton, M. G. ;Abramowitz, J. S. ;Berman, N. C. ;Fabricant, L. E. ;Olatunji, B. O. Psychological Predictors of Anxiety in Response to the H1N1 (Swine Flu) Pandemic. Cognitive Therapy and Research. 2012. doi 10.1007/s10608-011-9353-3 | C |
| Wilkinson LR. Financial Strain and Mental Health Among Older Adults During the Great Recession. J Gerontol B Psychol Sci Soc Sci. 2016 Jul;71(4):745-54. doi: 10.1093/geronb/gbw001. Epub 2016 Feb 3. PMID: 26843395. | E/I |
| Williamson V, Greenberg N, Bowden G, Rothenfluh D, Nnadi C, Reynolds J. The mental health impact of providing spine care during COVID-19. Spine J. 2020 Sep;20(9):1363-1366. doi: 10.1016/j.spinee.2020.04.019. Epub 2020 Jun 10. PMID: 32534951; PMCID: PMC7286638. | C |
| Willner P, Rose J, Stenfert Kroese B, Murphy GH, Langdon PE, Clifford C, Hutchings H, Watkins A, Hiles S, Cooper V. Effect of the COVID-19 pandemic on the mental health of carers of people with intellectual disabilities. J Appl Res Intellect Disabil. 2020 Nov;33(6):1523-1533. doi: 10.1111/jar.12811. Epub 2020 Sep 21. PMID: 32885897. | C |
| Wind TR, Rijkeboer M, Andersson G, Riper H. The COVID-19 pandemic: The 'black swan' for mental health care and a turning point for e-health. Internet Interv. 2020 Apr;20:100317. doi: 10.1016/j.invent.2020.100317. Epub 2020 Mar 19. PMID: 32289019; PMCID: PMC7104190. | C |
| Winkler P, Formanek T, Mlada K, Kagstrom A, Mohrova Z, Mohr P, Csemy L. Increase in prevalence of current mental disorders in the context of COVID-19: analysis of repeated nationwide cross-sectional surveys. Epidemiol Psychiatr Sci. 2020 Sep 29;29:e173. doi: 10.1017/S2045796020000888. PMID: 32988427; PMCID: PMC7573458. | S |
| Witteveen D, Velthorst E. Economic hardship and mental health complaints during COVID-19. Proc Natl Acad Sci U S A. 2020 Nov 3;117(44):27277-27284. doi: 10.1073/pnas.2009609117. Epub 2020 Oct 12. PMID: 33046648; PMCID: PMC7959574. | C |
| Wiwanitkit V. Health care workers and swine flu. Disaster Med Public Health Prep. 2011 Mar;5(1):9; author reply 9. doi: 10.1001/dmp.2011.4. PMID: 21402821. | C |
| Wu L, Guo X, Shang Z, Sun Z, Jia Y, Sun L, Liu W. China experience from COVID-19: Mental health in mandatory quarantine zones urgently requires intervention. Psychol Trauma. 2020 Aug;12(S1):S3-S5. doi: 10.1037/tra0000609. Epub 2020 Jun 15. PMID: 32538663. | C |
| Xiang X, Ning Y, Kayser J. The Implications of COVID-19 for the Mental Health Care of Older Adults: Insights from Emergency Department Social Workers. J Gerontol Soc Work. 2020 Aug-Oct;63(6-7):662-664. doi: 10.1080/01634372.2020.1779160. Epub 2020 Jun 16. PMID: 32543294. | C |
| Xu J, Ou J, Luo S, Wang Z, Chang E, Novak C, Shen J, Zheng S, Wang Y. Perceived Social Support Protects Lonely People Against COVID-19 Anxiety: A Three-Wave Longitudinal Study in China. Front Psychol. 2020 Nov 6;11:566965. doi: 10.3389/fpsyg.2020.566965. PMID: 33240152; PMCID: PMC7677578. | C |
| Xu, F. M. Yu, P. Feng, H. A research on the relationships of college students' mental health with their coping strategies associated with SARS' stress, personality trait. International Journal of Psychology. 2014. WOS:000226118004122 | C |
| Yang H, Ma J. How an Epidemic Outbreak Impacts Happiness: Factors that Worsen (vs. Protect) Emotional Well-being during the Coronavirus Pandemic. Psychiatry Res. 2020 Jul;289:113045. doi: 10.1016/j.psychres.2020.113045. Epub 2020 Apr 30. PMID: 32388418; PMCID: PMC7190485. | S |
| Yao H, Chen JH, Xu YF. Patients with mental health disorders in the COVID-19 epidemic. Lancet Psychiatry. 2020 Apr;7(4):e21. doi: 10.1016/S2215-0366(20)30090-0. PMID: 32199510; PMCID: PMC7269717. | P |
| Yehudai M, Bender S, Gritsenko V, Konstantinov V, Reznik A, Isralowitz R. COVID-19 Fear, Mental Health, and Substance Misuse Conditions Among University Social Work Students in Israel and Russia. Int J Ment Health Addict. 2020 Jul 6:1-8. doi: 10.1007/s11469-020-00360-7. Epub ahead of print. PMID: 32837438; PMCID: PMC7338139. | C |
| Yip PS, Caine ED. Employment status and suicide: the complex relationships between changing unemployment rates and death rates. J Epidemiol Community Health. 2011 Aug;65(8):733-6. doi: 10.1136/jech.2010.110726. Epub 2010 Nov 28. PMID: 21115474. | C |
| Yohannes AM. COPD patients in a COVID-19 society: depression and anxiety. Expert Rev Respir Med. 2021 Jan;15(1):5-7. doi: 10.1080/17476348.2020.1787835. Epub 2020 Jul 5. PMID: 32578464. | P |
| Yuen KF, Wang X, Ma F, Li KX. The Psychological Causes of Panic Buying Following a Health Crisis. Int J Environ Res Public Health. 2020 May 18;17(10):3513. doi: 10.3390/ijerph17103513. PMID: 32443427; PMCID: PMC7277661. | C |
| Zandifar A, Badrfam R, Mohammadian Khonsari N, Assareh M, Karim H, Azimzadeh M, Noori Sepehr M, Tajbakhsh R, Rahimi F, Ghanipour N, Agoushi A, Hassani Gelsefid S, Etemadi F, Qorbani M. COVID-19 and medical staff's mental health in educational hospitals in Alborz Province, Iran. Psychiatry Clin Neurosci. 2020 Sep;74(9):499-501. doi: 10.1111/pcn.13098. Epub 2020 Jul 17. PMID: 32592626; PMCID: PMC7362126. | C |
| Zapata Moya ÁR, Navarro Yáñez CJ. Urban regeneration policies and mental health in a context of economic crisis in Andalusia (Spain). J Hous Built Environ. 2020 Aug 19:1-13. doi: 10.1007/s10901-020-09774-0. Epub ahead of print. PMID: 32839662; PMCID: PMC7437960. | S |
| Zavras D, Tsiantou V, Pavi E, Mylona K, Kyriopoulos J. Impact of economic crisis and other demographic and socio-economic factors on self-rated health in Greece. Eur J Public Health. 2013 Apr;23(2):206-10. doi: 10.1093/eurpub/cks143. Epub 2012 Oct 23. PMID: 23093716. | O |
| Zhang B, Zaman A, Silenzio V, Kautz H, Hoque E. The Relationships of Deteriorating Depression and Anxiety With Longitudinal Behavioral Changes in Google and YouTube Use During COVID-19: Observational Study. JMIR Ment Health. 2020 Nov 23;7(11):e24012. doi: 10.2196/24012. PMID: 33180743; PMCID: PMC7685700. | C |
| Zhang WR, Wang K, Yin L, Zhao WF, Xue Q, Peng M, Min BQ, Tian Q, Leng HX, Du JL, Chang H, Yang Y, Li W, Shangguan FF, Yan TY, Dong HQ, Han Y, Wang YP, Cosci F, Wang HX. Mental Health and Psychosocial Problems of Medical Health Workers during the COVID-19 Epidemic in China. Psychother Psychosom. 2020;89(4):242-250. doi: 10.1159/000507639. Epub 2020 Apr 9. PMID: 32272480; PMCID: PMC7206349. | C |
| Zhang Y, Cao X, Wang P, Wang G, Lei G, Shou Z, Xie S, Huang F, Luo N, Luo M, Bian Y, Zhang J, Xiao Q. Emotional "inflection point" in public health emergencies with the 2019 new coronavirus pneumonia (NCP) in China. J Affect Disord. 2020 Nov 1;276:797-803. doi: 10.1016/j.jad.2020.07.097. Epub 2020 Jul 19. PMID: 32738664; PMCID: PMC7369017. | C |
| Zhong BL, Zhou DY, He MF, Li Y, Li WT, Ng CH, Xiang YT, Chiu HF. Mental health problems, needs, and service use among people living within and outside Wuhan during the COVID-19 epidemic in China. Ann Transl Med. 2020 Nov;8(21):1392. doi: 10.21037/atm-20-4145. PMID: 33313137; PMCID: PMC7723535. | C |
| Zhou Y, MacGeorge EL, Myrick JG. Mental Health and Its Predictors during the Early Months of the COVID-19 Pandemic Experience in the United States. Int J Environ Res Public Health. 2020 Aug 31;17(17):6315. doi: 10.3390/ijerph17176315. PMID: 32877985; PMCID: PMC7503583. | C |
| Zhu S, Wu Y, Zhu CY, Hong WC, Yu ZX, Chen ZK, Chen ZL, Jiang DG, Wang YG. The immediate mental health impacts of the COVID-19 pandemic among people with or without quarantine managements. Brain Behav Immun. 2020 Jul;87:56-58. doi: 10.1016/j.bbi.2020.04.045. Epub 2020 Apr 18. PMID: 32315758; PMCID: PMC7165285. | C |
| Zhuo K, Gao C, Wang X, Zhang C, Wang Z. Stress and sleep: a survey based on wearable sleep trackers among medical and nursing staff in Wuhan during the COVID-19 pandemic. Gen Psychiatr. 2020 Jun 16;33(3):e100260. doi: 10.1136/gpsych-2020-100260. Erratum in: Gen Psychiatr. 2020 Jul 21;33(4):e100260corr1. PMID: 32596641; PMCID: PMC7299005. | C |
| Zitting KM, Lammers-van der Holst HM, Yuan RK, Wang W, Quan SF, Duffy JF. Google Trends reveals increases in internet searches for insomnia during the 2019 coronavirus disease (COVID-19) global pandemic. J Clin Sleep Med. 2021 Feb 1;17(2):177-184. doi: 10.5664/jcsm.8810. PMID: 32975191; PMCID: PMC7853234. | E/I |
| Zivin K, Paczkowski M, Galea S. Economic downturns and population mental health: research findings, gaps, challenges and priorities. Psychol Med. 2011 Jul;41(7):1343-8. doi: 10.1017/S003329171000173X. Epub 2010 Sep 14. PMID: 20836907; PMCID: PMC3846090. | P |
| Zolotov Y, Reznik A, Bender S, Isralowitz R. COVID-19 Fear, Mental Health, and Substance Use Among Israeli University Students. Int J Ment Health Addict. 2020 Jun 15:1-7. doi: 10.1007/s11469-020-00351-8. Epub ahead of print. PMID: 32837432; PMCID: PMC7299139. | C |
