# Supplementary material for: Effects of the COVID-19 pandemic and previous pandemics, epidemics and economic crises on mental health: systematic review
Source: BJPsych Open. 2022 Oct 10;8(6):e181. doi: 10.1192/bjo.2022.587 (PMC9551492; doi:10.1192/bjo.2022.587)
Supplement: Supplementary file 1 [file S2056472422005877sup001.zip › S2056472422005877sup004.docx]

**Table 1: Characteristics of studies with COVID-19 exposure**

| **First Author** | **Quality** | **Year** | **Study design** | **Country** | **Main mental health outcome measure** | **Follow up period** |
| --- | --- | --- | --- | --- | --- | --- |
| Meda N(14) | ** | 2020 | Repeated cross-Sectional | Italy | BDI-2*, BAI* | 8 months 2 years October 2019-June 2020 |
| Krendl A C(15) | ** | 2020 | Longitudinal cohort | USA | PHQ-8* | 1 year summer/fall 2019 and April/May 2020 |
| Pan K-Y(16) | ** | 2020 | Longitudinal cohort | Netherlands | QUIDS*, BAI* | 4-14 years 2006-2016 and April- May 2020 |
| Ettman C K(17) | ** | 2020 | Longitudinal cohort | USA | Depression symptoms | 2-3 years 2017-2018 and March- April 2020 |
| Daly M(18) | ** | 2020 | Longitudinal cohort | UK | GHQ-12* | 3 years 2017-2020 |
| Puhl R M(19) | ** | 2020 | Longitudinal cohort | USA | Depression scale, 6 items | 2- 10 years 2010-2018 and 2020 |
| Villani E R(20) | ** | 2020 | Longitudinal cohort | Italy | DRS* | 1-5 years 2015-2019 |
| Gallagher S(21) | *** | 2020 | Longitudinal cohort | UK | GHQ-12* | 2017–2019 and May 2020 |
| Wanberg C R(22) | ** | 2020 | Longitudinal cohort | USA | PHQ*-8 | 1 year April-June 2019 and April 2020 |
| Hamadani J D(23) | ** | 2020 | Longitudinal cohort | Bangladesh | CES-D* | 3 months- 3 years 2017- Feb 2020, May -June 2020. Median days 708 (baseline) and 347 (RCT endline) days before covid lockdown period |
| Lee C M(24) | ** | 2020 | Longitudinal cohort | USA | Three-item Loneliness Scale, PHQ-4* | 3-4 months Jan 2020 and April/May 2020 |
| Zanardo V(25) | ** | 2020 | Repeated cross sectional | Italy | Postpartum depression EPDS* total score | 1 year 2019-2020 |
| Wong S Y S(26) | ** | 2020 | Longitudinal cohort | Hong Kong | PHQ-9*, GAD-7* | 4 years 2016-2020 |
| Saraswathi I(27) | *** | 2020 | Longitudinal cohort | India | DASS21* | 6 months Dec 2019 to June 2020 |
| Stojanov A(28) | ** | 2020 | Longitudinal cohort | Serbia | HARS*, HRSD* | 1 year 2019-2020 |
| Kwong A S F(29) | *** | 2021 | Longitudinal cohort | UK | PHQ-9*, GAD-7*, Short Mood and Feelings Questionnaire, Short Warwick Edinburgh Mental Wellbeing Scale | 9- 29 years ALSPAC:1991- 1992 to 9 April and 14 May 2020.Generation Scotland: 2006 -2011 to 7 April and 17 May 2020. |
| Thombs B D(30) | ** | 2020 | Longitudinal cohort | Canada, France, UK, USA | PROMIS anxiety scale, PHQ-8* | 4-9 months July -Dec 2019 and April 9 to April 27 2020 |
| Jacob L(31) | *** | 2020 | Repeated cross-sectional | Germany | ICD-10 anxiety disorders | 1 year Jan-June 2019 and Jan-June 2020 |
| Titov N(32) | ** | 2020 | Repeated cross sectional | Australia | GAD-7*, PHQ-9* | 1 year 2019 and 2020 |
| Zhao S Z(33) | ** | 2020 | Repeated cross-sectional | China | GAD-2*, PHQ-2* | 3-4 years 2016, 2017 and April 2020 |
| Huckins J F(34) | ** | 2020 | Longitudinal cohort | USA | PHQ-4* | 2-3 years 2017/2018 and 2020 |
| Lim S L(35) | ** | 2020 | Longitudinal cohort | Singapore | EQ-5D | 2 years 2018-2020 |
| Li H Y(36) | ** | 2020 | Longitudinal cohort | China | Positive Affect | 3 months 20 Dec 2019 and Feb 2020 |
| Chen I H(37) | ** | 2020 | Longitudinal cohort | China | DASS-21* | 1 year 2019-2020 |
| Magson N R(38) | ** | 2021 | Longitudinal cohort | Australia | SCAS-C*, SMFQ-C * | 1 years 2019-2020 |
| Creese B(39) | ** | 2020 | Longitudinal cohort | UK | PHQ-9*, GAD-7* | 5 years 2015-2020 |
|  |  |  |  |  |  |  |
| Twenge J M(40) | ** | 2020 | Repeated cross sectional | USA | PHQ ‐2*, GAD-2* | approx. 9 months Jan-Jun 2019 and April-May 2020 |
| Zhang Y(41) | ** | 2020 | Longitudinal cohort | South Sudan | PSS-10*, GAD-7*, PHQ-9* | 3-9 months Nov 2019, Feb, May and August 2020 |
| Zhang B(42) | ** | 2020 | Longitudinal cohort | USA | GAD-7*, PHQ-9* | 1 month Jan - Feb and March – May 2020 |
| Peters A(43) | ** | 2020 | Longitudinal cohort | Germany | PHQ-9*, GAD-7 | 3-6 years 2014- 2019 and 30 April- 29 May 2020 |
| Elmer T(44) | ** | 2020 | Longitudinal cohort | Switzerland | CES-D*, GAD-7* | 2 years 2018-2020 |
| Janssen L H C(45) | ** | 2020 | Longitudinal cohort | Netherlands | PHQ-9* | 1-2 years 2018–2019 and 14–28 April 2020 |
| Gallagher S(46) | ** | 2020 | Longitudinal cohort | UK | GHQ-12* | 1-3 years 2017–19 and 2020 |
| McArthur C(47) | ** | 2021 | Longitudinal cohort | Canada | Depression DRS* | 3.5 years Jan 2017-June 2020 and March 2020 |
| Pinkham A E(48) | ** | 2020 | Longitudinal cohort | USA | Self-rated mood and wellness | 1-2 years 2018, 2019 and 2020 |
| Sturman E D(49) | * | 2020 | Repeated cross-sectional | USA | CESD* |  |
| van der Velden P(50) | ** | 2020 | Longitudinal cohort | Netherlands | MHI-5* | 2 years 2018-2020 |
| Baiano C(51) | ** | 2020 | Longitudinal cohort | Italy | Anxiety Sensitivity Index-3 | 2 -5 months 4 Nov 2019–17 Feb 2020 and 26-30 April 2020 |
| Pariente G(52) | *** | 2020 | Repeated cross-sectional | Israel | EPDS* | 3-4 years Nov 2016- April 2017 and March -April 2020. |
| Ubara A(53) | ** | 2020 | Longitudinal cohort | Japan | PHQ-9* | 1 year 2019 and 2020 |
| Bolatov A K(54) | ** | 2021 | Repeated cross-sectional | the Republic of Kazakhstan | CBI-S*, PHQ-9*, GAD-7* | 4 months Oct- Nov 2019 and April 2020 |
| Li W W(55) | ** | 2020 | Longitudinal cohort | China | DASS-21* | 6-7 months Nov 2019 to May - June, 2020 |
| Williams R(56) | *** | 2020 | Repeated cross-sectional | UK | Common mental health problem diagnoses and medication prescriptions | Predicted expected and observed numbers of first diagnoses and first prescriptions between March 1 and May 31, 2020, |
| Mitchell T O(57) | *** | 2020 | Repeated cross-sectional | USA | Suicide rates | 2 months March 10th to May 20th, 2020, compared with 5 year average |
| Leske S(58) | *** | 2020 | Repeated cross-sectional | Australia | Probable and beyond reasonable doubt suicides | 2015-2020 |
| Isumi A(59) | *** | 2020 | Repeated cross-sectional | Japan | Suicide rates | 1-2 years 2018 - 2019 and 2020 |
| Calderon-Anyosa R(60) | *** | 2020 | Repeated cross-sectional | Peru | Suicide | 3 years Jan 2017 -Sep 2020 |
| Sutin A R(61) | ** | 2020 | Longitudinal cohort | USA | PHQ-2* | 1-3 months Jan 31- Feb 10, March 18 -29, and April 23- 29 2020 |
| Pierce M(62) | *** | 2020 | Longitudinal cohort | UK | GHQ-12* | 1 year 2019 - April 2020 |
| Banks J(63) | ** | 2020 | Longitudinal cohort | UK | GHQ*-12 | 11 months Jan 2017-Nov 2019 and April 2020 |
| Gray N(64) | ** | 2020 | Repeated cross-sectional | UK | WEMWBS* and K10* | 1 year, & 2 months April 2018-March 2019 and 9 June -13 July, 2020 |
| Daly M(65) | *** | 2021 | Repeated cross-sectional | USA | PHQ-2 * | 2-3 years 2017-2018 and 2020 |
| Sibley C G(66) | ** | 2020 | Longitudinal cohort | New Zealand | K6* | 6-3 months Oct-Dec 2019 and 2020 |
| Twenge J M(67) | *** | 2020 | Repeated cross-sectional | USA | K-6* | 2 years 2018- and April 2020 |
| McGinty E E(68) | ** | 2020 | Longitudinal cohort | USA | Psychological distress | 2 years 2018 - April 2020 |
| Niedzwiedz C L(69) | *** | 2020 | Longitudinal cohort | UK | GHQ-12* | 1 year 2019 and April 2020 |
| Sønderskov K M(70) | * | 2020 | Repeated cross- sectional | Denmark | Wellbeing | 4 years 2016-2020 |
| Bierman A(71) | ** | 2020 | Repeated cross-sectional | Canada | Distress | 6 months Sep 2019 -March 2020 |
| Ran M S(72) | ** | 2020 | Repeated cross-sectional | China | GHQ-12*, SAS*, SDS* | 8 months Jan -May 2019 and Feb 2020 |
| Shen J(73) | ** | 2020 | Longitudinal cohort | UK | GHQ-12* | 1-3 years 2017-2019 follow-up 2020 |
| Reverté-Villarroya S(74) | ** | 2021 | Repeated cross-sectional | Spain | GHQ* | 3 years 2017 and 2020 |
| Macdonald B(75) | ** | 2020 | Longitudinal cohort | Switzerland | Positive and negative affect and loneliness | 1 year 2019 (for 21 days) & 2020 (4-weeks) |
| Savage M J(76) | ** | 2020 | Longitudinal cohort | UK | WEMWBS* and PSS* | 6 months Oct 2019 - April 2020 |
| Ohliger E(77) | *** | 2020 | Repeated cross-sectional | USA | ICD-10 classification of mental disorders | 9 months- 1 year Feb-April 2019 and Feb-April 2020 |
| Copeland W E(78) | ** | 2021 | Longitudinal cohort | USA | 2 mood scale items | 2-4 months Early 2020 and May-Jun 2020 |
| Dragun R(79) | ** | 2021 | Repeated cross-sectional | Croatia | PSS-10* | 1-2 years 2018 and 2019 vs 2020 |
| Castellini G(80) | ** | 2020 | Longitudinal cohort | Italy | BSI* and EDE-Q* | 5 months Nov 2019 - Jan 2020 and April- May 2020 |
| Giordano A(81) | ** | 2021 | Longitudinal cohort | Italy | Epileptic seizure frequency | 1-3  Months Jan- Feb and March-April 2020 |
| Gomez S(82) | ** | 2020 | Longitudinal cohort | USA | Stanford Professional Fulfillment Index | 1-4   1-2 years Dec 2018- Jan 2019 and July 2019 - May 2020 |
| Breslau J(83) | *** | 2020 | Longitudinal cohort | USA | Psychological distress K-6* | 1-year 2019 and 2020 |
| Penner F(84) | ** | 2021 | Longitudinal cohort | USA | BPM* |  |
| Rutherford B R(85) | ** | 2020 | Longitudinal cohort | USA | PCL-5*, HARS*, HRSD* |  |
| Benham G(86) | ** | 2020 | Repeated cross-sectional | USA | PSS-10* | 1 year Spring 2019 -Summer 2020 |
| Kivi M(87) | ** | 2021 | Longitudinal cohort | Sweden | Levels of Worry, Risk Perception, and Social Distancing in Relation to COVID-19 | 5 years Yearly from 2015 to 2020 |
| Schäfer S K(88) | ** | 2020 | Longitudinal cohort | Germany | SOC (9-item Antonovsky scale), Mini-Symptom Checklist | 1 month Feb 2020 and March 2020 |
| van Tilburg T G(89) | *** | 2020 | Longitudinal cohort | Netherlands | Social and emotional loneliness (three items), mental health inventory (five items) | 1 year 2019-2020 |
| van Gorp M(90) | ** | 2021 | Longitudinal cohort | Netherlands | PedsQL generic and PedsQL fatigue of children (caregiver about child) | 5 months 1 Jan - 1 June 2020 |
| Dragovic M(91) | *** | 2020 | Repeated cross sectional | Australia | ICD-10 principal psychiatric diagnoses | 1 year 2019 and 2020 |
| Stein H C(92) | *** | 2020 | Repeated cross-sectional | Italy | ER visits for mental‐health‐related conditions | 1 year Jan – May, 2019 and Jan – March, 2020 and March – May, 2020 |
| Capuzzi E(93) | *** | 2020 | Repeated cross-sectional | Italy | Emergency psychiatric consultations | 1 year Feb - May 2019 and Feb - May 2020 |
| Pignon B(94) | *** | 2020 | Repeated cross-sectional | France | Psychiatric emergency consultations | 1 year 2019-2020 |
| Leff R A(95) | *** | 2021 | Repeated cross-sectional | USA | Mental health-related diagnoses at Paediatric emergency department | 1 year March 2019 and March 2020 |
| Abbas M J(96) | *** | 2021 | Repeated cross-sectional | UK | Referrals to crisis resolution and home treatment team and acute care mental health admissions | 1 year 2019 and 2020 March-April |
|  |  |  |  |  |  |  |
| Grimshaw B(97) | *** | 2021 | Repeated cross-sectional | UK | Mental health admissions, ICD-10* | 1 year 2019 and 2020 |
| Chen S(98) | *** | 2020 | Repeated cross-sectional | UK | Referrals per day to secondary care mental health services. Routine, urgent/emergency | 1-year 2019 and 2020 |
| Varani S(99) | ** | 2020 | Longitudinal cohort | Italy | GHQ-12* | 4 years 2016 and 2020 |
| Joyce L R(100) | *** | 2021 | Repeated cross-sectional | New Zealand | Emergency department mental health presentations (medical records) | 2019 and 2020. Baseline at pre-lockdown, 33 days prior to lock-down period |

**Table legend:** * CES-D Center for Epidemiological Studies Depression Scale, EPDS Edinburgh Postnatal Depression, DASS21 Depression and Anxiety Scale 21, WEMWBS Warwick-Edinburgh Mental Well- being Scale, PCL-5 Post-traumatic Stress Disorder Checklist, BSI Brief Symptom Inventory, PHQ-2 PHQ-10 Patient Health Questionnaire, K-10, K-6 Kessler Psychological Distress Scale, GHQ-12 General Health Questionnaire -12, PSS Perceived Stress Scale, DRS Depression Rating Scale, MHI-5 Mental Health Inventory, 5 items, BPM Brief Problem Monitor, HARS Hamilton Anxiety Rating Scale, HRSD Hamilton rating Scale for Depression, EDE-Q Eating Disorder Examination Questionnaire, GAD-7, GAD-2 General Anxiety Disorder, CBI-S Copenhagen Burnout Inventory, SAS Self-rating Anxiety Scale, SDS Self-Rating Depression Scale, BAI Beck Anxiety Inventory, QIDS Quick Inventory of Depressive Symptoms, SCAS-C Spence Children’s Anxiety Scale, SMFQ-C Short Mood and Feelings Questionnaire—Child Version, BDI Beck Depression Inventory, EQ-5D Euroqol 5 dimensions. Quality assessment according to Newcastle-Ottawa criteria(9): High quality = *** Fair quality =** Low quality =*

**Table 2: Characteristics of studies with economic crisis exposure**

| **First Author** | **Quality** | **Year** | **Study design** | **Country** | **Main mental health outcome measure** | **Follow up period length** |
| --- | --- | --- | --- | --- | --- | --- |
| Wang J(101) | ** | 2010 | Repeated Cross-sectional | Canada | WHO’s CIDI-Auto 2.1* | 1 year 2008-2009 |
| Lee S(102) | ** | 2010 | Repeated cross-sectional | Hong Kong | Major Depressive Episode according to DSM-IV | 2 years and 3 months Jan-Feb 2007 and April-May 2009) |
| Riumallo-Herl C(103) | *** | 2014 | Longitudinal Cohort | USA | EURO-D*, CES-D* | 6 years 2004-2010 |
| Tapia Granados J A(104) | *** | 2018 | Longitudinal cohort | USA | CES-D* | Up to 24 years 1987–1988, 1990–1991, 1992–1993, 1995–1996, 2000–2001, 2005–2006 and 2010–2011 |
| Cagney K A(105) | ** | 2014 | Longitudinal cohort | USA | CES-D* | 5 – 6 years 2005-2006 and 2010-2011 |
| Chaves C(106) | ** | 2018 | Repeated cross-sectional | Spain | CESD8* | 7 years 2006-2013 |
| Sargent-Cox K(107) | ** | 2011 | Longitudinal cohort | Australia | Goldberg depression scale | 3-5 years 2005-2006 to 2009-2010 |
| Mehta K(108) | ** | 2015 | Repeated cross-sectional | USA | PHQ-9* | 7 years 2005-2012 |
| Pruchno R(109) | ** | 2017 | Longitudinal cohort | USA | CES-D* | 5 years Nov 2006, April 2008 and Jan 2011- May 2012 |
| Wang H(110) | ** | 2018 | Longitudinal cohort | USA | K6* | Up to 10 years 2003-2005, 2007-2009,2011-2013 |
| Dagher R K(111) | ** | 2015 | Repeated cross sectional | USA | SF-12 MCS*, K6 | 4-6 years 2005-2006, 2010-2011 |
| Shi Z(112) | ** | 2010 | Repeated cross-sectional | Australia | K-10*, GHQ*-28 | 7 years 2002-2009 |
| McInerney M(113) | ** | 2013 | Longitudinal cohort | USA | CES-D* | 2 years 2006 and 2008 |
| Forbes M K(114) | *** | 2019 | Longitudinal cohort | USA | CIDI-SF* | 10-11 years 2003/2004-2012/2013 |
| Reibling N(115) | ** | 2017 | Repeated cross-sectional | 21 European nations | CES-D* | 8 years 2006-2014 |
| Merzagora I(116) | *** | 2016 | Repeated cross-sectional | Italy | Suicide | 11 years 2002-2013 |
| De Vogli R(117) | *** | 2014 | Repeated cross-sectional | Italy | Mortality due to mental and behavioural disorders | 10 years 2000-2010 |
| Zilidis C(118) | *** | 2020 | Repeated cross-sectional | Greece | Suicide | 15 years 2001-2016 |
| Madianos M G(119) | *** | 2014 | Repeated cross-sectional | Greece | Suicide | 21 years 1990-2011 |
| Vlachadis N(120) | *** | 2014 | Repeated cross sectional | Greece | Suicide | 2 years 2010-2012 and time series of suicide rates over several years |
| Branas C C(121) | *** | 2014 | Repeated cross-sectional | Greece | Suicide | 29 years 1983-2012 |
| Papaslanis T(122) | *** | 2016 | Repeated cross-sectional | Greece | Suicide | 20 years 1992 to 2012 (only extracting data from 2006 to 2012 |
| Kontaxakis V(123) | *** | 2013 | Repeated cross-sectional | Greece | Suicide | 10 years 2001-2011 |
| Lopez Bernal J A(124) | *** | 2013 | Repeated cross-sectional | Spain | Suicide | 5 years 2005-2010 |
| Córdoba-Doña J A(125) | *** | 2014 | Repeated cross sectional | Spain | Suicide attempts | 1-9 years 2003–2007, 2008–2012 |
| Reeves A(126) | *** | 2014 | Repeated cross-sectional | Europe, Canada, USA | Suicide | 3 years 2007-2010 |
| Stuckler D(127) | *** | 2011 | Repeated cross-sectional | Austria, Finland, Greece, Ireland, the Netherlands, and the UK, Czech Republic, Hungary, Lithuania, and Romania | Suicide | 2 years 2007-2009 |
| Laanani M(128) | *** | 2014 | Longitudinal cohort | Western EU countries (Austria, Finland, France,Germany, the Netherlands, Spain, Sweden and the UK) | Relative risks of increase in suicide rates for a 10% increase in unemployment rate | 10 years 2000–2010 |
| Saurina C(129) | *** | 2013 | Repeated cross-sectional | England | Suicide | 17 years 1993-2010 |
| Barr B(130) | *** | 2012 | Repeated cross-sectional | England | Number of excess suicides | 10 years 2000-2010 |
| Agrrawal P(131) | *** | 2017 | Repeated cross-sectional | USA | Suicide | 8 years 2005-2013 |
| Kerr W C(132) | *** | 2017 | Repeated cross-sectional | USA | Suicide | 6 years 2005-2011 |
| Carriere D E(133) | *** | 2019 | Repeated cross-sectional | USA | Suicide | 14 years 2002-2016 |
| Chan C H(134) | *** | 2013 | Repeated cross-sectional | South Korea | Suicide | 2003-2011 |
| Rachiotis G(135) | *** | 2015 | Repeated cross-sectional | Greece | Suicide | 9 years 2003-2012 |
| Alexopoulos E C(136) | *** | 2019 | Repeated cross-sectional | Greece | Suicide | 13 years 2000–2013 |
| Coope C(137) | *** | 2014 | Repeated cross-sectional | UK | Suicide | 10 years 2001-2011 |
| Ruiz-Perez I(138) | *** | 2017 | Repeated cross-sectional | Spain | Suicide | 10 years 2002-2012 |
| Corcoran P(139) | *** | 2015 | Repeated cross-sectional | Ireland | Suicide | 32 years 1980-2012 |
| Mattei G(140) | *** | 2019 | Repeated cross-sectional | Italy | Suicide | 29-38 years 1977–2015, 1983–2012 |
| Milner A(141) | *** | 2014 | Repeated cross-sectional | Australia | Suicide | 2 years 2007–2009 |
| Iglesias-García C(142) | *** | 2017 | Repeated cross-sectional | Spain | Suicide | 14 years 1999-2013 |
| Rivera B(143) | *** | 2016 | Repeated cross-sectional | Spain | Suicide | 9 years 2004 and 2013 |
| López-Contreras N(144) | *** | 2019 | Repeated cross-sectional | Spain | Suicide | 10 years 2006-2016 |
| Cylus J(145) | *** | 2014 | Repeated cross sectional | USA | Suicide | 40 years 1968-2008 |
| Fountoulakis K N(146) | *** | 2014 | Repeated cross-sectional | Austria, Belgium, Estonia, Finland, France, Germany, Greece, Italy, Ireland, Netherlands, Portugal, Slovakia, Slovenia, Spain, Bulgaria, Croatia, Czech Rep, Denmark, Hungary, Latvia, Lithuania, Poland, Romania, Sweden, UK, Montenegro, Norway, Serbia, Switzerland. | Suicide | 11 years 2000–2011 |
| Reeves A(147) | *** | 2015 | Repeated cross-sectional | 24 EU countries | Suicide | 30 years 1981-2011 |
| Chang S S(148) | *** | 2013 | Repeated cross-sectional | 54 countries | Suicide | 9 years 2000-2009 |
| Mattei G(149) | *** | 2019 | Repeated cross-sectional | Italy | Suicide | 24 years 1990-2014 |
| Mattei G(150) | *** | 2014 | Repeated cross-sectional | Italy | Suicide | 10 years 2000-2010 |
| Paraschakis A(151) | *** | 2018 | Repeated cross-sectional | Greece | Suicide | 9 years 2006-2015 |
| Basta M(152) | *** | 2018 | Repeated cross-sectional | Crete, Greece | Suicide | 14 years 1999 and 2013 |
| Blomqvist S(153) | ** | 2014 | Repeated cross-sectional | Sweden | GHQ12* | 4 years 2006-2010 |
| Thomson R M(154) | *** | 2018 | Repeated cross-sectional | England | GHQ G-12* | 23 years timeline 1991-2014 |
| Thomson R M(155) | ** | 2018 | Repeated cross-sectional | England | GHQ-12* | 23 years 1991-2014 |
| Odone A(156) | *** | 2018 | Repeated cross-sectional | Italy | MSC* derived from SF-12* | 8 years 2005-2013 |
| Urbanos-Garrido R M(157) | ** | 2014 | Repeated cross-sectional | Spain | Self-assessed health | 5-6 years 2006 and 2011-2012 |
| Tamayo-Fonseca N(158) | ** | 2018 | Repeated cross-sectional | Spain | questions corresponding to GHQ-12* | 5 years 2005 and 2010 |
| Katikireddi S V(159) | ** | 2012 | Repeat cross-sectional | England | GHQ-12* | 19 years 1991-2010 |
| Parker P D(160) | ** | 2016 | Repeated cross-sectional | Australia | a measure similar to the Personal Wellbeing Index | 7-10 years 1997-2013 |
| Gonza G(161) | ** | 2016 | Repeated cross-sectional | 36 countries, mainly European | Subjective Well-Being assessed with two questions | 10 years 2002-2012 |
| Gudmundsdottir D G(162) | *** | 2011 | Longitudinal cohort | Iceland | Happiness question: ‘Taking all things together, how happy would you say you are?’’, 1-10 scale | 2 years Oct 2007 and Nov 2009 |
| Sifaki-Pistolla D(163) | ** | 2018 | Repeated Cross-sectional | Greece | DASS-21* | 9 years Dec 2008 and Feb 2017 |
| Golberstein E(164) | ** | 2019 | Repeated cross-sectional | USA | SDQ* | 12 years 2001–2013 |
| Houdmont J(165) | ** | 2012 | Repeated cross-sectional | Ireland | Absence attributable to work-related stress | 4 years 2005-2009 |
| Hauksdóttir A(166) | *** | 2013 | Longitudinal cohort | Iceland | PSS-4* | 2 years 2007-2009 |
| Bartoll X(167) | ** | 2013 | Repeated cross-sectional | Spain | GHQ-12* | 4-6 years 2006-2007 to 2011-2012 |
| Malard L(168) | *** | 2015 | Longitudinal cohort | France | MINI* | 4 years 2006-2010 |
| Rajmil L(169) | ** | 2013 | Repeated cross-sectional | Spain | Parent version of SDQ* | 6 years 2006-2012 |
| Lindström M(170) | *** | 2016 | Longitudinal cohort | UK | GHQ-12* | 1 year 2007-2008 |
| Ruiz-Pérez I(171) | *** | 2017 | Repeated Cross-sectional | Spain | GHQ* |  |
| Kozman D(172) | *** | 2012 | Repeated cross-sectional | USA | Prescription drugs utilisation | 3 years 2007-2010 |
| Bubonya M(173) | ** | 2019 | Longitudinal cohort | Australia | SDQ* | 4 years 2007-2011 |
| Boyce C J(174) | *** | 2018 | Longitudinal cohort | UK | GHQ-12* and a life satisfaction measure | 3-4 years 2006-2007 and 2009-2010 |
| Barrett A(175) | * | 2014 | Repeated cross-sectional | Ireland | CASP-12 quality of life | 5-7 years 2006-2007 and 2012-2013 |
| Sarracino F(176) | ** | 2020 | Repeated cross-sectional | Belgium, Bulgaria, Switzerland, Cyprus, Germany, Denmark, Estonia, Spain, Finland, France, Great Britain, Hungary, Ireland, Netherlands, Norway, Poland, Portugal, Russian Fed., Sweden, Slovenia, Slovakia, Ukraine, | Well-being assessed in European Social Survey | 2 years 2006 to 2008 |
| Rathmann K(177) | ** | 2016 | Repeated cross-sectional | 31 countries in Europe, North America and Israel | HBSC* | 4 years 2005/2006 and 2009/2010 |
| Wang Y(178) | *** | 2020 | Longitudinal cohort | Italy | Inpatient admissions for affective disorders (ICD-9: 296.0-296.9) | 8 years 2007-2015 |
| Bonnie Lee C(179) | *** | 2017 | Repeated cross-sectional | Taiwan | Hospitalization due to depressive illnesses | 5 Years 2007-2012 |
| Gili M(180) | ** | 2012 | Repeated cross-sectional | Spain | PRIME-MD* | 3-4 years 2006/2007 and 2010/2011 |
| Kendrick T(181) | *** | 2015 | Repeated cross-sectional | UK | GP recording of Depression | 10 years 2003-2013 |
| Medel-Herrero A(182) | *** | 2017 | Repeated cross-sectional | Spain | Psychiatric hospital admissions | 5-11 years July 2002-March 2008 and April 2008 to Dec 2013 |
| Hawton K(183) | *** | 2016 | Repeated cross-sectional | UK | Rates of self-harm | 9 years 2001-2010 |
| Chen J(184) | *** | 2014 | Repeated cross- sectional | USA | Physician visits, Prescription drug utilization | 9 years 2000-2009 |

**Table legend:** * GHQ-12 General Health Questionnaire, CES-D Center for Epidemiologic Studies Depression, CIDI-SF The World Health Organization’s Composite International Diagnostic Interview Short Form, MINI The Mini International Neuropsychiatric Interview, HBSC Health Behaviour in School- aged Children, PSS-4 Perceived Stress Scale, SF-12 MCS the 12-item Short Form Mental Health Summary, EURO-D Euro-Depression scale, SDQ Strengths and Difficulties Questionnaire, PRIME-MD Primary Care Evaluation of Mental Disorders, MSC Mental Component Summary, SF-12 12-item Short-Form health survey, K-6 Kessler Psychological Distress Scale - 6, DASS21 Depression and Anxiety Scale 21, K-10 Kessler Psychological Distress 10 item scale, WHO’s CIDI-Auto 2.1 World Health Organization’s Composite International Diagnostic Interview—Auto 2.1, SDQ Child Strengths and Difficulties Questionnaire PHQ-9 Patient Health Questionnaire 9. Quality assessment according to Newcastle-Ottawa criteria(9): High quality = *** Fair quality =** Low quality =*

**Table 3: Characteristics of studies with SARS exposure**

| **First Author** | **Quality** | **Year** | **Study design** | **Country** | **Main mental health outcome measure** | **Follow up period** |
| --- | --- | --- | --- | --- | --- | --- |
| Lai D W L(185) | ** | 2008 | Repeated cross-sectional | Hong Kong | 15-item Geriatric Depression Scale | 3-5 months dec 2002 - Jan 2003 and April-May 2003 |
| Cheung Y(186) | ** | 2008 | Longitudinal cohort | China | Suicide | 9 – 11 years 1993-2002/2004 |
| Yu H Y R(187) | ** | 2005 | Longitudinal cohort | Hong Kong | CES-D* | 1 year 2002-2003 |

**Table legend:** * CES-D Center for Epidemiologic Studies Depression. Quality assessment according to Newcastle-Ottawa criteria(9): High quality = *** Fair quality =** Low quality =*
