# Supplementary material for: Effects of the COVID-19 pandemic and previous pandemics, epidemics and economic crises on mental health: systematic review
Source: BJPsych Open. 2022 Oct 10;8(6):e181. doi: 10.1192/bjo.2022.587 (PMC9551492; doi:10.1192/bjo.2022.587)
Supplement: Supplementary file 1 [file S2056472422005877sup001.zip › S2056472422005877sup005.docx]

**Table 1: findings form individual studies in relation to country and study size**

|  | **Exposure to Covid-19**  **(Increased effect (n) / no change (n) / decreased effect (n))** | | | | **Exposure to economic crises**  **(Increased effect (n)/ no change (n) / decreased effect (n))** | | | | **Exposure to SARS**  **(Increased effect (n)/ no change (n)/ decreased effect (n))** | |
| --- | --- | --- | --- | --- | --- | --- | --- | --- | --- | --- |
|  | **Affective disorders** | **Suicides** | **Other mental health problems** | **Healthcare utilization** | **Affective disorders** | **Suicides** | **Other mental health problems** | **Healthcare utilization** | **Suicides** | **Other mental health problems** |
| **Regions^*^** |  |  |  |  |  |  |  |  |  |  |
| US/Canada | 8 / 3 / 0 | 0 / 0 / 1 | 8 / 4 / 0 | 0 / 0 / 1 | 8 / 1 / 1 | 4 / 0 / 0 | 2 / 0 / 0 | 1 / 0 / 0 | ---- | ---- |
| Europe & Central Asia | 14 / 2 / 2 | ---- | 12 / 4 / 0 | 4 / 0 / 3 | 1 / 1 / 0 | 27^#^ / 1 / 2^#^ | 16 / 3 / 0 | 5 / 0 / 0 | ---- | ---- |
| East Asia & Pacific | 7 / 0 / 2 | 0 / 2 / 0 | 2 / 0 / 0 | 1 / 0 / 1 | 3 / 0 / 0 | 2 / 0 / 0 | 2 / 0 / 0 | 1 / 0 / 0 | 2 / 0 / 0 | 1 / 0 / 0 |
| South Asia | 2 / 0 / 0 | ---- | ---- | ---- | ---- | ---- | ---- | ---- | ---- | ---- |
| Middle East & North Africa | 0 / 0 / 1 | ---- | ---- | ---- | ---- | ---- | ---- | ---- | ---- | ---- |
| Sub-Saharan Africa | 1 / 0 / 0 | ---- | ---- | ---- | ---- | ---- | ---- | ---- | ---- | ---- |
| Latin America & Caribbean | ---- | 0 / 0 / 1 | ---- | ---- | ---- | ---- | ---- | ---- | ---- | ---- |
| Multiregional | 1 / 0 / 0 | ---- | ---- | ---- | ---- | 2 / 0 / 0 | 1 / 1 / 0 | ---- | ---- | ---- |
| **Study size**** |  |  |  |  |  |  |  |  |  |  |
| Below 1000 | 19 / 4 / 3 | 0 / 0 / 1 | 8 / 4 / 0 | 3 / 0 / 2 | 0 / 1 / 0 | 0 / 0 / 0 | 0 / 0 / 0 | 0 / 0 / 0 | 2 / 0 / 0 | 0 / 0 / 0 |
| >1000 - ≤10,000 | 9 / 1 / 2 | 0 / 1 / 0 | 9 / 4 / 0 | 0 / 0 / 2 | 7 / 0 / 1 | 0 / 0 / 0 | 7 / 2 / 0 | 0 / 0 / 0 | 0 / 0 / 0 | 0 / 0 / 0 |
| More than 10,000 | 5 / 0 / 0 | 0 / 1 / 1 | 5 / 0 / 0 | 2 / 0 / 1 | 5 / 1 / 0 | 36 / 1 / 2 | 14 / 2 / 0 | 7 / 0 / 0 | 0 / 0 / 0 | 1/ 0 / 0 |

* Categorized according to World Bank regions (<https://data.worldbank.org/country>)

** Total number of study participants

^#^ The study is a multi-country study conducted in the EU, where overall increase in suicide rates was found, but in Austria rates were found to decrease – therefore the study appears twice in the table.

n = number of studies
